# ER-to-Golgi transport machinery promotes the excessive cargo-triggered unfolded protein response in *C*. *elegans*

**DOI:** 10.1371/journal.pgen.1012301

**Published:** 2026-09-08

**Authors:** Liying Guan, Tong Zhang, Zhigao Zhan, Yingchun Wang, Xun Huang, Mei Ding

**Affiliations:** 1 State Key Laboratory of Metabolism and Regulation in Complex Organization, Taikang Center for Life and Medical Sciences, School of Basic Medical Sciences, Wuhan University, Wuhan, China; 2 Institute of Genetics and Developmental Biology, Chinese Academy of Sciences, Beijing, China; 3 University of Chinese Academy of Sciences, Beijing, China; University of California San Diego, UNITED STATES OF AMERICA

## Abstract

Disruption of endoplasmic reticulum (ER) homeostasis activates the unfolded protein response (UPR) to restore proteostasis. Although defects in the secretory machinery can induce ER stress, whether specific trafficking components actively couple cargo handling to UPR signaling remains unclear. Here, using *Caenorhabditis elegans* genetics, imaging, and biochemical assays, we show that neuronal overexpression of the gap junction protein UNC-9 cell-autonomously activates the IRE-1-XBP-1 branch of the ER UPR. Loss of the early secretory pathway proteins ERGI-2 or ERGI-3 suppresses this response and disrupts UNC-9 localization, revealing functions for these proteins that extend beyond cargo trafficking. ERGI-2 and ERGI-3 interact with both UNC-9 and the ER chaperone HSP-4/BiP, suggesting that they couple the handling of excessive UNC-9 to UPR activation. This requirement is cargo-selective: ERGI-2 and ERGI-3 are dispensable for UPR activation induced by overexpression of another innexin, UNC-7, or unrelated proteins. Moreover, activation of the IRE-1-XBP-1 pathway reduces abnormal UNC-9 accumulation in *ergi-2* and *ergi-3* mutants. Together, our findings identify ER-to-Golgi trafficking proteins as cargo-selective regulators that link secretory-pathway demand to adaptive UPR.

## Author summary

Cells must carefully balance protein production, transport, and quality control. When proteins accumulate in the endoplasmic reticulum, cells activate a protective response that helps restore normal function. Although protein transport and stress signaling are closely connected, we still do not fully understand how the machinery that moves proteins through the cell influences this protective response. We used the nematode *Caenorhabditis elegans* to investigate what happens when neurons produce excessive amounts of UNC-9, a protein used to build communication channels between neighboring cells. We found that excess UNC-9 activates a protective stress response within the neurons producing it. Two proteins involved in transporting newly made proteins from the endoplasmic reticulum, ERGI-2 and ERGI-3, were required for both the normal distribution of UNC-9 and activation of this response. Their involvement was selective: they were not required for stress responses caused by excessive production of several other proteins. We also found that activating the protective response reduced abnormal UNC-9 accumulation. Our findings show that protein-transport machinery can help cells recognize and respond to the burden created by particular proteins. This connection may be especially important in long-lived cells such as neurons, which must maintain protein balance throughout life.

## Introduction

The endoplasmic reticulum (ER) serves as a central hub for protein folding, quality control, and secretion, overseeing the maturation of approximately one-third of the cellular proteome. Properly folded polypeptides are packaged into COPII-coated vesicles and exported to the Golgi apparatus, whereas misfolded proteins are retained and ultimately directed to ER-associated degradation (ERAD) and ER-phagy [1–3]. Perturbations in secretory trafficking, arising from genetic mutations, environmental insults, or elevated protein synthesis, can lead to aberrant protein aggregation and ER dysfunction [4]. To counteract such challenges, cells activate the unfolded protein response (UPR), a stress-adaptive signaling network that enhances folding capacity and clears aberrant proteins [5–8]. However, a critical question remains unanswered: are the components of the secretory machinery merely passively conduits that become overloaded during stress, or do they actively shape UPR signaling? While trafficking defects are well-established UPR inducers, whether transport regulators themselves modulate UPR output has remained largely unexplored.

In metazoans, the UPR is coordinated by three ER-resident transmembrane sensors: IRE1 (inositol-requiring enzyme 1), PERK (PKR-like ER kinase), and ATF6 (activating transcription factor 6) [9,10]. Among these, IRE1 is the most evolutionarily conserved. Upon ER stress, IRE1 oligomerizes and catalyzes the unconventional splicing of XBP1 mRNA, yielding the active transcription factor XBP1s [11–13]. This transcriptional program upregulates genes encoding ER chaperones and ER-phagy components, thereby enhancing the ER’s protein-folding capacity and promoting the clearance of misfolded proteins [14]. Despite extensive knowledge of UPR activation, the potential regulatory influence of secretory cargo burden, i.e., the flux of proteins traversing the ER-Golgi axis, on UPR dynamics has received little attention.

We previously reported that neuronal overexpression of UNC-9, a four-transmembrane gap junction protein, activates the IRE1-XBP1 branch of the ER UPR [15]. UNC-9 is broadly expressed in both neurons and muscle cells of *C. elegans*. *ergi-2* and *ergi-3* genes encode the *C. elegans* orthologs of yeast Erv41/Erv46 and mammalian ERGIC2/ERGIC3, respectively, which have been characterized as COPII-associated regulators of ER-to-Golgi transport [16–22]. Our previous study showed that, in body-wall muscle cells, loss of *ergi-2* or *ergi-3* causes UNC-9 to accumulate in the ER and impairs its delivery to cell-cell junctions [21]. Here, we found that neuronal localization of overexpressed UNC-9 similarly requires *ergi-2* and *ergi-3*. In the absence of either gene, neuronal UNC-9 aggregates within the ER and fails to be delivered to neuronal processes. Strikingly, however, this ER retention suppresses UPR activation. In other words, trapping the very cargo that normally induces stress prevents the stress response, even though the ER is filled with aggregated protein. This counterintuitive finding reveals that ERGI-2/ERGI-3 are not passive escorts but active promoters of UPR signaling. Genetic evidence places *ergi-2* and *ergi-3* upstream of *ire- 1* in this response. Moreover, ERGI-2 and ERGI-3 associate with both UNC-9 and the ER chaperone BiP/HSP-4, suggesting that these interactions may couple the handling of excessive cargo to UPR signaling. Together, our findings uncover a previously unrecognized mechanism by which protein-trafficking machinery links cargo load to stress-sensor activation. This model expands the conventional view of secretory cargo proteins as passive sources of ER stress by suggesting that specific cargo-trafficking interactions can actively shape ER UPR signaling, with potential relevance to ER stress-associated conditions, including neurodegenerative and metabolic diseases.

## Results

### *ergi-2* and *ergi-3* are required for the ER stress response induced by UNC-9 overexpression

**Fig 1:**
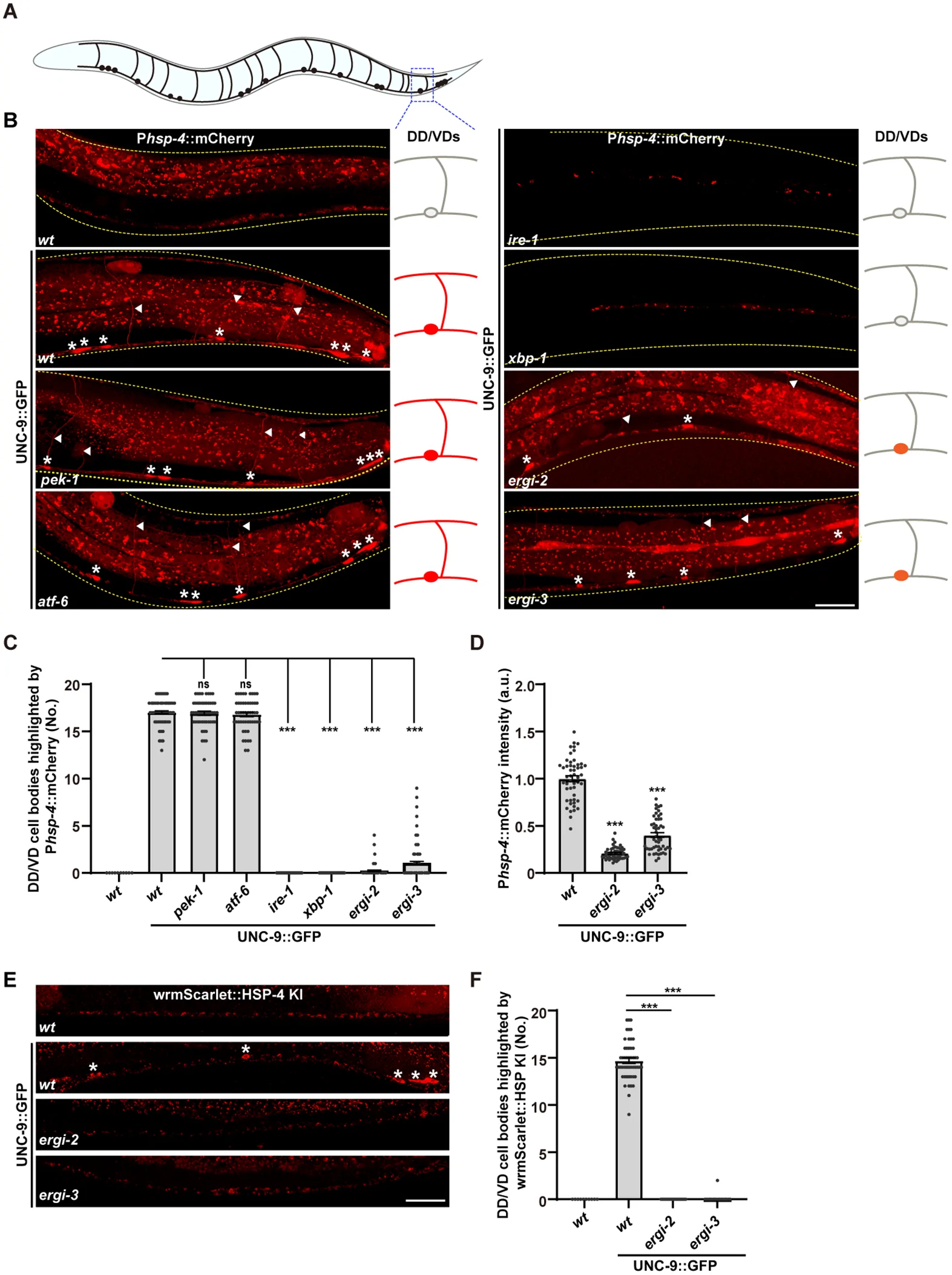
Loss of *ergi-2* or *ergi-3* suppresses UNC-9-induced UPR activation. **(A)** Schematic representation of DD/VD neurons in *C. elegans*. **(B)** UNC-9::GFP overexpression induces P*hsp-4*::mCherry expression (red) in DD/VD neurons, as shown by comparison with wild-type animals lacking UNC-9::GFP. This induction is reduced in *ire-*1, *xbp-1*, *ergi-2*, and *ergi-3* mutants, but not in *pek-1* or *atf-6* mutants. Yellow dashed lines outline the animals. White arrowheads indicate commissures, and asterisks indicate neuronal cell bodies. Scale bar, 25 μm. **(C)** Quantification of the number of mCherry-positive DD/VD neuronal cell bodies. **(D)** Quantification of mCherry fluorescence intensity in DD/VD neuronal cell bodies. a.u., arbitrary units. **(E)** UNC-9::GFP overexpression induces expression of endogenous wrmScarlet::HSP-4 (red) in DD/VD neurons, and this induction is reduced in *ergi-2* and *ergi-3* mutants. Asterisks indicate neuronal cell bodies. Scale bar, 25 μm. **(F)** Quantification of the number of wrmScarlet-positive DD/VD neuronal cell bodies. Data are presented as mean ± SEM; n ≥ 50 animals per genotype. Statistical significance was determined by one-way ANOVA followed by Tukey’s multiple-comparisons test. ***p < 0.001; ns, not significant.

In *C. elegans*, the D-type motor neurons (DD/VD) reside along the ventral nerve cord and extend commissural processes across the body to the dorsal cord, where they elaborate anteriorly and posteriorly projecting branches, respectively (Fig 1A, [23]). The gene *hsp-4* encodes the nematode ortholog of the ER chaperone BiP, a canonical transcriptional target upregulated upon ER stress [11]. Under basal conditions, an *hsp-4* promoter-driven mCherry reporter (P*hsp-*4::mCherry) exhibits weak, diffuse expression in intestinal and epidermal cells (Fig 1B, [15,24]). Upon DD/VD-specific expression of the gap junction protein UNC-9 under the control of the *Punc-25* promoter [25], we observed a pronounced increase in P*hsp-4::mCherry* fluorescence, which was strictly restricted to DD/VD neurons (Fig 1B, [15]). This result suggests that overexpression of UNC-9 triggers an ER stress response specifically in DD/VD neurons.

The UPR is orchestrated by three distinct signaling branches: the IRE1, PERK and ATF6 pathways [9]. Loss of *pek-1* (the worm PERK ortholog) or *atf-6* did not significantly alter *hsp-4* induction (Fig 1B and 1C). In contrast, mutations in *ire-1* or *xbp-1* markedly attenuated the *hsp-4* reporter signal (Fig 1B and 1C), indicating that the IRE1-XBP1 arm is specifically required for the UPR triggered by UNC-9 overexpression.

ERGI-2 and ERGI-3 were previously identified as key regulators involved in the ER-to-Golgi transport of UNC-9 in *C. elegans* muscle cells [21]. While examining whether *ergi-2* and *ergi-3* are required for the proper distribution of neuronally overexpressed UNC-9, we unexpectedly found that in both *ergi-2* and *ergi-3* mutant animals, the UNC-9-overexpression-induced upregulation of P*hsp-4*::mCherry was severely impaired (Fig 1B-1D). Both the proportion of mCherry-positive DD/VD neurons and the fluorescence intensity per neuron were markedly reduced compared to wild types (Fig 1B-1D). This diminished reporter expression was not attributable to neuronal loss, as the total number of DD/VD neurons remained unchanged in the mutants (S1A-S1B Fig). Expressing wild-type copy of *ergi-2* and *ergi-3* in DD/VD neurons significantly rescued the ER stress response triggered by UNC-9 overexpression (S1C-S1D Fig), indicating that *ergi-2* and *ergi-3* could act cell-autonomously in DD/VD neurons to modulate the ER stress response.

To validate these findings at the level of the endogenous locus, we generated a knock-in strain expressing wrmScarlet fused to *hsp-4* gene locus. Overexpression of UNC-9::GFP in this background robustly induced wrmScarlet::HSP-4 fluorescence specifically in DD/VD neurons (Fig 1E and 1F), confirming that excessive UNC-9 upregulates endogenous *hsp-4* transcription. Consistently, in *ergi-2* and *ergi-3* mutants, this induction was largely suppressed (Fig 1E and 1F). Together, these data demonstrate that *ergi-2* and *ergi-3* are essential mediators of the ER stress response elicited by UNC-9 overexpression in DD/VD neurons.

### *ergi-2* and *ergi-3* function upstream of *ire-1*

**Fig 2:**
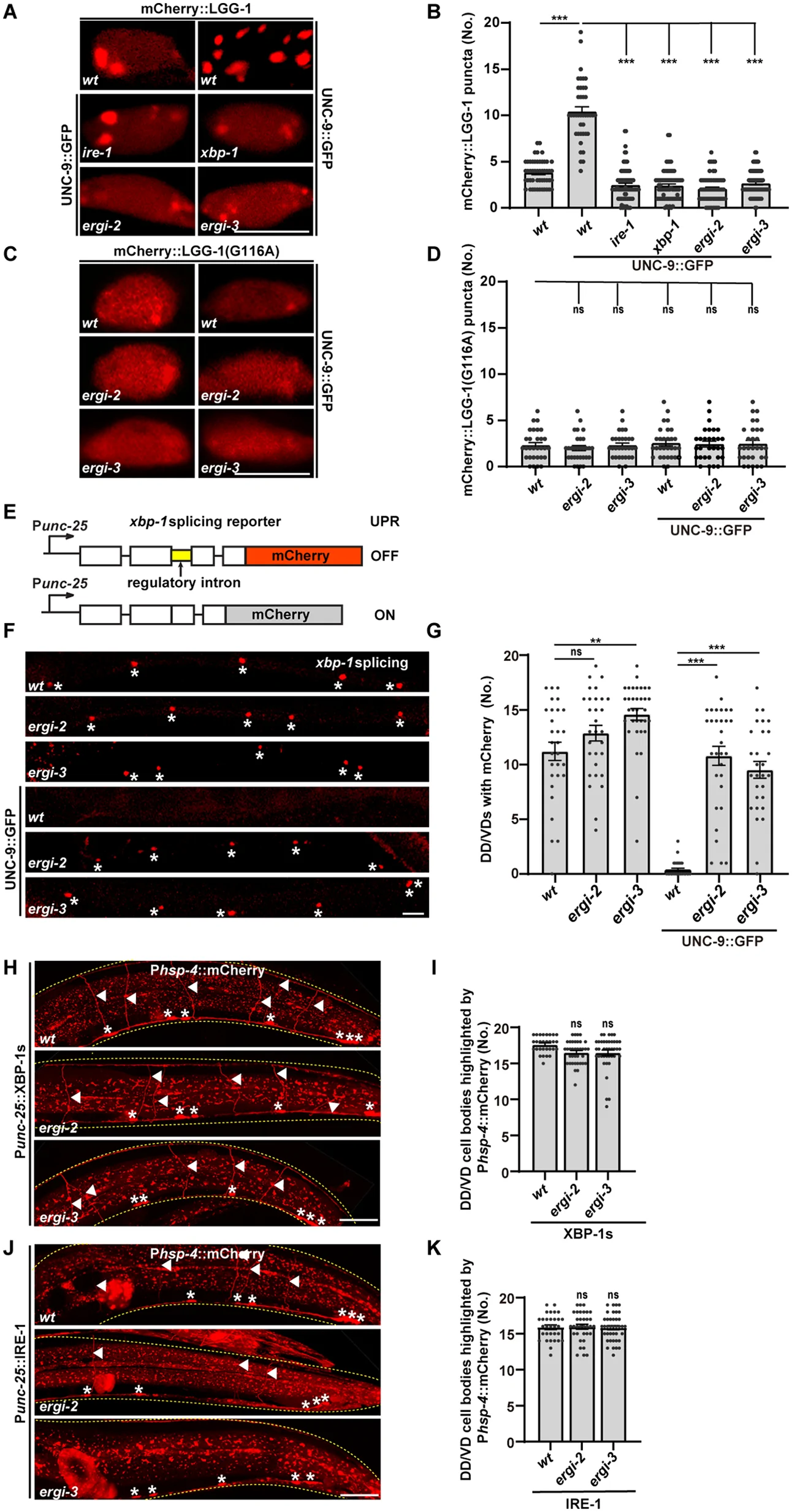
*ergi-2* and *ergi-3* function upstream of *ire-1* in UNC-9-induced UPR activation. **(A)** UNC-9::GFP overexpression induces the formation of mCherry::LGG-1 puncta (red), as shown by comparison with wild-type animals lacking UNC-9::GFP. This response is reduced in *ire-1*, *xbp-1*, *ergi-2*, and *ergi-3* mutants. Scale bar, 5 μm. **(B)** Quantification of the number of mCherry::LGG-1 puncta in DD/VD neuronal cell bodies shown in **(A)**. **(C)** The lipidation-defective mCherry::LGG-1(G116A) mutant (red) fails to form puncta following UNC-9::GFP overexpression in wild-type, *ergi-2*, or *ergi-3* animals, providing a specificity control for LGG-1 puncta formation. Scale bar, 5 μm. **(D)** Quantification of the number of mCherry::LGG-1(G116A) puncta in DD/VD neuronal cell bodies shown in **(C)**. **(E)** Schematic representation of the *xbp-1* splicing reporter. **(F)** Representative images of the *xbp-1* splicing reporter in the absence (top) or presence (bottom) of UNC-9::GFP in wild-type, *ergi-2*, and *ergi-3* animals. UNC-9::GFP-induced *xbp-1* splicing is reduced in *ergi-2* and *ergi-3* mutants. Asterisks indicate neuronal cell bodies. Scale bar, 25 μm. **(G)** Quantification of mCherry-positive DD/VD neuronal cell bodies shown in **(F)**. **(H)** XBP-1s overexpression induces P*hsp-4*::mCherry expression (red), and this induction is maintained in *ergi-2* and *ergi-3* mutants. **(I)** Quantification of mCherry-positive DD/VD neuronal cell bodies shown in **(H)**. **(J)** IRE-1 overexpression induces P*hsp-*4::mCherry expression (red), and this induction is maintained in *ergi-2* and *ergi-3* mutants. **(K)** Quantification of mCherry-positive DD/VD neuronal cell bodies shown in **(J)**. In **(H)** and **(J)**, white arrowheads indicate commissures, asterisks indicate neuronal cell bodies, and scale bars represent 25 μm. Data are presented as mean ± SEM; n ≥ 30 animals per genotype. Statistical significance was determined by one-way ANOVA followed by Tukey’s multiple-comparisons test. **p < 0.01; ***p < 0.001; ns, not significant.

Neuronal overexpression of UNC-9 selectively engages the IRE1-XBP1 branch of the UPR. In addition to inducing the canonical UPR target HSP-4, this pathway promotes autophagy, providing an independent readout of UPR activation, as we previously demonstrated [15]. To determine whether *ergi-2* and *ergi-3* act through the IRE1-XBP1 axis, we monitored autophagy activity using a DD/VD-specific mCherry::LGG-1 reporter (*C. elegans* Atg8) (Fig 2A and 2B). LGG-1(G116A) is a lipidation-deficient mutant of LGG-1 that fails to localize to membranes (Fig 2C, [26,27]). Under autophagy-activating conditions, if the wild-type mCherry::LGG-1 reporter forms puncta while the G116A reporter remains diffuse, this confirms that the observed puncta represent lipidation-dependent autophagosomal localization rather than non-specific aggregation caused by overexpression of the mCherry fusion protein [28,29]. Using both wild-type and G116A LGG-1 reporters, we examined the effect of UNC-9 overload and found that overexpression of UNC-9::GFP specifically caused the wild-type reporter mCherry::LGG-1 to form puncta, whereas the G116A reporter remained diffuse (Fig 2A and 2C). Hence, UNC-9 overload indeed creates an autophagy-activating condition. We further examined *ergi-2* and *ergi-3* mutants and found that in loss-of-function mutants of either gene, this autophagic response was largely abrogated, with puncta formation reduced to near-baseline levels (Fig 2A-2D). Thus, *ergi-2* and *ergi-3* are required for UNC-9::GFP-evoked autophagy, consistent with their involvement in IRE-1-XBP-1-dependent UPR signaling.

A hallmark of IRE-1 activation is the non-conventional splicing of *xbp-1* mRNA. To monitor this event specifically in DD/VD neurons, we developed an *in vivo* splicing reporter [15,30]. This reporter comprises mCherry fused to the un-spliced (inactive) isoform of *xbp-1*, expressed under the control of the DD/VD-specific P*unc-25* promoter (Fig 2E). In the absence of ER stress, the unspliced transcript permits mCherry expression (“mCherry on”). Upon IRE-1 activation, removal of a 23-nucleotide intron from *xbp-1* mRNA disrupts the mCherry open reading frame, resulting in loss of fluorescence (“mCherry off”) (Fig 2E). Consistent with our previous observations, UNC-9::GFP overexpression triggered efficient *xbp-1* splicing, as evidenced by the disappearance of mCherry signal in DD/VD neurons. In *ergi-2* and *ergi-3* mutants, however, UNC-9::GFP-induced splicing was markedly impaired, with persistent mCherry expression indicating a failure to engage the IRE-1-XBP-1 pathway (Fig 2F and 2G). These findings position *ergi-2* and *ergi-3* upstream of *xbp-1* splicing, implicating them in the initiation or propagation of IRE-1-dependent signaling. Notably, in the absence of UNC-9::GFP overexpression, loss of *ergi-2* or *ergi-3* alone did not elicit *xbp-1* splicing (Fig 2F and 2G), suggesting that their role in UPR activation is contingent upon elevated secretory cargo load.

We next asked whether *ergi-2* and *ergi-3* function at the level of IRE-1 or downstream effectors. Overexpression of either the constitutively active spliced isoform of XBP-1 (XBP-1s) or full-length IRE-1 in DD/VD neurons was sufficient to induce UPR activation, as monitored by the P*hsp-4*::mCherry reporter (Fig 2H-2K). However, this induction was unaffected in *ergi-2* or *ergi-3* mutants (Fig 2H-2K), indicating that neither XBP-1s nor activated IRE-1 requires *ergi-2/ergi-3* function to execute downstream UPR transcriptional programs. These results collectively suggest that ERGI-2 and ERGI-3 act upstream of IRE-1 activation, likely modulating the initiation of UPR signaling in response to cargo overload.

### The specific role of *ergi-2* and *ergi-3* in UPR activation

To further dissect the role of ERGI-2 and ERGI-3 in UPR activation, we asked whether the COPII machinery participates in the UNC-9-induced stress response. Using tissue-specific RNAi, we found that knockdown of core COPII components (*sec-12* or *sec-13*) in DD/VD neurons led to substantial neuronal loss, consistent with the essential function of COPII in viability (S2A and S2B Fig). In the surviving neurons, however, the intensity of the ER stress reporter P*hsp-4*::mCherry remained comparable to controls (S2C and S2D Fig). Thus, while COPII function is critical for neuronal survival, it is not required for the UPR elicited by UNC-9 overexpression.

We next examined whether other ER-Golgi intermediate compartment proteins contribute to this response. ERGIC32, which shares structural similarity with ERGIC2 and ERGIC3 and interacts with ERGIC3 to modulate its localization [31], is represented in *C. elegans* by *ergi-1*. In *ergi-1* mutant animals, UNC-9-induced UPR activation was indistinguishable from wild-type controls (S2E and S2F Fig). Similarly, loss of ERGIC53/*ile-1*, a cargo receptor critical for ER-Golgi transport [32,33], did not impair P*hsp-4*::mCherry induction (S2E and S2F Fig). Taken together, these observations suggest that ERGI-2 and ERGI-3 play a dedicated role in mediating the ER stress provoked by excessive UNC-9, operating independently of the core ER-Golgi trafficking machinery.

### *ergi-2* and *ergi-3* are selectively required for UPR induced by excessive UNC-9

**Fig 3:**
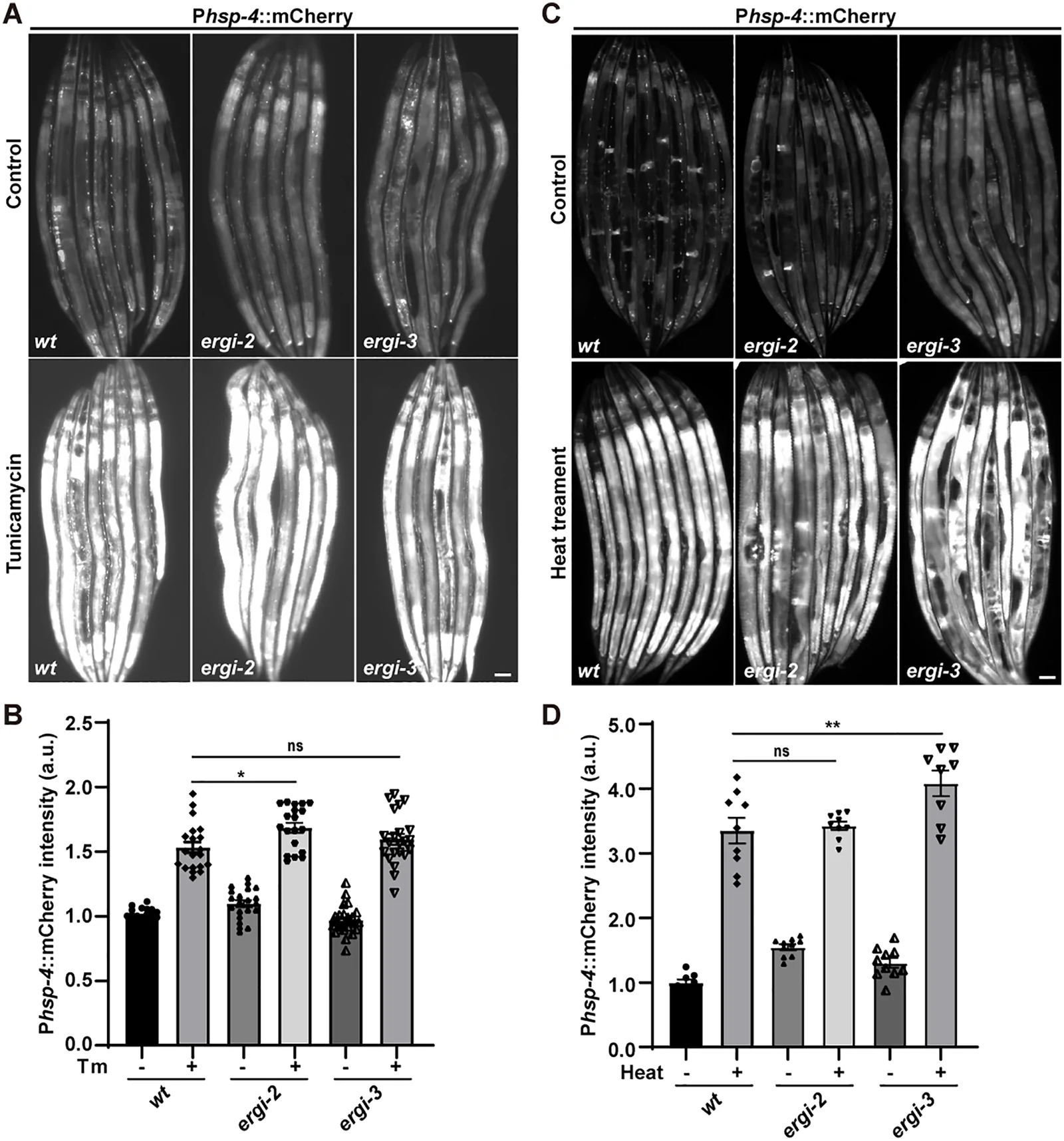
UPR induced by tunicamycin or heat stress is retained in *ergi-2* and *ergi-3* mutants. **(A)** Tunicamycin treatment induces P*hsp-4*::mCherry expression, and this response is not reduced in *ergi-2* or *ergi-3* mutants. **(B)** Quantification of P*hsp-4*::mCherry fluorescence intensity in the intestine in **(A)**; n ≥ 20 animals per genotype. **(C)** Heat treatment induces P*hsp-4*::mCherry expression, and this response is not reduced in *ergi-2* or *ergi-3* mutants. **(D)** Quantification of P*hsp-4*::mCherry fluorescence intensity in the intestine in **(C)**; n ≥ 8 animals per genotype. Scale bars, 25 μm in (A) and **(C)**. Data are presented as mean ± SEM. Statistical significance was determined by one-way ANOVA followed by Tukey’s multiple-comparisons test. *p < 0.05; **p < 0.01; ns, not significant.

To determine whether ERGI-2 and ERGI-3 function as general UPR mediators, we challenged mutants with tunicamycin (Tm), an N-linked glycosylation inhibitor that broadly disrupts protein folding and robustly activates the IRE-1/XBP-1 branch of the UPR [11,34]. Tm treatment induced strong P*hsp-4*::mCherry expression in the intestine, yet this response was fully preserved in *ergi-2* and *ergi-3* mutants (Fig 3A and 3B). Consistent with the Tm results, heat shock, a distinct proteotoxic stressor that likewise compromises protein folding [35,36], induced comparable levels of intestinal P*hsp-4*::mCherry in wild-type, *ergi-2*, and *ergi-3* animals (Fig 3C and 3D). This corroborates the view that ERGI-2 and ERGI-3 are not required for the UPR activated by protein misfolding *per se*.

**Fig 4:**
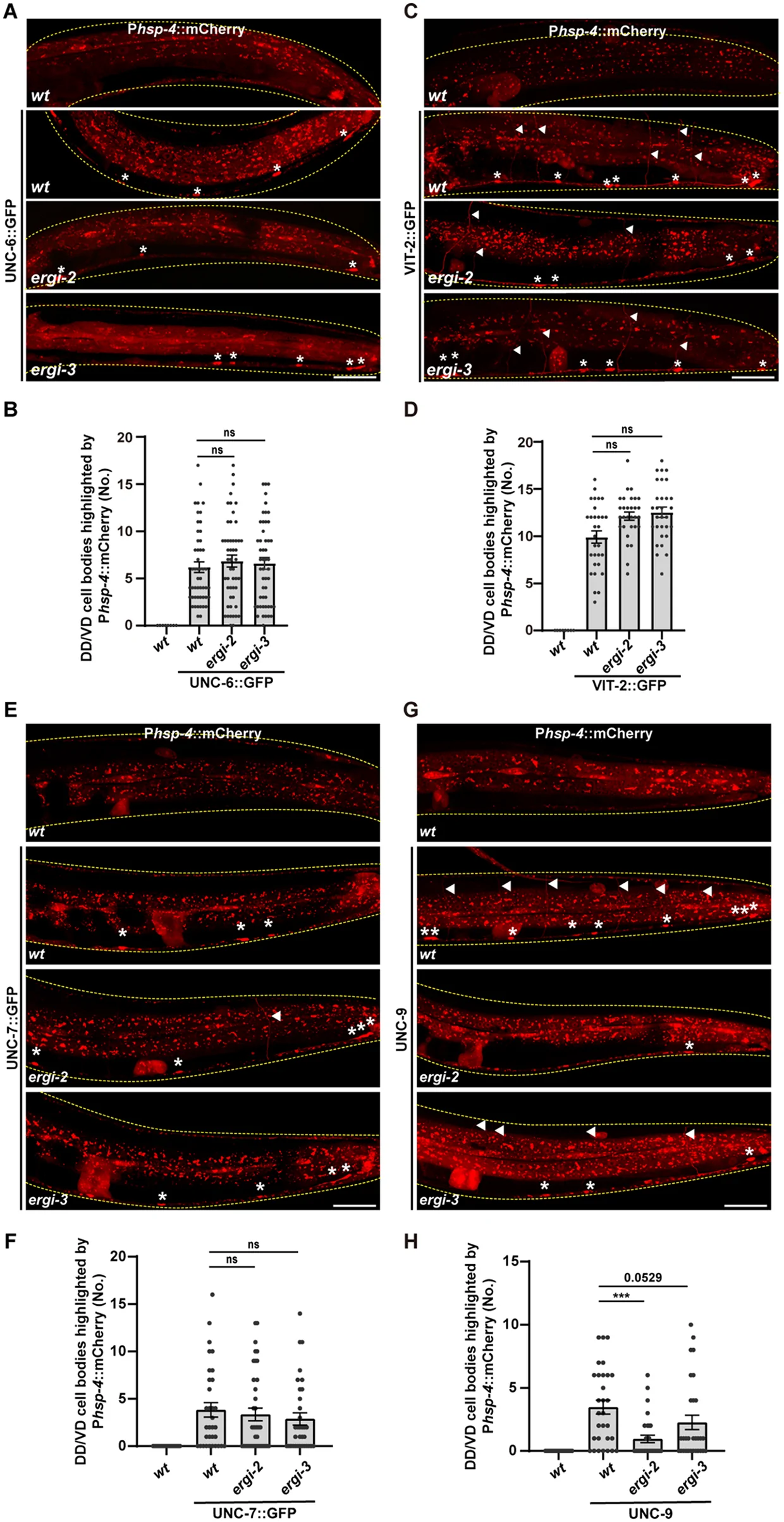
ERGI-2 and ERGI-3 are selectively required for UPR induced by UNC-9 overexpression. **(A)** Overexpression of UNC-6::GFP in DD/VD neurons induces P*hsp-4*::mCherry expression, and this response is not suppressed in *ergi-2* or *ergi-3* mutants. **(B)** Quantification of P*hsp-4*::mCherry-positive DD/VD neuronal cell bodies shown in **(A)**. **(C)** Overexpression of VIT-2::GFP in DD/VD neurons induces P*hsp-4*::mCherry expression, and this response is not suppressed in *ergi-2* or *ergi-3* mutants. **(D)** Quantification of P*hsp-4*::mCherry-positive DD/VD neuronal cell bodies shown in **(C)**. **(E)** Overexpression of UNC-7::GFP in DD/VD neurons induces P*hsp-4*::mCherry expression, and this response is not suppressed in *ergi-2* or *ergi-3* mutants. **(F)** Quantification of P*hsp-4*::mCherry-positive DD/VD neuronal cell bodies shown in **(E)**. **(G)** Overexpression of untagged UNC-9 in DD/VD neurons induces P*hsp-4*::mCherry expression. This induction is markedly reduced in *ergi-2* mutants and moderately reduced in *ergi-3* mutants. **(H)** Quantification of P*hsp-4*::mCherry-positive DD/VD neuronal cell bodies shown in **(G)**. Yellow dashed lines outline the animals. White arrowheads indicate commissures, and asterisks indicate neuronal cell bodies. Scale bars, 25 μm in **(A)**, **(C)**, **(E)**, and **(G)**. Data are presented as mean ± SEM; n ≥ 30 animals per genotype. Statistical significance was determined by one-way ANOVA followed by Tukey’s multiple-comparisons test. ***p < 0.001; ns, not significant.

Having excluded a general role in UPR activation, we asked whether these genes might specifically mediate neuronal UPR caused by overexpression of misfolded or secreted cargo. We turned to two distinct secretory proteins: UNC-6/Netrin, an evolutionarily conserved guidance cue, and VIT-2, a yolk protein involved in lipid transport [37,38]. Overexpression of either UNC-6::GFP or VIT-2::GFP in DD/VD neurons led to marked upregulation of P*hsp-4*::mCherry, confirming UPR induction (Fig 4A-4D). However, mutations in *ergi-2* or *ergi-3* failed to suppress this activation (Fig 4A-4D), indicating that these genes are not broadly required for UPR arising from neuronal protein overexpression.

To further test whether the role of ERGI-2 and ERGI-3 in regulating ER export is specifically linked to their function in UNC-9-overload-induced UPR, we examined UNC-7, another innexin protein similarly expressed in neurons and muscle cells in *C. elegans* [39,40]. When GFP-tagged UNC-7 was expressed in GABAergic neurons, we found that it significantly increased the transcriptional level of *hsp-4*, indicating UPR activation (Fig 4E-4F). However, this upregulation of *hsp-4* transcription was not suppressed when *ergi-2* or *ergi-3* was mutated (Fig 4E-4F).

We were concerned that the UPR effects observed with *ergi-2* and *ergi-3* might actually stem from an abnormal GFP fusion protein of UNC-9, rather than from UNC-9 itself. To address this, we reevaluated our findings using untagged UNC-9. Overexpression of untagged UNC-9 in DD/VD neurons was still sufficient to specifically activate *hsp-4* expression in these cells (Fig 4G-4H). This induction was significantly reduced by the *ergi-2* mutation, while the *ergi-3* mutation also produced an apparent reduction that did not reach statistical significance (*p* = 0.0529). The concordant effects observed with tagged and untagged UNC-9 support the conclusion that UNC-9 itself activates the UPR and that this response is promoted by ERGI-2 and ERGI-3, rather than being an artifact of the GFP fusion (Fig 4G-4H).

Together, these results demonstrate that the UPR triggered by UNC-9 overexpression depends specifically on ERGI-2 and ERGI-3. Importantly, this dependence is not due to a general role in the UPR, but rather points to a substrate-tailored surveillance mechanism. In this model, ERGI-2 and ERGI-3 act not as core UPR components, but as selective facilitators that enable the cellular response to particular misfolded clients.

### Excessive UNC-9 accumulates in the ER in e*rgi-2* and *ergi-3* mutants

**Fig 5:**
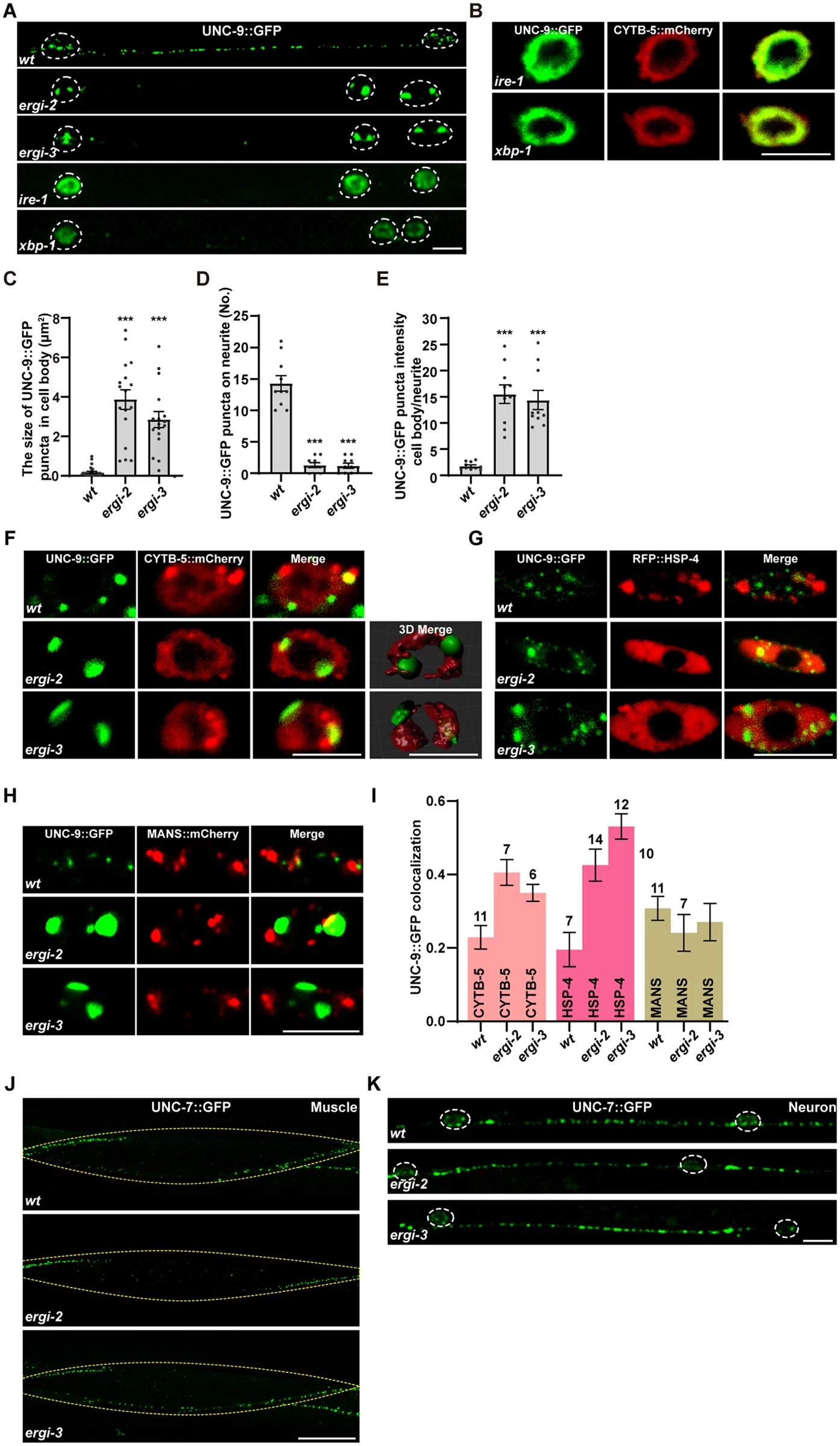
Large UNC-9::GFP aggregates accumulate in the ER in *ergi-2* and *ergi-3* mutants. **(A)** UNC-9::GFP distribution in DD/VD neuronal cell bodies and neurites in the indicated genotypes. White dashed circles outline neuronal cell bodies. **(B)** UNC-9::GFP colocalization with the ER marker CYTB-5::mCherry in *ire-1* and *xbp-1* mutants. **(C-E)** Quantification of UNC-9::GFP puncta size in cell bodies **(C)**, puncta number in neurites **(D)**, and the cell body-to-neurite fluorescence-intensity ratio **(E)**. **(F)** Co-localization between large UNC-9::GFP aggregates and CYTB-5::mCherry in *ergi-2* and *ergi-3* mutants. **(G)** Small UNC-9::GFP dots colocalize with RFP::HSP-4 in *ergi-2* and *ergi-3* mutants. **(H)** UNC-9::GFP shows little colocalization with the Golgi marker MANS::mCherry in *ergi-2* and *ergi-3* mutants. **(I)** Pearson’s correlation coefficients for UNC-9::GFP colocalization with the ER markers CYTB-5 and HSP-4 and the Golgi marker MANS. Sample sizes are indicated above the bars. **(J)** P*myo-3*::UNC-7::GFP forms puncta at body-wall muscle cell-cell junctions, and this distribution is unchanged in *ergi-2* and *ergi-3* mutants. Yellow dashed lines outline the muscle cells. Scale bar, 25 μm. **(K)** P*unc-25*::UNC-7::GFP forms puncta in DD/VD neuronal cell bodies and processes, and this distribution is unchanged in *ergi-2* and *ergi-3* mutants. White dashed circles outline neuronal cell bodies. Scale bars, 5 μm in **(A)**, **(B)**, **(F)**, **(G)**, **(H)**, and **(K)**. Data are presented as mean ± SEM; n ≥ 10 animals per genotype. Statistical significance was determined by one-way ANOVA followed by Tukey’s multiple-comparisons test. ***p < 0.001; ns, not significant.

Our previous study showed that ERGI-2 and ERGI-3 bind UNC-9 and promote its ER-to-Golgi transport [21]. To understand the selective role of *ergi-2* and *ergi-3* in the UPR induced by excessive UNC-9, we examined the subcellular localization of UNC-9 in DD/VD neurons. In wild-type animals, ectopically expressed UNC-9::GFP formed distinct puncta along neurites and within cell bodies (Fig 5A, [15]). In *ire-1* or *xbp-1* mutants, the punctate UNC-9::GFP signal became diffusely distributed in the ER of DD/VD cells, accompanied by a marked reduction of punctate signals along neurites (Fig 5A-5B, [15]), suggesting that the UPR is required for proper distribution of excessive UNC-9 proteins. In contrast, mutations in *ergi-2* and *ergi-3* produced a distinctly different phenotype, characterized by the formation of large UNC-9::GFP blobs within the DD/VD cell body region (Fig 5A, 5C). Concomitantly, the UNC-9::GFP signal along DD/VD neurites was greatly diminished (Figs 5A, 5D-5E). Despite these localization defects, UNC-9::GFP expression levels were not altered in either *ergi-2* or *ergi-3* mutants (S3A Fig).

Since ERGI-2 and ERGI-3 regulate ER-to-Golgi transport of UNC-9 in muscle cells [21], we wondered in which organelle these enlarged structures reside in DD/VD neurons. Double-labeling with the ER marker CYTB-5::mCherry revealed that the large UNC-9::GFP plaques in the mutants appear to localize within, or on the membranes of, the ER (Fig 5F). However, the large aggregates formed in the *ergi-2* and *ergi-3* mutants prevented us from obtaining a definitive answer. Interestingly, the large UNC-9::GFP aggregates resolved into smaller puncta when co-expressed with the ER marker HSP-4. These smaller signals co-localized extensively with RFP::HSP-4 (Fig 5G), indicating that in the absence of ERGI-2 or ERGI-3, overexpressed UNC-9 becomes trapped within the ER lumen. Meanwhile, UNC-9 colocalization with the Golgi was unchanged (Fig 5H-5I). Together with its increased ER accumulation, this finding supports the conclusion that ER-to-Golgi trafficking of UNC-9 is impaired in *ergi-2* and *ergi-3* mutants, resulting in its retention in the ER [21].

ERGI-2 and ERGI-3 are not required for the UPR induced by overexpression of UNC-7 (Fig 4E-4F), another innexin protein. To further test whether the unique role of ERGI-2 and ERGI-3 in UNC-9-overload-induced UPR is linked to their function in UNC-9 transport, we examined the subcellular localization of UNC-7. In wild-type muscle cells, UNC-7::GFP displayed a punctate pattern at cell-cell junctions. This distribution was unaltered in *ergi-2* or *ergi-3* mutants (Fig 5J). Similarly, in DD/VD neurons, UNC-7::GFP formed puncta distributed in both cell bodies and neuronal processes, and this pattern also remained unchanged in the absence of ERGI-2 or ERGI-3 (Fig 5K).

Thus, unlike UNC-7, which requires neither ERGI-2 nor ERGI-3 for its proper localization or for the UPR triggered by its overexpression (Fig 5J-5K), UNC-9 depends on ERGI-2 and ERGI-3 for both its distribution and the UPR induced by its overexpression (Fig 5A, 5C-5E). These findings indicate that the selective role of ERGI-2 and ERGI-3 in activating the UPR in response to UNC-9 overload stems from their specific requirement for UNC-9 cargo transport, a function not shared by UNC-7.

### ERGI-2 and ERGI-3 facilitate UPR activation by modulating the BiP/IRE-1 interaction

**Fig 6:**
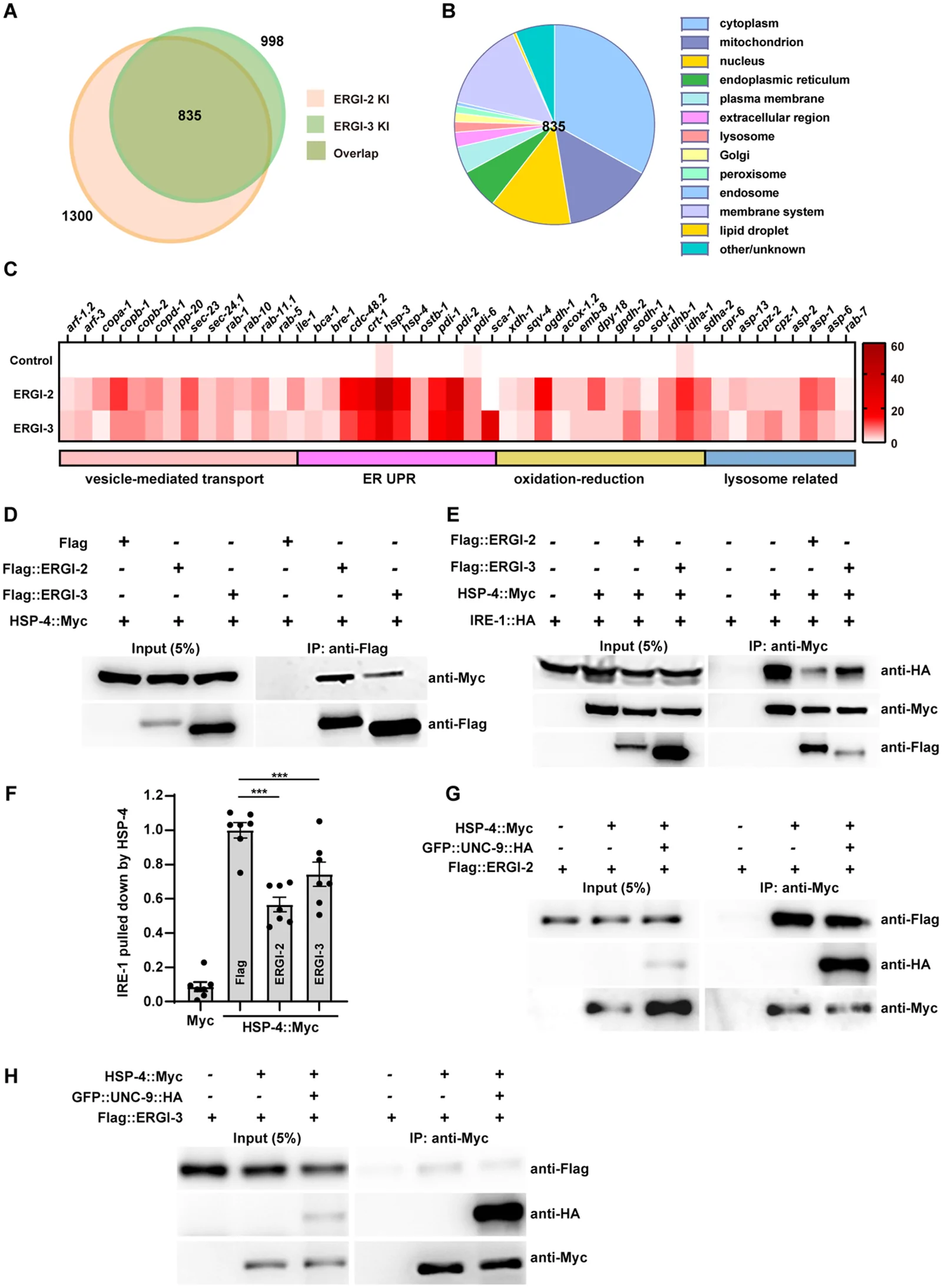
ERGI-2 and ERGI-3 reduce the association between HSP-4 and IRE-1. **(A)** Venn diagram of proteins identified by immunoprecipitation-mass spectrometry as interactors of GFP::ERGI-2 (1,300 proteins) or GFP::ERGI-3 (998 proteins), including 835 shared interactors. **(B)** Gene Ontology cellular-component analysis of the 835 shared interactors. **(C)** Heatmaps of representative shared interactors. Wild-type animals expressing GFP served as controls, and GFP::ERGI-2 and GFP::ERGI-3 knock-in strains were used for immunoprecipitation-mass spectrometry. The color scale indicates peptide-spectrum matches. **(D)** Co-immunoprecipitation of HSP-4::Myc with Flag::ERGI-2 or Flag::ERGI-3. **(E)** Co-immunoprecipitation of IRE-1::HA with HSP-4::Myc in the presence or absence of Flag::ERGI-2 or Flag::ERGI-3. **(F)** Quantification of IRE-1::HA co-immunoprecipitated with HSP-4::Myc, normalized to immunoprecipitated HSP-4::Myc and input IRE-1::HA. Data are presented as mean ± SEM; n = 7. Statistical significance was determined by one-way ANOVA followed by Tukey’s multiple-comparisons test. ***p < 0.001; ns, not significant. **(G, H)** Co-immunoprecipitation assays showing associations among HSP-4::Myc, UNC-9::GFP, and Flag::ERGI-2 (G) or Flag::ERGI-3 **(H)**.

To further dissect the mechanisms underlying ERGI-2 and ERGI-3 function in UPR activation, we performed an unbiased proteomic approach to identify their interacting partners. Using CRISPR/Cas9, we generated endogenous GFP knock-in strains for ERGI-2 and ERGI-3 and performed anti-GFP immunoprecipitation coupled with mass spectrometry (IP-MS). This analysis revealed a substantial overlap between the interactomes of ERGI-2 and ERGI-3 (Fig 6A and 6B), corroborating our previous finding that they function as a complex [21]. As anticipated, both proteins associated with components of COPII and COPI vesicles, consistent with their established role in early secretory pathway trafficking (Fig 6C). Notably, their interactomes were also enriched for proteins involved in ER UPR, redox homeostasis, and lysosomal degradation. Among these, HSP-4/BiP, a master regulator of the unfolded protein response, emerged as a particularly compelling candidate (Fig 6C).

We validated these interactions through co-immunoprecipitation (Co-IP) assays in HEK293T cells, demonstrating that both Flag-tagged ERGI-2 and ERGI-3 bind to Myc-tagged HSP-4 (Fig 6D). Reciprocal Co-IP using anti-Myc antibodies further confirmed this association (S4A Fig). Notably, ERGI-2 exhibited stronger binding to HSP-4 than did ERGI-3, suggesting that ERGI-3 may associate with HSP-4 indirectly through ERGI-2. This interaction appears evolutionarily conserved, as human ERGIC2 and ERGIC3 similarly co-precipitated with human BiP (S4B Fig). Domain mapping revealed that both the nucleotide-binding domain (NBD) and the substrate-binding domain (SBD) of HSP-4 [41,42] can independently interact with ERGI-2; however, full-length HSP-4 exhibited the strongest binding (S4A Fig), indicating that the intact structural conformation of HSP-4 is required for optimal interaction.

How does the BiP/HSP-4 interaction participate in ERGI-2/ERGI-3-mediated UPR signaling? Cumulative evidences indicate that IRE-1 activation is contingent upon the dissociation of BiP [43–46]. Therefore, we tested whether ERGI-2/ERGI-3 might modulate the BiP-IRE-1 interaction. Co-IP experiments revealed that expression of either ERGI-2 or ERGI-3 substantially reduced the binding of HSP-4::Myc to IRE-1::HA (Fig 6E and 6F). Consistent with its higher affinity for HSP-4, ERGI-2 exerted a more pronounced effect. Neither ERGI-2 nor ERGI-3 alone bound directly to IRE-1 (S4C Fig), suggesting that their modulatory function is mediated through HSP-4 rather than through direct competition for IRE-1 binding.

However, the ability of ERGI-2 and ERGI-3 to bind HSP-4 alone cannot explain their apparent specificity for the UPR induced by UNC-9 overexpression. Neuronal overexpression of either *ergi-2* or *ergi-3* alone did not induce the UPR (S5A-S5B Fig). Moreover, overexpression of *ergi-2* or *ergi-3* did not enhance the UPR triggered by UNC-6::GFP or VIT-2::GFP overexpression (S5A-S5B Fig). Co-immunoprecipitation experiments further demonstrated that HSP-4, ERGI-2, and UNC-9 can coexist in the same protein complex (Fig 6G and H). Collectively, these findings suggest that the involvement of ERGI-2 and ERGI-3 in UPR activation is determined primarily by their cargo-specific association with UNC-9 rather than by a general ability to regulate HSP-4 or IRE-1.

### Restoring UPR activation alleviates UNC-9 aggregation

**Fig 7:**
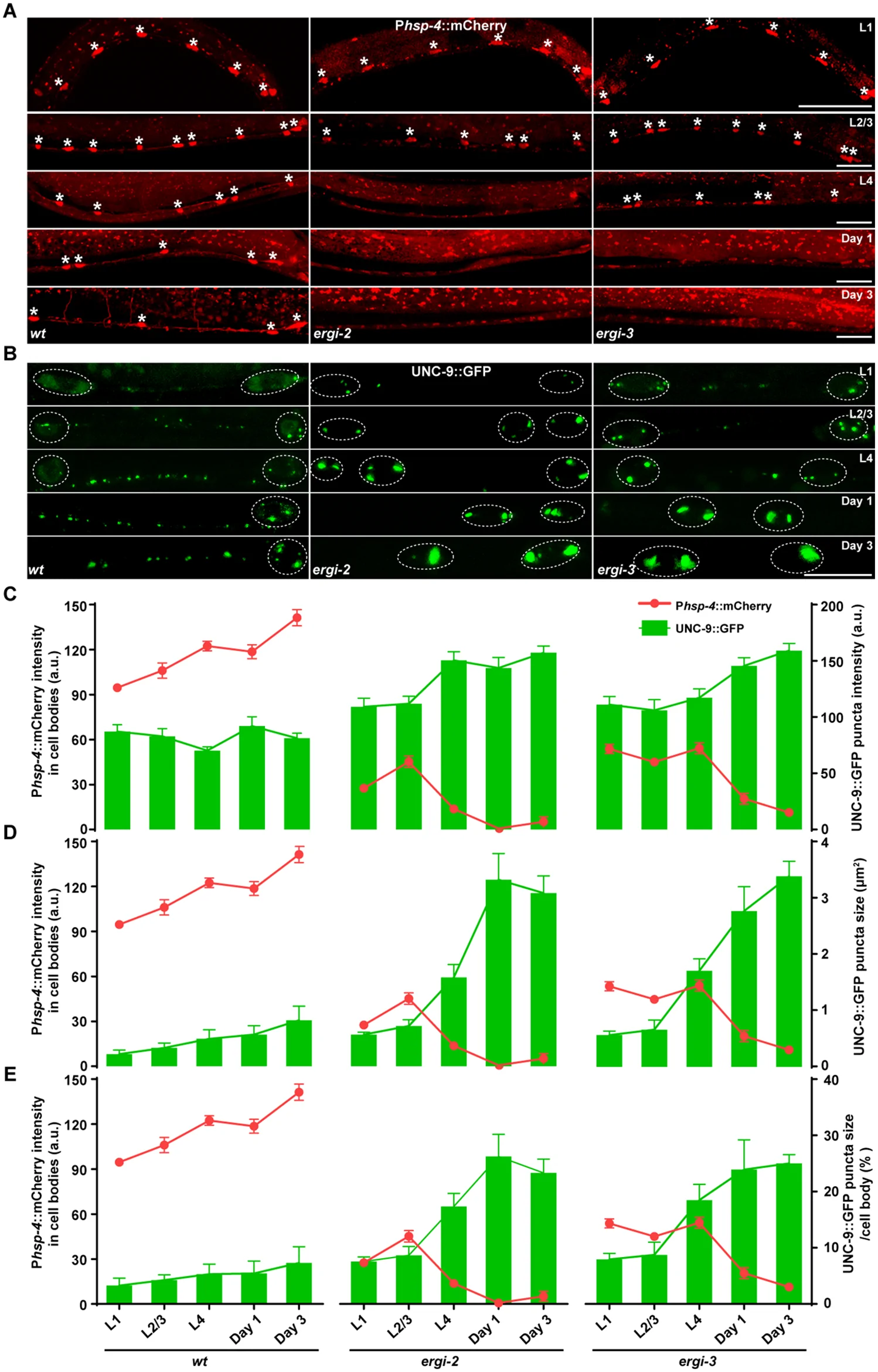
Progressive UNC-9::GFP aggregation is accompanied by reduced UPR activity. **(A)** P*hsp-4*::mCherry expression (red) in DD/VD neurons at the indicated developmental stages in wild-type, *ergi-2*, and *ergi-3* animals. Asterisks indicate DD/VD neuronal cell bodies. Scale bar, 25 μm. **(B)** UNC-9::GFP distribution in DD/VD neurons across the same developmental stages and genotypes. Dashed lines outline neuronal cell bodies. Scale bar, 10 μm. **(C-E)** Quantification of P*hsp-4*::mCherry expression and UNC-9::GFP puncta across developmental stages. Red lines, corresponding to the left y-axis, show P*hsp-4*::mCherry fluorescence intensity in DD/VD neuronal cell bodies; green bars, corresponding to the right y-axis, show UNC-9::GFP puncta intensity **(C)**, puncta area **(D)**, and the percentage of the cell-body area occupied by puncta **(E)**. In *ergi-2* and *ergi-3* mutants, UNC-9::GFP aggregation increases during development as P*hsp-4*::mCherry expression declines. a.u., arbitrary units. Data are presented as mean ± SEM; n ≥ 10 animals per genotype.

Despite the eventual suppression of UPR in *ergi-2* and *ergi-3* mutants, we observed that UPR activation remained detectable during early larval development. P*hsp-4*::mCherry signal, marking UPR in DD/VD neuron cell bodies, was present from the L1 through L3 stages, albeit at lower intensity than in wild-type animals (Fig 7A). During this period, UNC-9::GFP maintained a punctate distribution comparable to that of wild-type controls (Fig 7B). By the L4 stage, however, P*hsp-4*::mCherry expression in DD/VD neurons had markedly declined (Fig 7A and 7C-7E). This attenuation of UPR signaling coincided precisely with the loss of UNC-9::GFP puncta from neurites and the appearance of enlarged perinuclear blobs (Fig 7A-7E). These temporal correlations suggest that ERGI-2 and ERGI-3 are required to sustain UPR activity, which in turn prevent the aberrant aggregation of UNC-9.

**Fig 8:**
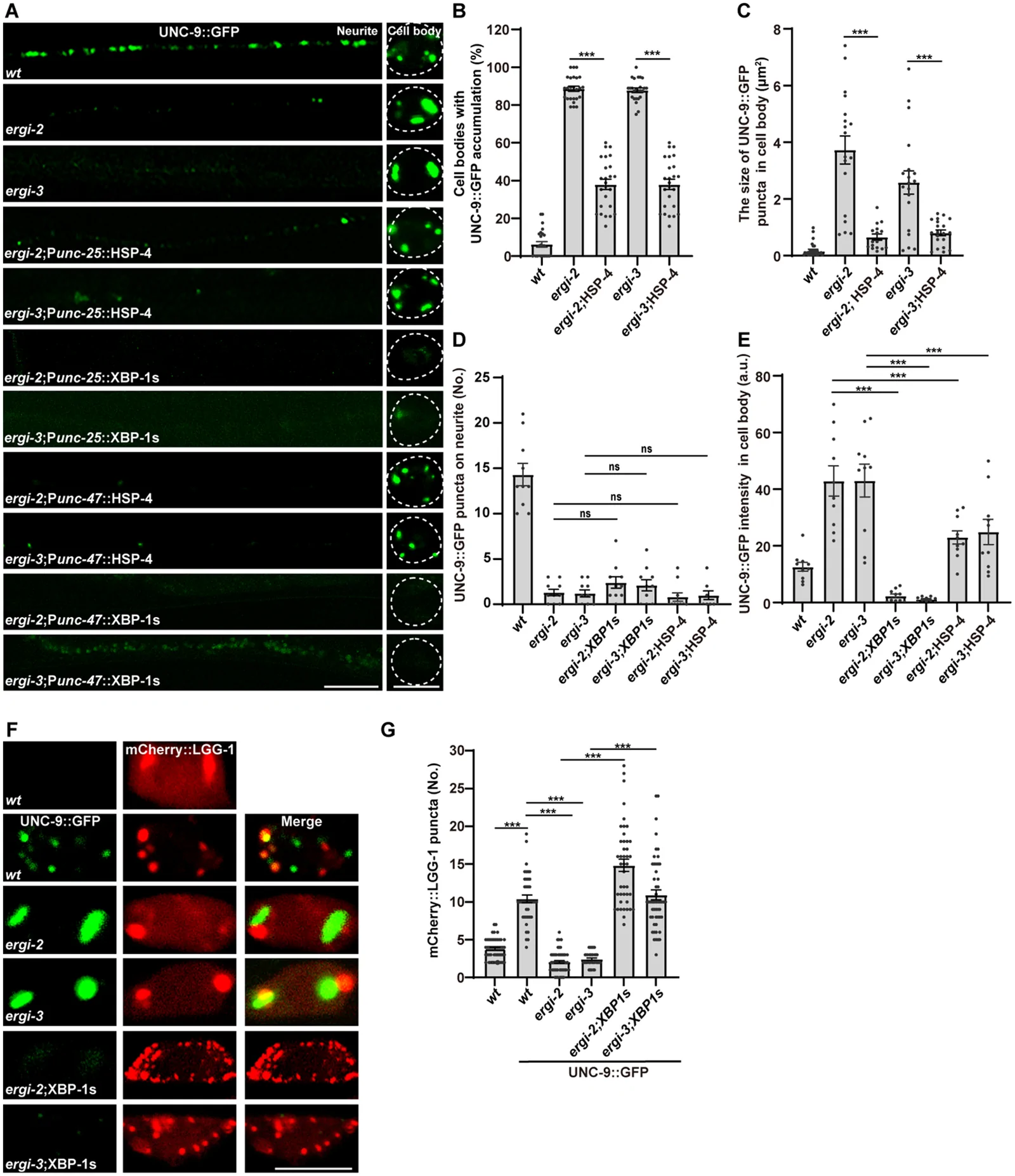
Enhancing UPR activity alleviates abnormal UNC-9::GFP accumulation in *ergi-2* and *ergi-3* mutants. **(A)** UNC-9::GFP (green) accumulates abnormally in the cell bodies of *ergi-2* and *ergi-3* mutants. Neuron-specific expression of HSP-4 or spliced XBP-1 (XBP-1s), driven by the P*unc-25* or P*unc-47* promoter, reduces this accumulation. Left panels, neuronal processes; right panels, cell bodies. Scale bars, 5 μm. **(B-E)** Quantification of UNC-9::GFP distribution: the percentage of neuronal cell bodies exhibiting abnormal accumulation **(B)**, puncta area in cell bodies **(C)**, puncta number in neuronal processes **(D)**, and fluorescence intensity in cell bodies **(E)**. **(F)** Expression of P*unc-25*::XBP-1s increases mCherry::LGG-1 puncta formation (red) and reduces UNC-9::GFP aggregation (green). Scale bar, 5 μm. **(G)** Quantification of mCherry::LGG-1 puncta in DD/VD neuronal cell bodies. Data are presented as mean ± SEM; n ≥ 10 animals per genotype. Statistical significance was determined by one-way ANOVA followed by Tukey’s multiple-comparisons test. ***p < 0.001; ns, not significant.

To test this hypothesis directly, we asked whether restoring UPR signaling could reverse or remodel UNC-9 aggregation in *ergi-2* and *ergi-3* mutants. As the IRE-1-XBP-1 pathway orchestrates both chaperone induction and autophagy activation, we first examined whether augmenting chaperone capacity alone, by overexpressing HSP-4 in DD/VD neurons, could resolve the UNC-9::GFP blobs. Remarkably, HSP-4 overexpression significantly reduced both the size of enlarged UNC-9::GFP puncta and the total UNC-9::GFP fluorescence intensity (Fig 8A-8E). Consistent with this observation, our earlier co-localization studies confirmed that RFP::HSP-4 expression markedly diminished UNC-9::GFP aggregate size (Fig 5G). These results indicate that replenishing ER chaperone capacity is sufficient to mitigate protein aggregation, even in the absence of *ergi-2/ergi-3* function.

We noticed that the ER-retained UNC-9::GFP aggregates in *ergi-2* or *ergi-3* mutants were often observed in close proximity to degradative organelles, including autophagosomes (labeled by mCherry::LGG-1), early endosomes (labeled by mCherry::RAB-5), late endosomes (labeled by mCherry::RAB-7), and lysosomes (labeled by mCherry::LAAT-1) (S6A-S6B Fig). This spatial association raises the possibility that the UPR-activated protein degradation pathways might be suppressed or inefficient in *ergi-2* or *ergi-3* mutants. In addition, spliced form of XBP-1 (XBP-1s) not only drives *hsp-4* transcription but also potentiates autophagy. Therefore, we assessed whether XBP-1s overexpression could similarly suppress UNC-9 aggregation. Indeed, expression of XBP-1s in DD/VD neurons nearly abolished UNC-9::GFP aggregation, with a near-complete loss of detectable fluorescence signal (Fig 8A, 8D-8E). Consistent with a role for autophagy in this clearance, XBP-1s overexpression also restored autophagic activity in both *ergi-2* and *ergi-3* mutant animals (Fig 8F and 8G).

To rule out the possibility that excessive amount of P*unc-25* promoter dampens UNC-9 expression, we used the P*unc-47* promoter which could also drive gene expression specifically in DD/VD neurons [47,48]. We found that P*unc-47*::XBP-1s or P*unc-47*::HSP-4 could also reduce the UNC-9::GFP aggregates formed in *ergi-2* and *ergi-3* mutant animals (Fig 8A). Hence, it is indeed due to the expression of XBP-1s or HSP-4, but not the potential promoter competition, cause the reduction of protein aggregates.

It is important to note, however, that neither XBP-1s nor HSP-4 overexpression restored the normal punctate distribution of UNC-9::GFP along DD/VD neurites (Fig 8A and 8D). This finding underscores that ERGI-2/ERGI-3-mediated ER-to-Golgi transport is an independent and essential process for proper UNC-9 localization, a function that cannot be bypassed merely by enhancing the UPR.

Taken together, these results establish a dual role for ERGI-2 and ERGI-3 in maintaining ER proteostasis: they not only facilitate the forward trafficking of secretory cargo but also promote IRE-1-XBP-1-mediated UPR activation, which in turn clears potentially toxic protein aggregates through chaperone upregulation and autophagy induction.

## Discussion

It is generally accepted that failure of the secretory pathway results in the abnormal accumulation of cargo proteins within ER, which in turn activates the UPR to resolve protein aggregates [6,7]. Here, our findings demonstrate that the early secretory components ERGIC2 and ERGIC3 are themselves directly involved in UPR activation upon cargo overload, revealing a dynamic and previously unappreciated coupling between protein trafficking and stress signaling.

### The dual role of ERGIC2 and ERGIC3 in proteostasis

The bifunctional nature of ERGIC2 and ERGIC3 offers a unique opportunity to dissect the intricate relationship between cargo transport and UPR activation. Under basal conditions, properly folded cargo proteins are efficiently exported from the ER [1,49], and the UPR remains quiescent, as it is not required for normal cargo localization. In this context, ERGIC2 and ERGIC3 bind correctly folded cargo and facilitate their ER exit [21]. However, during cargo overload, whether due to misfolding or excessive synthesis, the burden saturates ER chaperones such as BiP, displacing them from stress sensors like IRE1 and thereby initiating the UPR [50]. Under such conditions, the UPR becomes essential for the proper handling and localization of excess cargo [30,37]. Because protein folding is a prerequisite for ER export, the cargo-binding function of ERGIC2 and ERGIC3 positions them spatiotemporally at the nexus of UPR activation. During cargo overload, these factors not only engage cargo but also interact with BiP/HSP-4, contributing to its dissociation from IRE1. In this manner, overloaded cargo cooperates with ERGIC2 and ERGIC3 to amplify UPR signaling, ensuring that adaptive stress responses are precisely tuned to secretory demand.

Why are core COPII components dispensable for this cargo-triggered UPR? We propose that COPII deficiency causes a global accumulation of diverse ER-resident and secretory proteins, which may broadly titrate BiP away from ER stress sensors, thus activating the UPR in a non-specific manner. In contrast, overload of a specific cargo appears to require dedicated transport machinery to locally potentiate UPR activation. Given that selective protein accumulation, rather than stochastic aggregation, is a hallmark of many human diseases, particularly neurodegenerative disorders [10,51], targeting cargo-specific transporters may offer a more refined therapeutic strategy. Moreover, in conditions where such transporters are deficient, activating rather than inhibiting the UPR could represent an alternative approach to clear toxic aggregates and mitigate disease progression.

In *ergi-2* and *ergi-3* mutants, large protein aggregates form adjacent to lysosomes and autophagosomes, suggesting a failure in clearance mechanisms. Notably, restoring UPR signaling through XBP-1s overexpression efficiently resolves these aggregates, underscoring the critical role of the UPR in protein quality control. This observation aligns with studies showing that XBP-1s upregulates lysosomal activity [52] and that HSP70-family chaperones actively disassemble neurotoxic aggregates [53,54]. Our work thus positions ERGIC2 and ERGIC3 as gatekeepers of proteostasis, ensuring that cargo overload triggers adaptive UPR activation rather than toxic accumulation.

### ERGIC2 and ERGIC3 couple UNC-9 transport to UPR activation in a context-dependent manner

While defects in COPII-mediated transport are known to induce ER stress and activate the UPR [6,7], we now show that ERGIC2 and ERGIC3 are not merely passive cargo transporters but are critical regulators of the IRE1-XBP1 pathway. This finding suggests that components of the early secretory machinery can directly sense cargo burden and modulate UPR signaling accordingly. Intriguingly, proteomic analyses of purified ERGIC membranes have consistently identified BiP as an abundant resident [31,55], and a previous SEC-23B-centric COPII proteome similarly recovered BiP peptides [56,57]. This recurrent detection of BiP in early secretory compartments supports the notion that ERGIC- and COPII-associated machinery may cooperate to regulate BiP homeostasis, providing independent biochemical evidence for a functional link between the early secretory pathway and ER chaperone control.

Notably, the role of ERGI-2 and ERGI-3 in UPR activation is contingent upon cargo load. In the absence of excessive cargo burden, UPR activation does not require these factors, suggesting that cargo itself, or its transport, engages ERGI-2 and ERGI-3 to facilitate BiP dissociation from IRE-1. Consistent with this model, ERGI-2 exhibits a stronger influence on UPR activation than ERGI-3, correlating with its higher binding affinity for BiP/HSP-4. This functional divergence mirrors observations in mammalian cells, where ERGIC2 and ERGIC3 differentially regulate gap junction transport [21], and in yeast, where Erv41 and Erv46 play distinct roles in cargo binding [22]. We therefore propose that ERGIC3 primarily mediates cargo recognition, while ERGIC2 additionally serves as a UPR modulator—a dual role that ensures efficient coupling of secretory demand to stress resolution.

Our findings support a model in which the functions of ERGI-2 and ERGI-3 in UPR signaling are specifically linked to their handling of excessive UNC-9, rather than to a general role in ER stress signaling. In GABAergic neurons, loss of either protein causes overexpressed UNC-9 to accumulate abnormally in cell bodies, impairs its delivery to neuronal processes, and suppresses activation of the IRE-1-XBP-1 pathway. Thus, intracellular UNC-9 accumulation alone is insufficient to trigger the UPR. Instead, ERGI-2/3-dependent handling of excessive UNC-9 appears necessary to couple cargo overload to UPR activation.

Several observations argue that this response is not caused by abnormal behavior of the UNC-9::GFP fusion protein. UNC-9::GFP can reach cell-cell junction regions in muscle cells and can be transported from neuronal cell bodies into neuronal processes, demonstrating that it remains competent for secretory transport. More importantly, overexpression of untagged UNC-9 also induces *hsp-4* expression in DD/VD neurons. This response is markedly reduced in *ergi-2* mutants and modestly reduced in *ergi-3* mutants, consistent with the results obtained using UNC-9::GFP. UPR activation is therefore driven by excessive UNC-9 itself rather than by the GFP tag.

The selective requirement for ERGI-2 and ERGI-3 also cannot be explained by the *unc-25* promoter, GFP expression, or a general protein-overexpression burden. Expression of UNC-6::GFP or VIT-2::GFP from the same promoter activates *hsp-4* expression, but these responses are independent of ERGI-2 and ERGI-3. Moreover, overexpression of ERGI-2 or ERGI-3 does not further enhance the responses induced by these proteins, and *unc-9::gfp* transcript abundance is unchanged in *ergi-2* and *ergi-3* mutants. GABAergic neuron-specific expression of *ergi-2* or *ergi-3* restores UNC-9-induced *hsp-4* expression in the corresponding mutant, further supporting a cell-autonomous role within the neurons experiencing UNC-9 overload.

Comparison with UNC-7 provides particularly strong evidence for cargo selectivity. Like UNC-9, UNC-7 is an innexin whose overexpression activates the UPR in GABAergic neurons. However, loss of *ergi-2* or *ergi-3* neither suppresses UNC-7-induced UPR activation nor disrupts UNC-7::GFP localization in neurons or muscle cells. The requirement for ERGI-2 and ERGI-3 therefore closely parallels their cargo specificity: these proteins regulate both the localization of excessive UNC-9 and the UPR induced by it but are dispensable for the corresponding processes involving UNC-7.

The relationship among ERGI-2/3, UNC-9 trafficking, and UPR activation is also strongly shaped by cellular context and cargo abundance. In body-wall muscle cells, ERGI-2 and ERGI-3 are required for UNC-9 trafficking and gap junction formation [21], yet UNC-9 overexpression does not detectably activate the UPR. In neurons, by contrast, the localization of endogenous UNC-9 is independent of *ergi-2*, *ergi-3*, *ire-1*, and *xbp-1* (S7A-S7B Fig, [15]), whereas overexpressed UNC-9 requires ERGI-2/3 and IRE-1-XBP-1 branch for both its proper distribution and the accompanying UPR activation. Thus, ERGI-2 and ERGI-3 have a constitutive trafficking role for endogenous UNC-9 in muscle but acquire a detectable trafficking and stress-signaling role in neurons specifically under conditions of UNC-9 overload. These differences indicate that the cellular response to UNC-9 is determined by the combined effects of cargo identity, expression level, and cell-type-specific proteostatic capacity.

ERGI-2 and ERGI-3 associate with both UNC-9 and HSP-4/BiP, potentially placing them at an interface between cargo trafficking and stress signaling. Nevertheless, their interaction with HSP-4 is unlikely to confer a general role in the UPR, because they are dispensable for responses induced by UNC-7, UNC-6, and VIT-2. We therefore propose that, when UNC-9 production exceeds the normal handling capacity of GABAergic neurons, ERGI-2 and ERGI-3 coordinate its trafficking with activation of the IRE-1-XBP-1 pathway. More broadly, the early secretory pathway may collaborate with UPR regulators to accommodate fluctuations in protein synthesis, folding, and secretion in a cargo-, abundance-, and cell-type-dependent manner. Future studies comparing different cargoes and cellular contexts will be required to define the molecular basis and broader physiological relevance of this coordination *in vivo*.

### Perspective and future directions

While this study provides mechanistic insight into the role of ERGIC2 and ERGIC3 in coupling cargo transport to UPR activation, several important questions remain open for future investigation.

First, gap junction proteins are widely expressed across human tissues. Our findings establish that *ergi-2* and *ergi-3* modulate UPR triggered by neuronal UNC-9 overload. An important next step will be to determine whether their mammalian homologs function similarly in response to the overexpression of gap junction proteins in other tissues or in disease models characterized by gap junction dysregulation.

Second, cargo-receptor pairing is complex and often extends beyond a simple one-to-one relationship. A more systematic mapping of these specific interactions will help clarify how distinct secretory cargos engage the UPR. Additionally, other members of the ERGIC family, such as ERGIC53 and ERGIC32, are known to be essential for the efficient secretion of specific cargos under certain conditions [31,58–60], even though they are dispensable for bulk flow. It will be valuable to examine whether these related receptors similarly modulate the UPR in response to overload of their respective cargos.

Third, cellular context strongly shapes the response to cargo overload, indicating that the early secretory pathway collaborates with UPR regulators to dynamically manage fluctuations in protein synthesis, folding, and secretion across different cell types and physiological states. Future work should aim to elucidate the precise molecular mechanisms underlying this coordination *in vivo*, including comparative analyses across diverse cell types and tissues.

Fourth, it remains to be determined whether the UPR-modulating function of ERGIC2 and ERGIC3 is limited to a subset of cargo proteins or represents a more general mechanism for integrating secretory load with stress signaling. Expanding the repertoire of cargos examined, both in *C. elegans* and in mammalian systems, will help define the scope and generality of this regulatory axis.

In summary, our work reframes the secretory machinery as an active modulator of UPR signaling, rather than a passive target. By integrating cargo sensing, HSP-4/BiP regulation, and IRE-1 activation, ERGIC-2 and ERGIC-3 provide a mechanistic link between secretory demand and cellular stress resilience. This model offers a promising framework for understanding diseases associated with ER dysfunction, such as neurodegeneration, where defects in cargo handling and UPR dysregulation are prominent pathological features.

## Materials and methods

### Worm strains

Worm strains were maintained on nematode growth medium (NGM) plates at 22°C unless otherwise specified. Culture and genetic manipulation were carried out according to standard procedures [61]. The strains used in this study are listed in S1 Table.

### DNA constructs and transgenes

PCR amplifications were performed with KOD-Plus-Neo polymerase (TOYOBO, Cat. #KOD-401) or 2 × TransStart FastPfu Fly PCR SuperMix (TransGen, Cat. #AS231–01). Amplicons were cloned into pSM, ΔpSM, pPD95.77, or pPD95.75 vectors via Gibson assembly using the pEASY-Uni Seamless Cloning and Assembly Kit (TransGen Biotech).

Transgenic strains were generated by germline microinjection of the target construct (5–100 ng/μL) with the following co-injection markers: P*myo-2*::RFP (5 ng/μL), P*odr-1*::RFP (50 ng/μL), or P*odr-1*::GFP (50 ng/μL). Extrachromosomal arrays were integrated into the genome by trimethylpsoralen/ultraviolet (TMP/UV) mutagenesis [62]. Resulting integrants were outcrossed at least three times against the appropriate wild-type background.

### CRISPR/Cas9-mediated gene editing

Knock-in alleles were generated by CRISPR/Cas9-directed homologous recombination. The *xdKi80* (*gfp::ergi-2*) and *xdKi81*(*gfp::ergi-3*) alleles were generated by SunyBiotech and verified by PCR followed by sequencing. The *xdKi97(wrmScarlet::hsp-4)* allele was engineered using protocols optimized by the Jorgensen laboratory and similarly verified. The targeting sequences and genotyping primers are listed below.

Allele-specific reagents
  *xdKi80* (GFP::ERGI-2):
    sgRNA: 5′-GCTCGTCGGAAATCCGACAACGG-3′
    Forward primer: 5′-TGTAACATTAATATCACCCCAC-3′
    Reverse primer: 5′-AAGGGCATTCTGCAATTAGGA-3′
  *xdKi81* (GFP::ERGI-3):
    sgRNA 1: 5′-CCAATGGACGACTTTCGGGTCAA-3′
    sgRNA 2: 5′-GAAAGCCAATGGACGACTTTCGG-3′
    Forward primer: 5′-AGAGCAAAGGGCTAACTTAG-3′
    Reverse primer: 5′-GCAAGTACAAGTTGACCATAG-3′
  *xdKi97* (wrmScarlet::HSP-4):
    sgRNA 1: 5′-ACAGTACGCGTTTGCCACGA-3′
    sgRNA 2: 5′-GAAACGCCCAATCAGACGCT-3′
    Forward primer: 5′-CAGCCACTCAGCGAACAGTT -3′
    Reverse primer: 5′-TCCGGATTGATTGTGAGCTG -3′

### Neuron-specific RNAi

To achieve neuron-specific knockdown, we generated dsRNA from genomic DNA fragments of the target gene coding sequences (0.57-1.2 kb) under the control of the DD/VD neuron-specific promoter P*unc-25* [63,64]. The promoter and targeting sequences (50 ng/µL) were amplified and co-injected with the co-injection marker P*myo-2*::RFP (50 ng/µL). The following primers were used to amplify the fragments for *sec-12* and *sec-13* RNAi constructs, which were then recombined into the pPD49.26 plasmid:

*sec-12* front fragment:
  Forward primer: CAGGAGGACCCTTGGCTAGCCCAGCTTATTGTCTCAAAAC
  Reverse primer: GATATCAATACCATGGTACCCAAATCGAACGCATTTCTGA
*sec-12* rear fragment:
  Forward primer: CAGGAGGACCCTTGGCTAGCCAAATCGAACGCATTTCTGAC
  Reverse primer: GATATCAATACCATGGTACCCCAGCTTATTGTCTCAAAAC
*sec-13* front fragment:
  Forward primer: CAGGAGGACCCTTGGCTAGCTGGTATGGAATCGAGGATTTT
  Reverse primer: GATATCAATACCATGGTACCCAAATTGTGATCTGCAAAATTG
*sec-13* rear fragment:
  Forward primer: CAGGAGGACCCTTGGCTAGCCAAATTGTGATCTGCAAAAT
  Reverse primer: GATATCAATACCATGGTACCTGGTATGGAATCGAGGATTT

### Tunicamycin treatment

Synchronized L4-stage animals were incubated in M9 buffer containing 25 µg/ml Tunicamycin or an equivalent volume of DMSO (1% v/v final concentration) for 4 hours at 22 °C with gentle agitation. After two washes with M9, worms were transferred to OP50-seeded NGM plates and allowed to recover for 4 hours at 22 °C to permit maximal induction of P*hsp-4*::mCherry.

### Heat-shock treatment

Day 1 adult worms were collected and subjected to heat shock at 30 °C for 5 hours; control animals were maintained at 22 °C. After treatment, worms were immobilized on OP50-seeded NGM plates with 140nM sodium azide, oriented anterior-up under white light, and imaged using a Zeiss Imager A1 microscope controlled by ZEN software (Zeiss).

### Image collection and quantitative analysis

Worms were mounted on 2.5% agar pads in M9 containing 1.4% 1-phenoxy-2-propanol as anesthetic. All fluorescence images were acquired on a Leica SP8 confocal microscope unless otherwise stated. Imaging was performed at the young adult stage unless specified. Confocal stacks were projected to a single image. Comparative images of P*hsp-4*::mCherry strains were acquired using a 40 × objective, whereas UNC-9::GFP strains were imaged using either a 40× or 63 × objective. All images used for comparisons were acquired using identical settings and analyzed using the same thresholds and size criteria. Particle-size measurements, fluorescence-intensity quantification, and colocalization analyses using Pearson’s correlation coefficient were performed in ImageJ (NIH).

Specific quantitative analyses were performed as follows. DD/VD cell bodies were identified by their characteristic positions along the ventral nerve cord and were defined as approximately oval regions with an area of 6–20 μm^2^. To assess UPR activation in DD/VD neurons, we used the *hsp-4* transcriptional reporter P*hsp-4*::mCherry and quantified the numbers of mCherry-positive neuronal cell bodies, an mCherry-positive cell body was defined as an DD/VD cell body with a mean grayscale intensity greater than 50. For image quantification, we also measured the mean P*hsp-4*::mCherry fluorescence intensity within neuronal cell bodies and normalized it to that of the corresponding wild-type control.

UPR Induction in Intestine: P*hsp-4*::mCherry fluorescence induced by tunicamycin or heat shock was quantified and normalized to control levels.

Endogenous HSP-4 activation: Using the wrmScarlet::HSP-4 knock-in reporter, we quantified the numbers of fluorescently labeled neuronal cell bodies.

DD/VD neuron populations: The number of DD/VD neurons was quantified by counting P*unc-25*::mCherry-labeled somata.

*xbp-1* splicing activity: Using the P*unc-25*::XBP-1us::mCherry construct, we counted DD/VD cell bodies highlighted with mCherry signal.

UNC-9::GFP distribution in DD/VD neurons: In strains expressing P*unc-2*5::UNC-9::GFP, an individual UNC-9::GFP punctum was defined as a round particle with an area greater than 0.02 μm^2^ and a mean grayscale intensity greater than 40. The following parameters were quantified: (1) puncta intensity, defined as the mean fluorescence intensity of each UNC-9::GFP punctum; (2) puncta number, determined by counting UNC-9::GFP puncta along the neurite segment between the DD5 and VD9 cell bodies, or between the DD4 and DD5 cell bodies at the L1 stage; and (3) puncta size, defined as the area of each UNC-9::GFP punctum. Puncta with an area greater than 1 μm^2^ were classified as aggregates. For each neuronal cell body, the areas of all UNC-9::GFP puncta were summed and normalized to the total cell-body area to calculate the percentage of the soma occupied by UNC-9::GFP puncta. Mean UNC-9::GFP fluorescence intensity was also measured within defined regions of interest in the cell body (elliptical region, 12 μm^2^) and neurite (rectangular region, 30 × 0.35 μm). The cell body-to-neurite fluorescence-intensity ratio was then calculated.

Endogenous UNC-9 distribution: We used the UNC-9::split-GFP Native and Tissue-specific Fluorescence (NATF) approach to label endogenous UNC-9 (UNC-9-spGFP) [65], combined with P*unc-25*::mCherry to identify DD/VD neurons. The proportion of cell bodies with UNC-9-spGFP signals was quantified.

Autophagic activity: We generated a P*unc-25*::mCherry::LGG-1 reporter and quantified the number of mCherry::LGG-1 puncta with a diameter of at least 0.2 μm within DD/VD neuronal cell bodies in young adult animals.

### Immunoprecipitation for mass spectrometry analysis

Worm lysates were prepared from the strains *xdKi80* (ERGI-2::GFP) and *xdKi81* (ERGI-3::GFP), using GFP-only expressing worms as a control. Worms were collected and lysed using a Dounce homogenizer in pre-chilled homogenizing buffer (50 mM Tris-Cl, pH 8.0; 150 mM NaCl; 0.5% sodium deoxycholate; 1% Triton X-100; protease inhibitor [Roche]). Lysates were incubated for 15 minutes on ice and centrifuged at 12,000 × g for 15 minutes at 4°C. The supernatants were incubated with a GFP polyclonal antibody (rabbit, Abcam, ab290, 1:1,000 dilution) overnight, followed by incubation with protein A agarose beads (Cat#17-0780-01, GE) for 4 hours at 4°C. The pellets were washed three times with washing buffer (50 mM Tris-Cl, pH 8.0; 150 mM NaCl; 1% NP-40; 1 mM PMSF) and boiled in SDS sample buffer for 5 minutes. The boiled samples were resolved by SDS-PAGE, and protein bands of interest were excised. In-gel digestion was performed using a standard protocol (Shevchenko et al., 2006) with slight modifications. Proteins were reduced with 10 mM DTT, alkylated with 55 mM iodoacetamide, and digested overnight with sequencing-grade trypsin (Sigma) at 37°C. The resulting peptides were analyzed using a TripleTOF 5600 mass spectrometer (AB SCIEX) coupled to an Eksigent nanoLC. Peptide identification and quantification were performed with ProteinPilot 4.2 software (AB SCIEX) using the *C. elegans* proteome sequences (Uniprot) as a database and a mass tolerance of 0.05 Da. The false discovery rate (FDR) was analyzed using the integrated PSPEP software.

Proteins were selected based on significantly elevated or unique Peptide-Spectrum Matches (PSMs) in *xdKi80* and *xdKi81* samples compared to the GFP control. Candidate proteins were subjected to GO enrichment analysis using KOBAS. Functional clusters were visualized via hierarchical clustering and heatmaps (GraphPad Prism 8.0), with color intensity representing relative abundance.

### Immunoprecipitation and western blotting

HEK293T cells were maintained in DMEM supplemented with 10% FBS (Hyclone) at 37°C in 5% CO_2_. Transfections were performed using Lipofectamine 2000 (Invitrogen) according to the manufacturer’s instructions. Cells were harvested 24–48 hours post-transfection. For immunoprecipitation, whole-cell extracts were lysed in RIPA buffer (1% Triton X-100, 100 mM Tris-HCl, 50 mM EDTA, 150 mM NaCl, 1% deoxycholate, 0.1% SDS, and protease inhibitor cocktail) for 30 minutes at 4°C. Lysates were centrifuged at 12,000 × g for 15 minutes at 4°C. The supernatants were incubated with anti-FLAG M2 affinity gel or anti-Myc gel beads overnight at 4°C, washed three times with washing buffer, and eluted with SDS-sample buffer. Immunoprecipitated samples or whole-cell lysates were resolved by SDS-PAGE and transferred to nitrocellulose membranes (Pall Life Sciences). Membranes were blocked with 5% non-fat dried milk, and signals were detected using Pierce ECL western blotting substrate (Thermo Fisher Scientific, 34095). All co-immunoprecipitation experiments were repeated at least three times.

### Quantification of protein interactions

To quantitatively assess how ERGI-2 and ERGI-3 modulate the IRE-1::HA and HSP-4::MYC interaction, we used four conditions: (i) Myc-empty vector + IRE-1::HA (negative control), (ii) HSP-4::MYC + IRE-1::HA + empty Flag vector (basal control), (iii) HSP-4::MYC + IRE-1::HA + Flag::ERGI-2, and (iv) HSP-4::MYC + IRE-1::HA + Flag::ERGI-3. Protein band intensities were quantified using ImageJ. HSP-4::MYC immunoprecipitation (IP) efficiency was calculated as the anti-Myc IP/input ratio. IRE-1::HA co-IP efficiency was determined by normalizing anti-HA co-IP signals to the corresponding HSP-4::MYC IP values, adjusting for IP efficiency coefficients, and normalizing against IRE-1 input values. The ER luminal region of IRE-1 was used for the above experiments, while the full-length forms of ERGI-2, ERGI-3, and HSP-4 were used for the protein interaction analysis. All interaction ratios were standardized to the Flag-only control group (condition ii) to evaluate the specific effects of ERGI proteins.

### RNA extraction and quantitative RT-PCR

Total RNA was extracted from *C. elegans* using TRIzol reagent (Invitrogen). Three strains (wild type, *ergi-2*, and *ergi-3*) each carrying the P*unc-25*::UNC-9::GFP and P*hsp-4*::mCherry transgenes, were cultured on six 10-cm NGM plates per strain. Animals were collected from the plates with M9 buffer and washed twice more to remove residual bacteria. The worm suspensions were centrifuged at 376 × *g* for 2 min, and the resulting pellets were resuspended in 1 mL of TRIzol reagent and incubated at room temperature for 5 min. After the addition of 200 μL of chloroform (Sinopharm), samples were incubated at room temperature for 2 min and centrifuged at 12,000 × *g* for 15 min at 4°C. The aqueous phase was transferred to a new tube, mixed with 500 μL of isopropanol (Sinopharm), and incubated at room temperature for 10 min. RNA was collected by centrifugation at 12,000 × *g* for 10 min at 4°C, washed with 75% ethanol (Sinopharm) prepared in diethyl pyrocarbonate-treated water, and dissolved in RNase-free water.

Complementary DNA was synthesized from total RNA using SuperScript II Reverse Transcriptase (Invitrogen) according to the manufacturer’s instructions. Quantitative PCR was performed using TransStart Green qPCR SuperMix (TransGen) on a Stratagene Mx3000P real-time PCR system (Agilent). Relative levels of the *unc-9::gfp* transcript were measured using primers specific for the *unc-9::gfp* fusion transcript and normalized to *gapdh* as the internal reference gene.

## Supporting information

S1 Fig *ergi-*2 and *ergi-3* act cell-autonomously in GABAergic neurons to regulate UNC-9::GFP-induced UPR activation. (A) The number of DD/VD neurons, labeled with P*unc-25*::mCherry (red), is unaffected in *ergi-2* and *ergi-3* mutants. Scale bar, 25 μm. (B) Quantification of DD/VD neuron number in (A). (C) P*hsp-4*::mCherry expression (red) in wild-type, *ergi-2*, and *ergi-3* animals and in the corresponding neuron-specific rescue strains. Dashed lines outline the animals, white arrowheads indicate commissures, and asterisks indicate DD/VD neuronal cell bodies. Scale bar, 25 μm. (D) Quantification of P*hsp-4*::mCherry-positive DD/VD neuronal cell bodies in (C). Data are presented as mean ± SEM; n ≥ 30 animals per genotype. Statistical significance was determined by one-way ANOVA followed by Tukey’s multiple-comparisons test. ***p < 0.001; ns, not significant.
(TIF)

S2 Fig The tested COPII-associated components are dispensable for UNC-9::GFP-induced UPR. (A) The number of DD/VD neurons, labeled with P*unc-25*::mCherry (red), is reduced following *sec-12* or *sec-13* RNA interference. (B) Quantification of DD/VD neuron number in (A). (C) P*hsp-4*::mCherry expression (red) is not reduced following *sec-12* or *sec-13* RNA interference. White arrowheads indicate commissures, and asterisks indicate DD/VD neuronal cell bodies. (D) Quantification of P*hsp-4*::mCherry fluorescence intensity in the remaining DD/VD neuronal cell bodies in (C). (E) P*hsp-4*::mCherry expression (red) is unchanged in *ergic-1* and *ile-1* mutants. White arrowheads indicate commissures, and asterisks indicate DD/VD neuronal cell bodies. (F) Quantification of P*hsp-4*::mCherry fluorescence intensity in DD/VD neuronal cell bodies in (E). Scale bars, 25 μm in (A), (C), and (E). Data are presented as mean ± SEM; n ≥ 30 animals per condition. Statistical significance was determined by one-way ANOVA followed by Tukey’s multiple-comparisons test. ***p < 0.001; ns, not significant.
(TIF)

S3 Fig Loss of *ergi-2* or *ergi-3* does not alter *unc-9::gfp* transcript levels. Relative *unc-9::gfp* transcript levels were measured by quantitative RT-PCR in wild-type, *ergi-2*, and *ergi-3* animals carrying the P*unc-25*::UNC-9::GFP transgene. Transcript levels were normalized to *gapdh* and expressed relative to wild type. Data are presented as mean ± SEM; n = 3. Statistical significance was determined by one-way ANOVA followed by Tukey’s multiple-comparisons test. ns, not significant.
(TIF)

S4 Fig Interactions among HSP-4, ERGI-2, ERGI-3, and UNC-9. (A) Full-length and truncated forms of HSP-4::Myc co-immunoprecipitate with Flag::ERGI-2. (B) Human ERGIC2 and ERGIC3 co-immunoprecipitate with human BiP. (C) IRE-1::HA does not co-immunoprecipitate with Flag::ERGI-2 or Flag::ERGI-3.
(TIF)

S5 Fig Overexpression of ERGI-2 or ERGI-3 does not enhance the UPR induced by UNC-6::GFP or VIT-2::GFP overexpression. (A) Quantification of P*hsp-4*::mCherry-positive DD/VD neuronal cell bodies in UNC-6::GFP-overexpressing strains with or without neuronal overexpression of *ergi-2* or *ergi-3*. (B) Quantification of P*hsp-4*::mCherry-positive DD/VD neuronal cell bodies in VIT-2::GFP-overexpressing strains with or without neuronal overexpression of *ergi-2* or *ergi-3*. ns, not significant.
(TIF)

S6 Fig Subcellular localization of UNC-9::GFP in *ergi-2* and *ergi-3* mutants. (A) Colocalization of UNC-9::GFP (green) with markers of autophagosomes (mCherry::LGG-1), early endosomes (mCherry::RAB-5), late endosomes (mCherry::RAB-7), and lysosomes (LAAT-1::mCherry) in DD/VD neurons of wild-type, *ergi-2*, and *ergi-3* animals. Scale bar, 5 μm. (B) Quantification of UNC-9::GFP colocalization with the indicated organelle markers using Pearson’s correlation coefficient. Sample sizes are indicated above the bars. Data are presented as mean ± SEM.
(TIF)

S7 Fig Endogenous neuronal UNC-9 distribution is unaffected by *ergi-2*, *ergi-3*, *ire-1*, or *xbp-1* mutations. (A) Endogenous UNC-9, visualized using UNC-9 split-GFP (UNC-9-spGFP; green, white arrowheads), in DD/VD neurons labeled with P*unc-25*::mCherry (red). UNC-9-spGFP distribution is not detectably altered in *ergi-2*, *ergi-3*, *ire-1*, or *xbp-1* mutants. Scale bar, 25 μm. (B) Quantification of the proportion of DD/VD neuronal cell bodies containing UNC-9-spGFP puncta. Data are presented as mean ± SEM. Statistical significance was determined by one-way ANOVA followed by Tukey’s multiple-comparisons test. ns, not significant.
(TIF)

S1 Table Transgenic strains. (XLSX)

S1 Rawgels Raw images of blots. (PDF)
