# Supplementary material for: ER-to-Golgi transport machinery promotes the excessive cargo-triggered unfolded protein response in C. elegans
Source: PLoS Genet. 2026 Sep 8;22(9):e1012301. doi: 10.1371/journal.pgen.1012301 (PMC13568704; doi:10.1371/journal.pgen.1012301)
Supplement: Supplementary file 1 [file pgen.1012301.s009.pdf]

Fig 6D

All raw images of blots were captured by MiniChemi 610 chemiluminescence imaging system (Sagecreation)

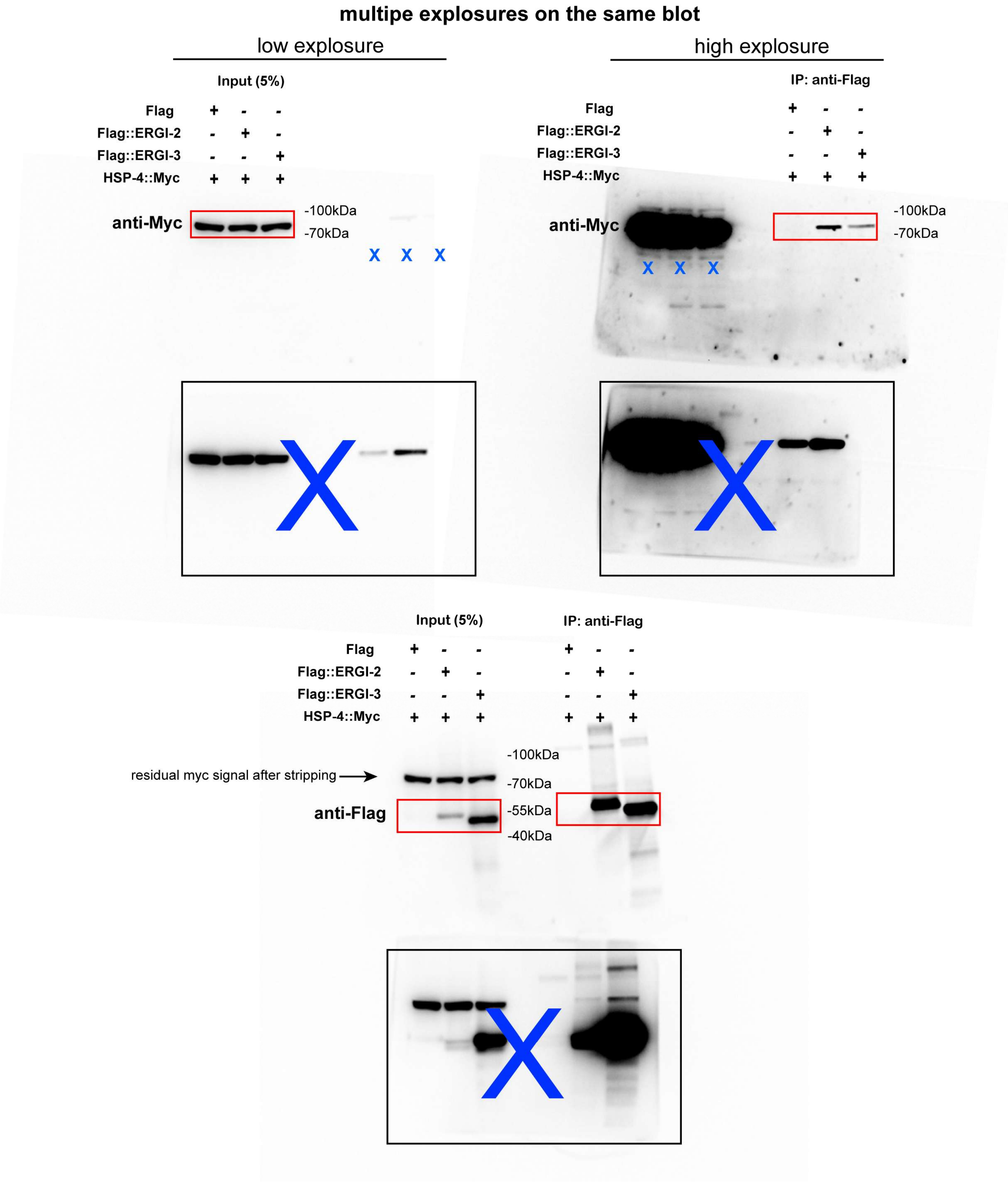

Fig 6 E

multipe explosures on the same blot

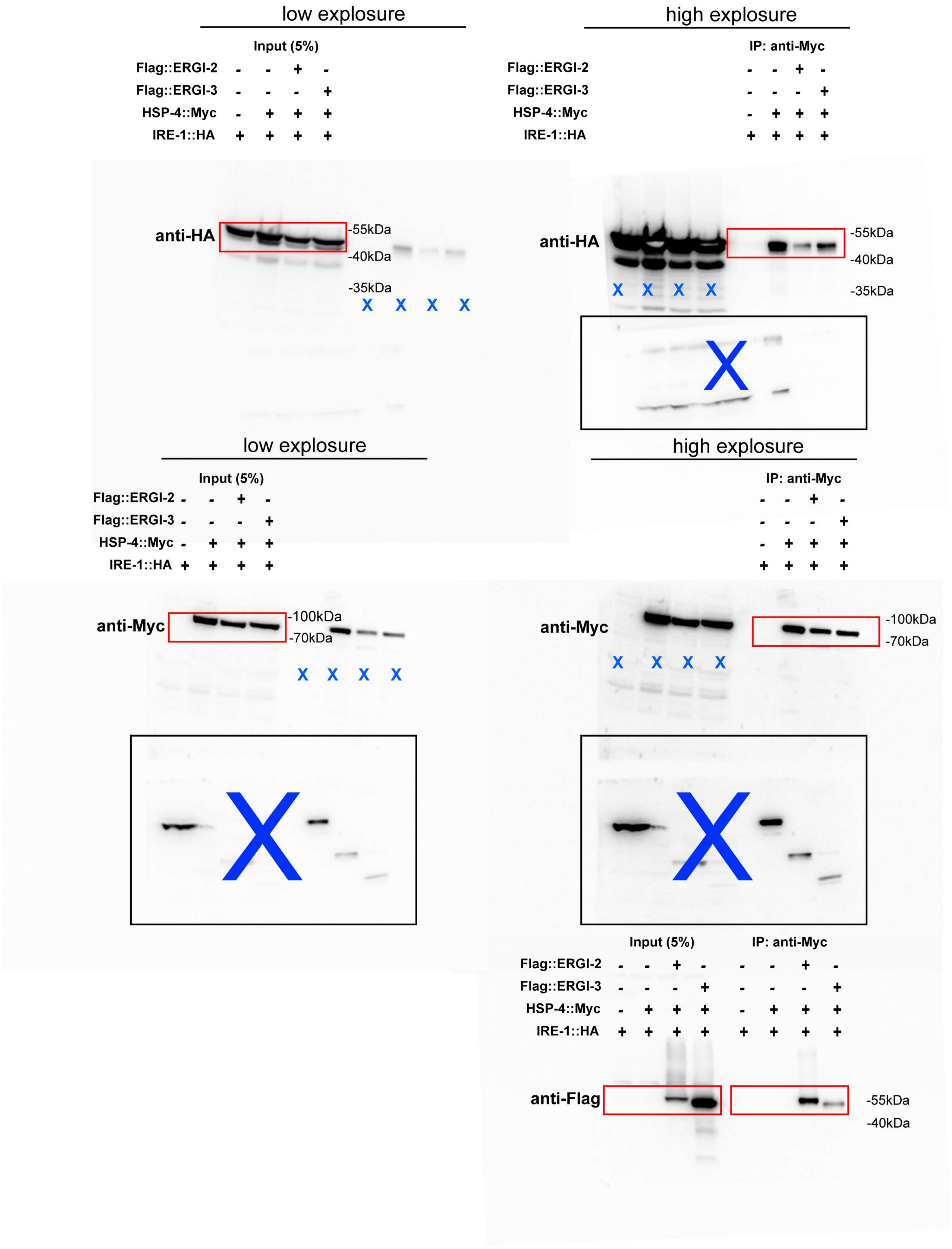

Fig 6 G

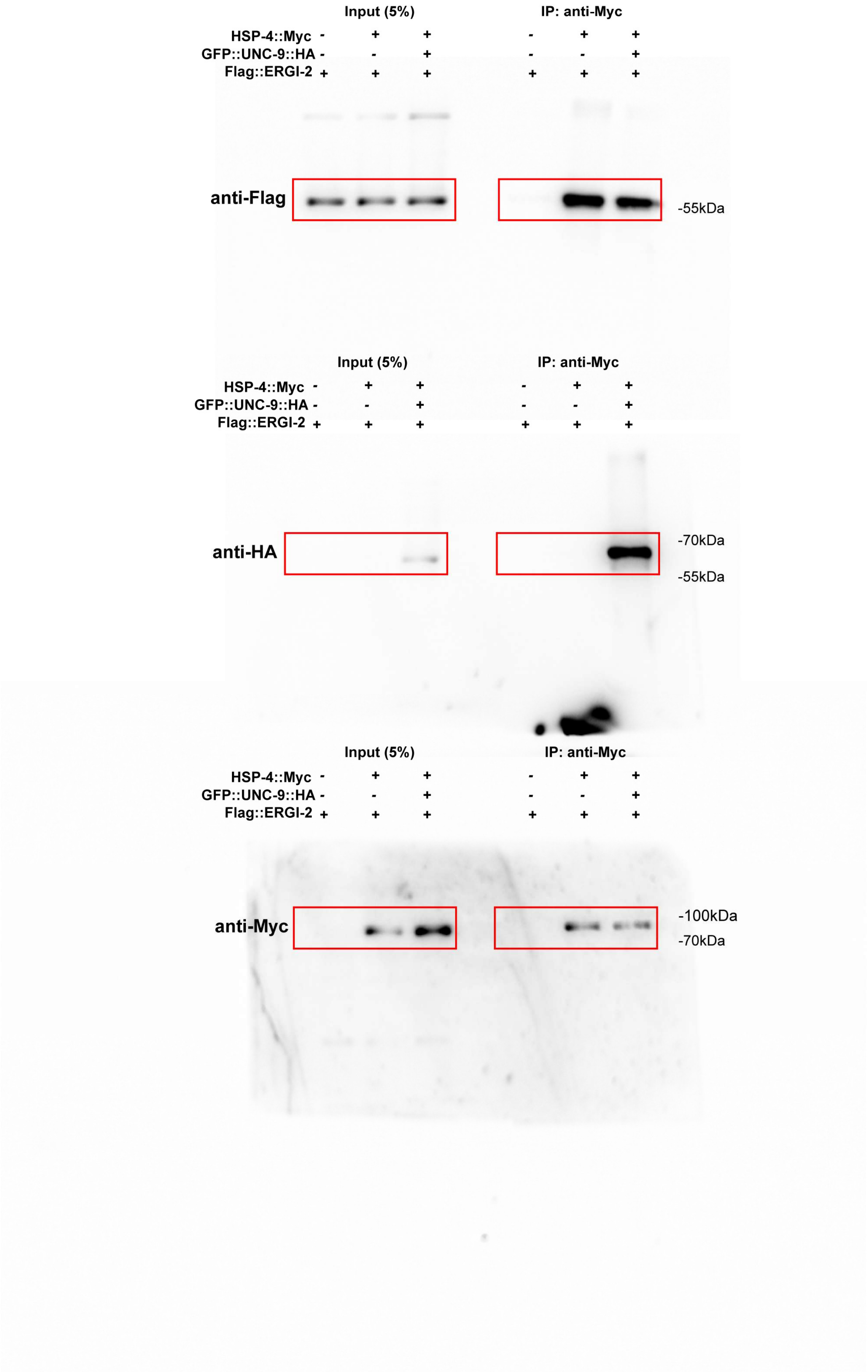

Input (5%)
IP: anti-Myc
HSP-4::Myc - + + - + +
GFP::UNC-9::HA - - + - - +
Flag::ERGI-2 + + + + + +
anti-Flag
-55kDa
Input (5%)
IP: anti-Myc
HSP-4::Myc - + + - + +
GFP::UNC-9::HA - - + - - +
Flag::ERGI-2 + + + + + +
anti-HA
-70kDa
-55kDa
Input (5%)
IP: anti-Myc
HSP-4::Myc - + + - + +
GFP::UNC-9::HA - - + - - +
Flag::ERGI-2 + + + + + +
anti-Myc
-100kDa
-70kDa

Fig 6 H

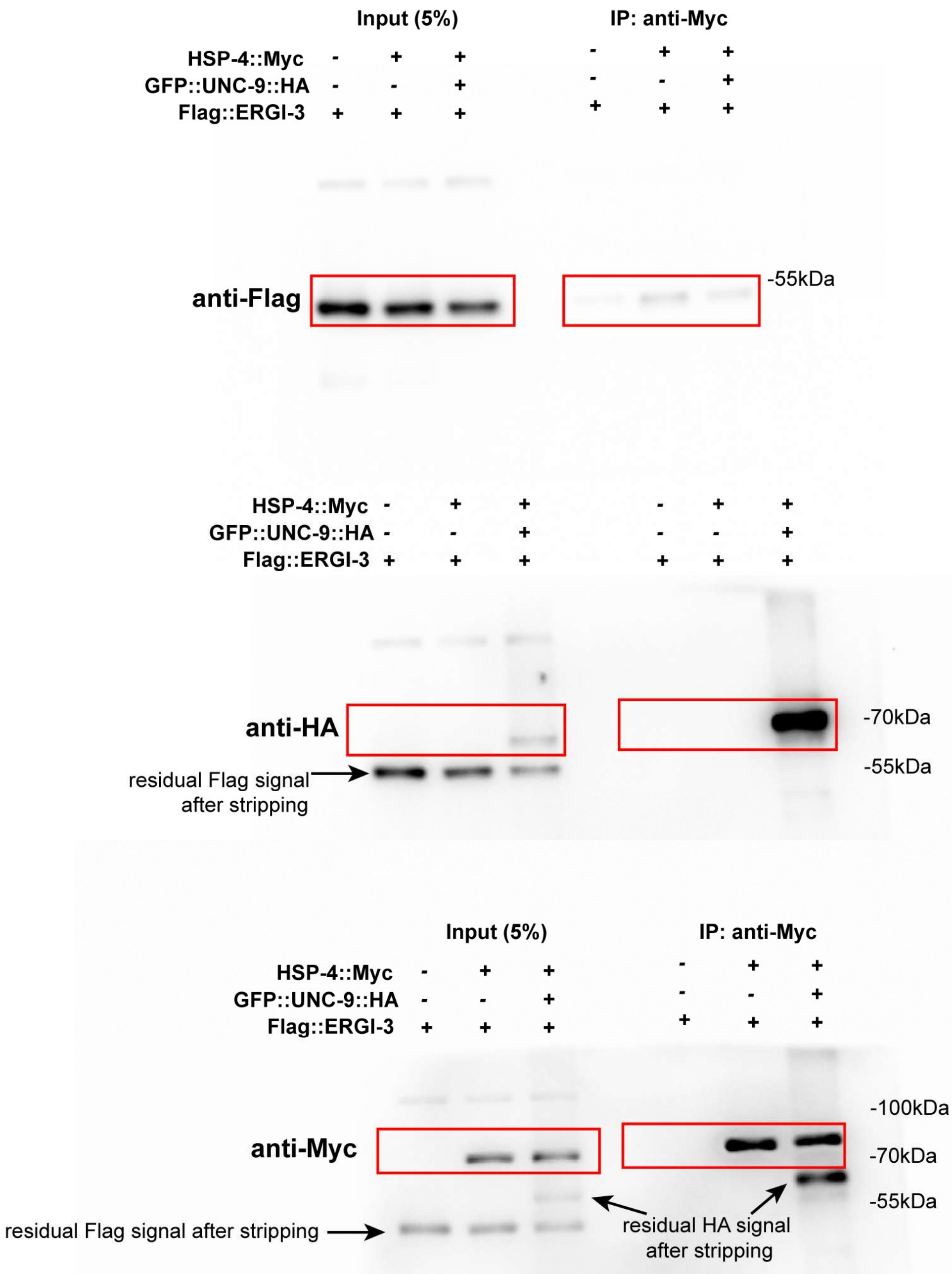

Input (5%)
IP: anti-Myc
HSP-4::Myc - + + - + +
GFP::UNC-9::HA - - + - - +
Flag::ERGI-3 + + + + + +
anti-Flag
-55kDa
HSP-4::Myc - + + - + +
GFP::UNC-9::HA - - + - - +
Flag::ERGI-3 + + + + + +
anti-HA
-70kDa
-55kDa
residual Flag signal after stripping
Input (5%)
IP: anti-Myc
HSP-4::Myc - + + - + +
GFP::UNC-9::HA - - + - - +
Flag::ERGI-3 + + + + + +
-100kDa
anti-Myc
-70kDa
-55kDa
residual Flag signal after stripping
residual HA signal after stripping

S4 Fig A

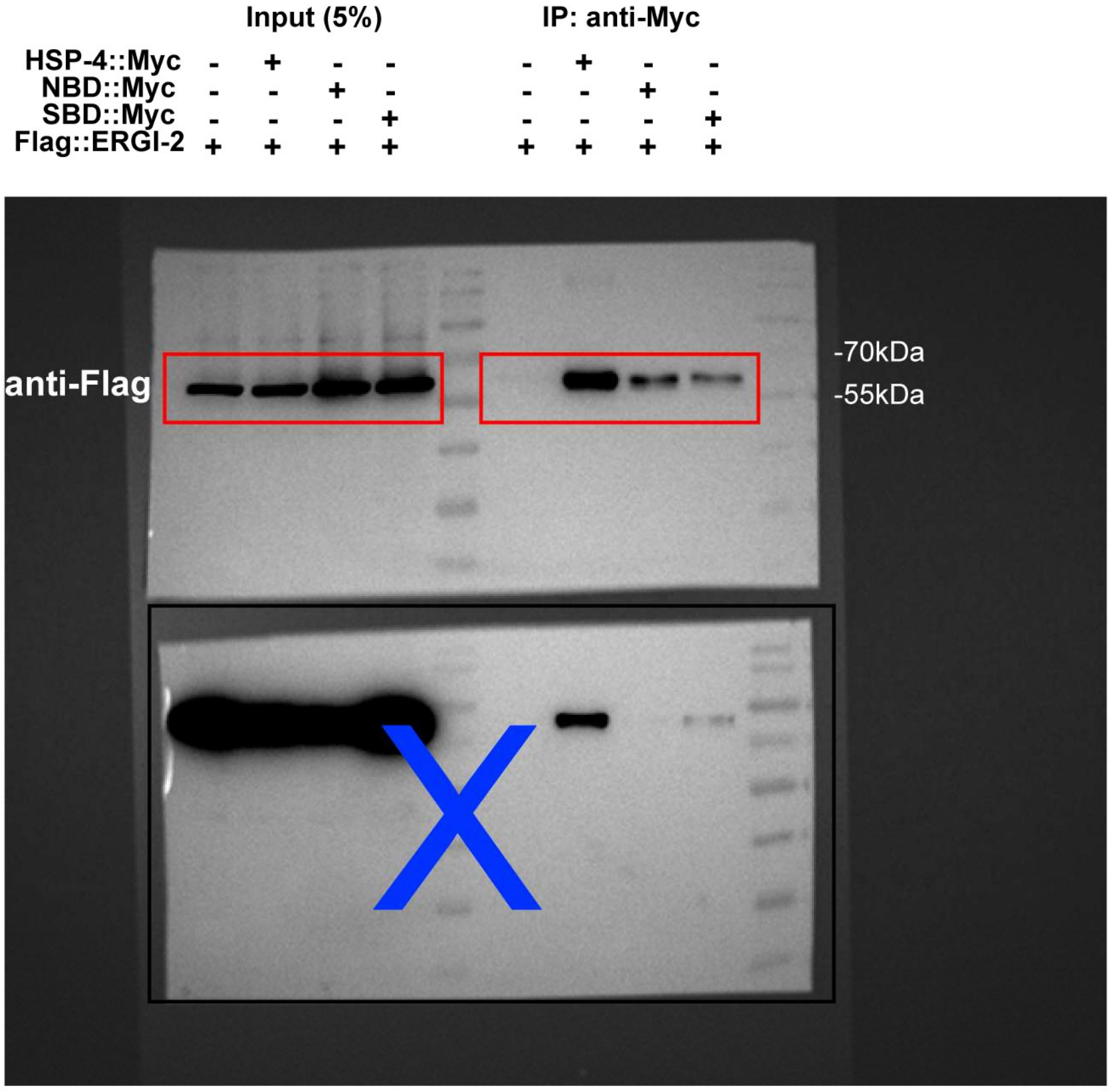

Input (5%)
IP: anti-Myc
HSP-4::Myc - + - - - + - -
NBD::Myc - - + - - - + -
SBD::Myc - - - + - - - +
Flag::ERGI-2 + + + + + + + +
anti-Flag
-70kDa
-55kDa

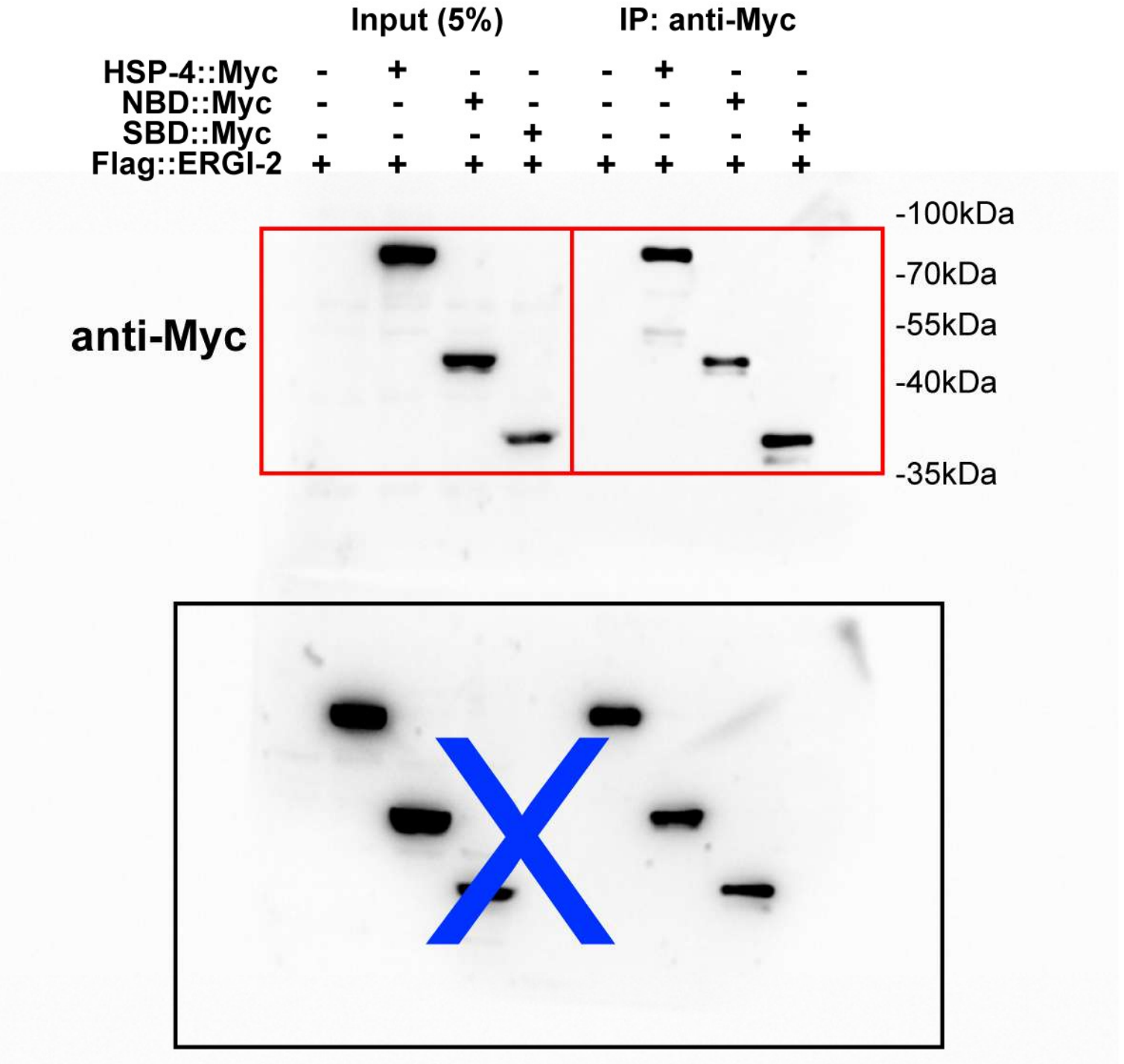

Input (5%)
IP: anti-Myc
HSP-4::Myc - + - - - + - -
NBD::Myc - - + - - - + -
SBD::Myc - - - + - - - +
Flag::ERGI-2 + + + + + + + +
anti-Myc
-100kDa
-70kDa
-55kDa
-40kDa
-35kDa

S4 Fig B

multipe explosures on the same blot

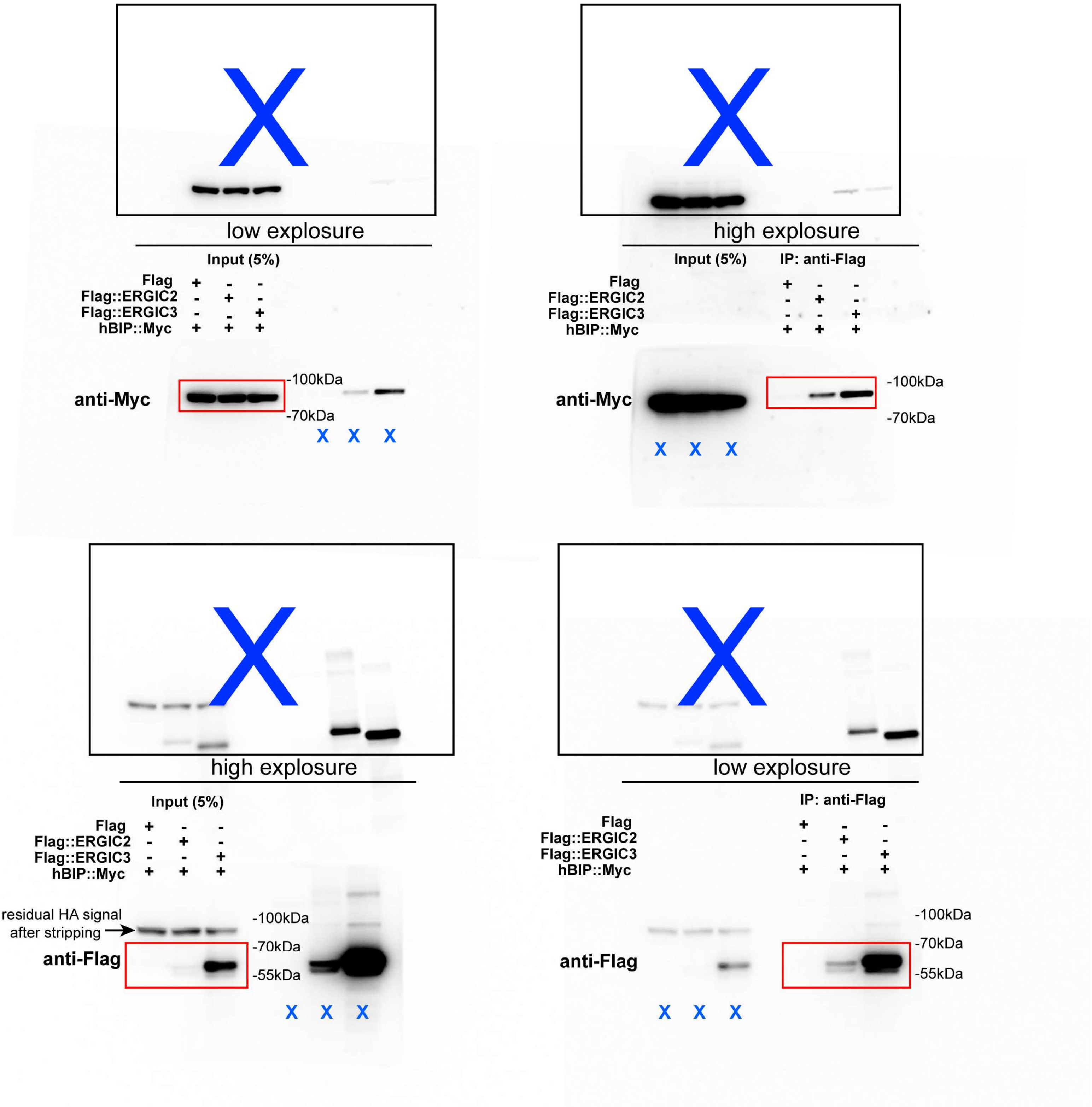

S4 Fig C

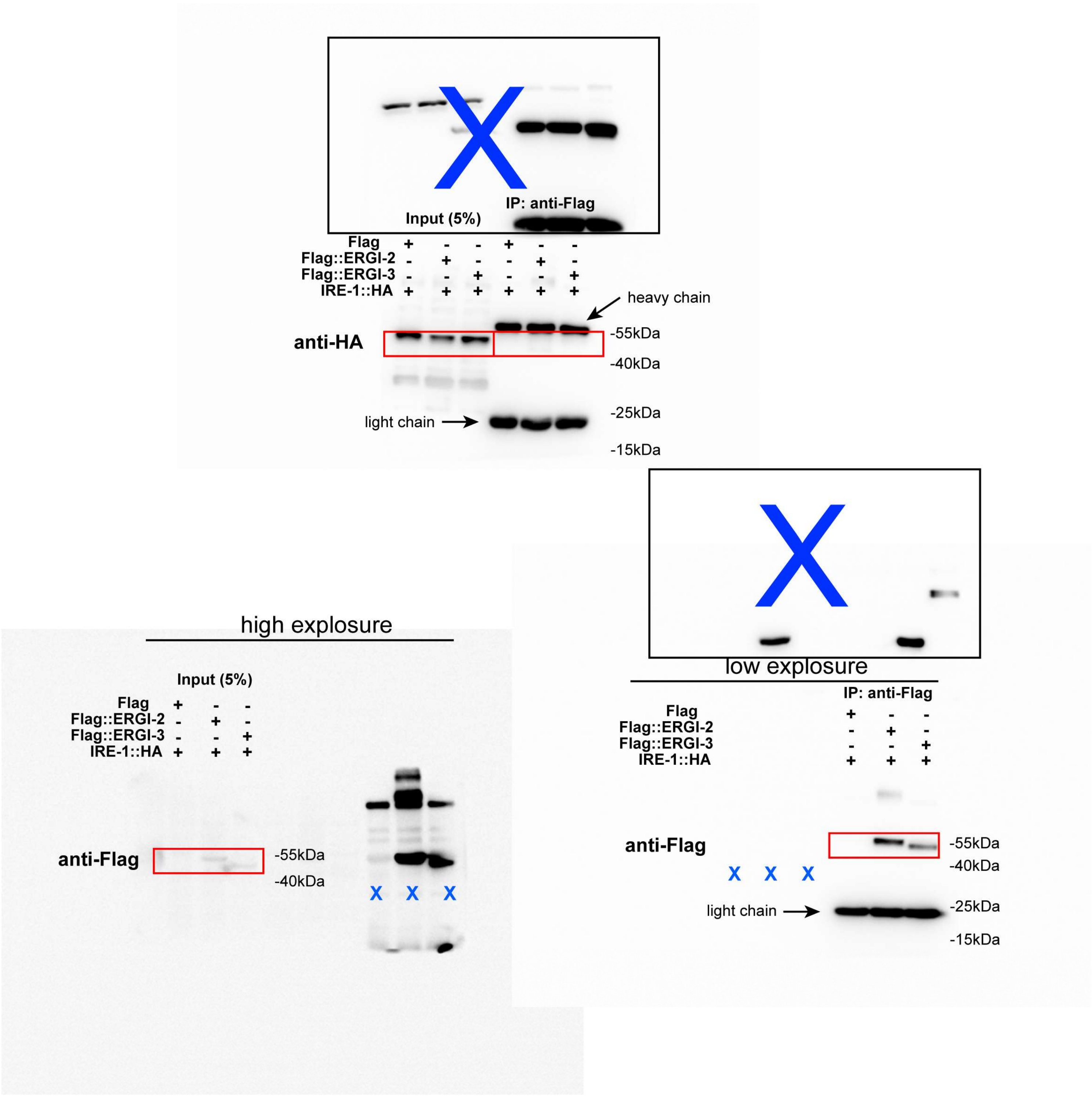
Input (5%)
IP: anti-Flag
Flag + - - + - -
Flag::ERGI-2 - + - - + -
Flag::ERGI-3 - - + - - +
IRE-1::HA + + + + + +
heavy chain
anti-HA
-55kDa
-40kDa
light chain
-25kDa
-15kDa
high explosure
Input (5%)
Flag + - -
Flag::ERGI-2 - + -
Flag::ERGI-3 - - +
IRE-1::HA + + +
anti-Flag
-55kDa
-40kDa
X X X
low explosure
IP: anti-Flag
Flag + - -
Flag::ERGI-2 - + -
Flag::ERGI-3 - - +
IRE-1::HA + + +
anti-Flag
-55kDa
-40kDa
X X X
light chain
-25kDa
-15kDa
