# Supplementary material for: ER-to-Golgi transport machinery promotes the excessive cargo-triggered unfolded protein response in C. elegans
Source: PLoS Genet. 2026 Sep 8;22(9):e1012301. doi: 10.1371/journal.pgen.1012301 (PMC13568704; doi:10.1371/journal.pgen.1012301)
Supplement: Supplementary file 2 [file pgen.1012301.s011.docx]

Dear Editors of *PLoS Genetics*,

We are grateful to you and the reviewers for the thoughtful and constructive comments, which have helped us substantially strengthen the manuscript.

In response to the reviewers’ comments and suggestions, we have performed all the requested experiments and incorporated the resulting data into the revised manuscript.

Among the major additions, we have:

1) Examined whether overexpression of another innexin, UNC-7 induces the UPR and whether these responses depend on ERGI-2 and ERGI-3.

2) Demonstrated that untagged UNC-9 also induces the UPR, excluding the possibility that the response is caused by the GFP tag.

3) Performed neuron-specific rescue experiments to establish the cell-autonomous functions of ERGI-2 and ERGI-3 in regulating UNC-9 localization and UPR activation.

4) Expanded the subcellular localization analysis of UNC-9::GFP using markers for the ER, Golgi, molecular chaperones, early and late endosomes, autophagosomes, and lysosomes.

5) Added experiments and quantitative analyses to further validate autophagy as an independent readout of UNC-9-induced UPR activation.

6) Confirmed that the altered UNC-9::GFP localization in *ergi-2* and *ergi-3* mutants is not caused by changes in *unc-9::gfp* transcript levels.

7) Added detailed criteria for image quantification and provided additional controls and raw imaging data to improve the rigor and reproducibility of the study.

We have incorporated these new results into the main and supplementary figures and revised the Results, Materials and Methods, Introduction, and Discussion accordingly. We have also revised the title and abstract to more accurately describe the study and its experimental system. In addition, the point-by-point response explains how each concern was addressed and indicates where the corresponding changes can be found in the revised manuscript.

We believe that these additions fully address the reviewers’ concerns, provide stronger support for our conclusions, and make the revised manuscript suitable for publication in PLoS Genetics.

Sincerely yours,

Mei Ding

Reviewer #1: Guan, Ding and colleagues explore how C. elegans vesicular trafficking machinery regulates ER stress responses induced by neuronal overexpression of gap junction proteins. Built on a prior study published by the same group of authors a few years ago, they investigated how overexpression of the C. elegans innexin UNC-9 in the GABAergic DD/VD motor neurons elicited ER unfolded protein responses (UPR-ER). They focused on ERGI-2 and ERGI-3, two proteins of the ER-Golgi trafficking pathway. Through a series of genetic, imaging and biochemical studies, they provided evidence pointing to a role of ERGI-2 and ERGI-3 dedicated s pecifically to UPR-ER caused by the expression of UNC-9, but not those by expressing other secreted proteins (netrin, vitellogenin) or by stressors (Tunicamycin, heat stress). Several lines of evidence suggest a model in which ERGI-2 and ERGI-3 recruit HSP-4, the ER stress chaperone, which frees the ER-resident kinase/endonuclease IRE-1 for splicing the xbp-1s messenger RNA. The findings that ER-Golgi trafficking proteins regulate UPR-ER are consistent with recent findings that Golgi trafficking proteins are involved in the regulation of ER stress responses. Most of the experiments were properly carried out, and the quantification of data was rigorous. Together these observations provide interesting insights on the mechanisms of proteostasis in the neurons. Below are issues that need to be addressed to further consolidate the major conclusions of the study. Below are key issues that the authors should address to further substantiate the major conclusions of the paper.

Major comments:

1. The nature of UNC-9 overexpression in the GABAergic neurons needs to be clarified, including the following: (1) Does expression of other gap junction proteins, such as UNC-7, show similar effects? (2) Is induction of neuron-autonomous UPR-ER by UNC-9 specific to GABAergic neurons, or does it also occur in other neuronal types?

Response: We thank the reviewer for these insightful comments. To address these questions, we performed the following additional experiments.

**(1) Does expression of other gap junction proteins, such as UNC-7, produce similar effects?**

To determine whether UPR activation is specific to UNC-9 overexpression, we overexpressed UNC-7 in GABAergic neurons using the unc-25 promoter. Similar to UNC-9 overexpression, UNC-7 overexpression increased transcription of the ER stress reporter hsp-4 (Fig 4E and 4F), indicating that elevated expression of another gap junction protein can also activate the UPR.

We next introduced ergi-2 or ergi-3 mutations into the UNC-7-overexpressing strain. In marked contrast to their suppression of the UNC-9-induced UPR, loss of either ergi-2 or ergi-3 did not reduce hsp-4 induction caused by UNC-7 overexpression (Fig 4E and 4F). Moreover, although ERGI-2 and ERGI-3 are required for the proper localization of UNC-9, neither protein was required for the normal distribution of UNC-7 in body-wall muscle cells or GABAergic neurons (Fig 5J and 5K).

Together, these findings distinguish the general cellular response to innexin overexpression from the selective function of ERGI-2 and ERGI-3. Although overexpression-induced UPR activation is not unique to UNC-9, ERGI-2 and ERGI-3 specifically regulate UNC-9 localization and the UPR response associated with UNC-9 overload. Their dispensability for both UNC-7 localization and UNC-7-induced UPR strongly argues that ERGI-2 and ERGI-3 are neither general components of the UPR machinery nor general mediators of the response to innexin overexpression.

**(2) Is the neuron-autonomous UPR induced by UNC-9 specific to GABAergic neurons, or does it also occur in other neuronal types?**

To determine whether UNC-9 overexpression activates the UPR in other neuronal types, we examined animals expressing UNC-9::GFP in a subset of interneurons under the control of the unc-53 promoter (Guan et al., 2020). In these neurons, UNC-9::GFP displayed a punctate distribution in neuronal cell bodies and processes. As shown in our previous study, loss of ire-1 or xbp-1 caused UNC-9::GFP to become diffusely distributed in the cell-body region, indicating that the IRE-1-XBP-1 pathway contributes to the proper localization or handling of overexpressed UNC-9 in these interneurons. This requirement is consistent with our observations in DD/VD GABAergic neurons in the present study.

Nevertheless, we did not detect a significant increase in P*hsp-4*::mCherry fluorescence in the P*unc-53*-expressing interneurons (Response Figure 1). Thus, under the conditions tested, UNC-9 overexpression in these interneurons did not elicit a detectable hsp-4 transcriptional response. A likely explanation is that P*unc-53* drives a lower level of UNC-9 expression than P*unc-25*, generating a correspondingly weaker ER stress response. At a relatively low UNC-9 load, the requirement for IRE-1 and XBP-1 in UNC-9 protein handling may be revealed without reaching the threshold required for detectable induction of the hsp-4 reporter. By contrast, the higher UNC-9 expression driven by P*unc-25* in GABAergic neurons produces robust transcriptional induction of ER chaperone genes such as hsp-4.

We therefore cannot conclude that UNC-9-induced UPR activation is intrinsically restricted to GABAergic neurons. Instead, our results indicate that the magnitude and detectable output of the response depend on cellular context and UNC-9 protein load, which may be influenced by neuronal identity and promoter strength. The P*unc-25*-driven GABAergic model provides a robust and experimentally tractable system for studying the neuronal response to chronic UNC-9 overload. Using this model, our previous study identified PMK-3 as a regulator of this response (Guan et al., 2020), whereas the present study establishes cell-autonomous and UNC-9-selective roles for ERGI-2 and ERGI-3.

Response Figure 1: Overexpression of UNC-9::GFP under the P*unc‑53* promoter does not induce *hsp‑4* expression.

2. The authors hypothesized that ERGI-2 and ERGI-3 are dedicated to mitigating ER stress triggered by UNC-9 overexpression. Data supporting this model includes biochemical evidence that ERGI-2 and ERGI-3 interact with UNC-9 and HSP-4 by forming protein complexes. One possibility is that ERGI-2 and ERGI-3 activate IRE-1 by removing HSP-4 away from it. However, one can imagine that such mechanisms will not be specific for ER stress induced by UNC-9, as HSP-4-IRE-1 is a core component of UPR-ER for many different stressors, and ERGI-2 and ERGI-3 can bind HSP-4 without UNC-9. How does such a model explain the specificity of ERGI-2/3 to UPR-ER induced by UNC-9, but not other protein stressors? Could ERGI-2/3 overexpression enhance the ability of the neurons to cope with ER stress induced by non-UNC-9 factors, such as Tunicamycin, heat or other secreted or misfolded proteins?

Response: We thank the reviewer for this insightful comment and for the opportunity to clarify our model. We agree that the ability of ERGI-2 and ERGI-3 to bind HSP-4, by itself, cannot explain their apparent specificity for the UPR induced by UNC-9 overexpression. Indeed, our biochemical assays show that ERGI-2 and ERGI-3 can associate with HSP-4 in the absence of UNC-9 (Fig 6D). Our *in vivo* findings, however, suggest that the involvement of ERGI-2 and ERGI-3 in UPR activation is determined primarily by their cargo-specific functions, rather than by a general ability to regulate HSP-4 or IRE-1 (Figs 1, 3-4).

Several observations support this interpretation. First, ERGI-2 and ERGI-3 are required for the proper subcellular localization of UNC-9 in both GABAergic neurons and body-wall muscle cells (Fig 5A) (Guan et al., 2022). Together with our previous finding that ERGI-2 and ERGI-3 interact with UNC-9, these results suggest that UNC-9 is a specific cargo of ERGI-2 and ERGI-3. Second, overexpression of another innexin, UNC-7, in GABAergic neurons also activates the UPR; however, this response is not suppressed by loss of either *ergi-2* or *ergi-3* (Fig 4E and 4F). Consistent with this result, neither ERGI-2 nor ERGI-3 is required for the proper localization of UNC-7 in GABAergic neurons or body-wall muscle cells (Fig 5J and 5K). Thus, ERGI-2 and ERGI-3 are not generally required for UPR activation caused by innexin overexpression. Instead, their involvement in this response correlates with their specific role in UNC-9 localization.

We therefore propose that ERGI-2 and ERGI-3 couple the handling of their specific cargo, UNC-9, to the UPR machinery. In this model, the interaction of ERGI-2 and ERGI-3 with HSP-4 occurs in the context of UNC-9 processing or trafficking and facilitates the cellular response to the burden imposed by excessive UNC-9. This cargo-dependent model could explain why ERGI-2 and ERGI-3 are required for UNC-9-induced UPR but not for the response induced by UNC-7. Moreover, because ERGI-2 and ERGI-3 can associate with both UNC-9 and HSP-4, our findings do not favor a simple model in which ERGI-2 or ERGI-3 activates IRE-1 solely by sequestering HSP-4 away from it. We have therefore revised the manuscript to present this mechanistic possibility more cautiously and to emphasize the cargo-dependent nature of our model.

To test more directly whether ERGI-2 and ERGI-3 generally enhance neuronal UPR activation, we overexpressed either ERGI-2 or ERGI-3 in animals in which the UPR was induced by overexpression of UNC-6::GFP or VIT-2::GFP (S5A and S5B Fig). In neither case did ERGI-2 or ERGI-3 overexpression further enhance *hsp-4* reporter induction. We did not directly test the effects of ERGI-2 or ERGI-3 overexpression under tunicamycin- or heat-induced stress because these treatments caused strong *hsp-4* reporter activation, making any additional enhancement difficult to quantify reliably. Nevertheless, the results obtained with UNC-7, UNC-6, and VIT-2 argue against a general role for ERGI-2 and ERGI-3 in increasing the magnitude or sensitivity of the neuronal UPR.

Collectively, our results support a model in which the contribution of ERGI-2 and ERGI-3 to UPR activation depends on their recognition and handling of specific cargo proteins, particularly UNC-9. Their interaction with HSP-4 may facilitate this cargo-associated response, but HSP-4 binding alone does not appear sufficient to enhance UPR activation induced by unrelated protein stressors. We have clarified and appropriately moderated this model in the revised manuscript.

3. Although unc-9 is expressed in VD and DD, according to the C. elegans single neuron gene expression dataset, the expression level is low (212 (rank: 67) in VD and 181 (rank: 73) in DD). In contrast, they are expressed in several non-neuronal cells, and in one of the authors’ prior papers, ergi-2 and erg-3 were shown to act in the muscles. Therefore, the authors should examine whether ergi-2 and ergi-3 act cell-autonomously in VD/DD neurons to modulate ER Stress response caused by UNC-9 overexpression.

Response: We thank the reviewer for this important suggestion. We agree that, because ergi-2 and ergi-3 function in body-wall muscle cells and unc-9 is expressed in multiple neuronal and non-neuronal cell types, it was necessary to determine whether ERGI-2 and ERGI-3 act cell-autonomously in DD/VD neurons.

To address this question, we expressed wild-type ergi-2 or ergi-3 specifically in GABAergic neurons using the unc-25 promoter in the corresponding mutant backgrounds. GABAergic expression of either ergi-2 or ergi-3 restored the hsp-4 induction caused by UNC-9 overexpression (S1C and S1D Fig). These results demonstrate that ERGI-2 and ERGI-3 could function cell-autonomously in DD/VD neurons to mediate the ER UPR response induced by UNC-9 overexpression.

We also agree with the reviewer that endogenous unc-9 expression is relatively low in DD and VD neurons. However, the UPR response examined here is induced by transgenic UNC-9 overexpression rather than by its endogenous expression. The magnitude of this response is therefore likely determined by the level of transgene-driven UNC-9 expression and the capacity of a given neuronal type to process the resulting protein load. Consistent with this interpretation, we previously examined UNC-9::GFP expressed in a subset of interneurons using the unc-53 promoter. UNC-9::GFP showed a punctate distribution in the cell bodies and processes of these neurons and loss of ire-1 or xbp-1 disrupted this distribution and caused UNC-9::GFP to become diffuse within the cell-body region (Guan et al., 2020), indicating that the IRE-1-XBP-1 pathway contributes to the handling of excessive UNC-9 in these interneurons. However, Punc-53::UNC-9::GFP did not produce a detectable increase in Phsp-4::mCherry fluorescence. This difference may reflect a lower UNC-9 expression level from Punc-53 than from Punc-25, differences in the proteostatic capacity of the targeted neurons, or both.

Thus, our findings do not establish that UNC-9-induced ER UPR activation is intrinsically specific to GABAergic neurons. Rather, DD/VD neurons provide an experimentally accessible system in which sustained UNC-9 overexpression produces a readily detectable UPR response. Using this model, our previous study identified a role for PMK-3 (Guan et al., 2020), and the present work identifies cell-autonomous functions of ERGI-2 and ERGI-3 in the neuronal response to chronic UNC-9 protein load.

4. The sensor for xbp-1 splicing is interesting, but using the disappearance of signals as evidence of a positive event is sometimes confounded by technical issues that lead to lack of signals unrelated to the regulatory mechanisms. Could the authors design an alternative sensor such that baseline mCherry expression is prevented by an out-of-frame design, and mCherry is in the reading frame when the xbp-1 short intron is removed by IRE-1? This will ensure that expression of mCherry is indeed triggered by xbp-1 splicing.

Response: We thank the reviewer for raising this important concern. We agree that using signal disappearance to report a positive regulatory event can, in principle, be confounded by technical factors unrelated to the mechanism under investigation. We therefore generated and tested the alternative “signal-on” XBP-1 splicing reporter proposed by the reviewer.

In this reporter, mCherry is out of frame in the un-spliced transcript and therefore is not expressed under basal conditions. IRE-1-mediated removal of the short xbp-1 intron places mCherry in frame, thereby allowing fluorescence to serve as a direct positive readout of xbp-1 splicing. Based on previously described reporter designs (Calfon et al., Nature, 2002; Shim et al., Molecular Biology of the Cell, 2004), we generated 13 independent transgenic lines carrying this stress-activated reporter.

Unfortunately, the signal-on reporter proved insufficiently sensitive in our experimental system. Eleven of the 13 independent lines showed no detectable fluorescence in animals overexpressing UNC-9. The remaining two lines exhibited red fluorescence in only a small number of neuronal cell bodies, and quantification revealed no significant difference between animals with and without UNC-9 overexpression. Thus, despite testing multiple independent transgenic lines, the signal-on reporter did not provide a sufficiently robust or reproducible readout of UNC-9-induced XBP-1 splicing in GABAergic neurons.

By contrast, the signal-off reporter produced a robust decrease in mCherry fluorescence following UNC-9 overexpression. Crucially, this decrease was abolished in ire-1 mutants, in which mCherry fluorescence was restored (Guan et al., 2020). This genetic dependence on ire-1 provides a stringent control for the specificity of the reporter response. In particular, restoration of the signal by loss of ire-1 is not readily explained by transgene silencing, imaging variability, or nonspecific fluorescence loss, but is consistent with IRE-1-dependent splicing of the reporter transcript.

Therefore, although we fully implemented and extensively tested the alternative signal-on design suggested by the reviewer, it was not sufficiently sensitive for this experimental setting. We have retained the signal-off reporter because it provides a robust, reproducible, and genetically validated readout of IRE-1-dependent xbp-1 splicing *in vivo*.

5. The imaging study of excessive UNC-9 in subcellular compartments of different genotypes needs further substantiation (Figure 5 and Figure S2). First, please provide images of UNC-9::GFP with markers for ER (CYTB-5::mCherry), chaperones (RFP::HSP-4), early endosomes (mCherry::RAB-5), late endosomes (mCherry::RAB-7), autophagosomes (mCherry::LGG-1) and lysosomes (LAAT-1::mCherry) in the wild type. Second, in both ergi-2 and ergi-3 mutants, UNC-9::GFP in the neuronal cell bodies appears as two big (perinuclear) blobs, whereas CYTB-5::mCherry signal is diffuse in the cytosol (in the extensive ER network?). Therefore, it is possible that UNC-9::GFP in these mutants is aggregated in another membrane organelle that is close to the cell nucleus and intimately associated with ER, such as Golgi. A Golgi marker should be used to further clarify the nature of these aggregates.

Response: We thank the reviewer for these constructive suggestions. We have now expanded the imaging analysis in two ways. First, we included images of UNC-9::GFP together with markers for the ER (CYTB-5::mCherry), the ER chaperone HSP-4 (RFP::HSP-4), early endosomes (mCherry::RAB-5), late endosomes (mCherry::RAB-7), autophagosomes (mCherry::LGG-1), and lysosomes (LAAT-1::mCherry) in wild-type animals. These data are now presented in Figs. 5F, 5G, and S6A. Second, as suggested by the reviewer, we examined whether the large perinuclear UNC-9::GFP accumulations in ergi-2 and ergi-3 mutants are associated with the Golgi.

In wild-type DD/VD neurons, UNC-9::GFP was distributed as small puncta within neuronal cell bodies and along neuronal processes. By contrast, in ergi-2 and ergi-3 mutants, UNC-9::GFP frequently accumulated in two large perinuclear structures within the neuronal cell body. Quantitative colocalization analysis demonstrated that the association of UNC-9::GFP with the ER marker CYTB-5::mCherry was increased in both mutants (Fig 5F and 5I), supporting the interpretation that loss of ERGI-2 or ERGI-3 causes excessive UNC-9 to accumulate in the ER.

We further examined the relationship between UNC-9::GFP and the ER chaperone HSP-4. Interestingly, expression of RFP::HSP-4 changed the morphology of the large UNC-9::GFP accumulations, dispersing them into smaller puncta. These smaller UNC-9::GFP puncta showed substantial colocalization with RFP::HSP-4 in both ergi-2 and ergi-3 mutants. This observation is particularly informative because the large size and high fluorescence intensity of the original UNC-9::GFP accumulations limited the spatial resolution of their subcellular localization. Their redistribution into smaller puncta in the presence of RFP::HSP-4 allowed the close association between UNC-9 and this ER-resident chaperone to be resolved more clearly. The ability of HSP-4 expression to alter these accumulations is also consistent with their representing improperly handled UNC-9 within an ER-associated compartment.

To directly address the reviewer’s alternative explanation, we introduced the Golgi marker MANS::mCherry. Colocalization between UNC-9::GFP and MANS::mCherry was not significantly increased in either ergi-2 or ergi-3 mutants (Fig 5H and 5I). Thus, although the UNC-9::GFP accumulations are perinuclear, our data do not support the possibility that they primarily represent Golgi-associated aggregates. We likewise found no increased association with the examined endosomal, autophagosomal, or lysosomal markers.

This conclusion is further supported by our analysis in body-wall muscle cells (Guan et al., 2022). Because these cells are substantially larger than GABAergic neuronal cell bodies, the subcellular distribution of accumulated UNC-9::GFP can be resolved more readily. In both ergi-2 and ergi-3 mutants, UNC-9::GFP showed increased retention in the ER rather than accumulation in the other organelles examined, consistent with our findings in GABAergic neurons.

Collectively, several independent observations, including enhanced colocalization with CYTB-5::mCherry, substantial co-distribution with RFP::HSP-4, the HSP-4-dependent dispersal of the large accumulations, the absence of increased Golgi colocalization, and the concordant results obtained in muscle cells, strongly support the conclusion that excessive UNC-9 is predominantly retained in ER-associated structures in ergi-2 and ergi-3 mutants. We have added these data and clarified this conclusion in the revised manuscript.

6. Does ER stress caused by UNC-9 overload impair neuronal health or functions? This important question is surprisingly missed in the current and prior publications.

Response: We thank the reviewer for raising this important question and appreciate the opportunity to clarify our previous findings. In our earlier study (Guan et al., 2020), we examined the morphology and survival of DD/VD neurons following UNC-9 overexpression. UNC-9 overload alone did not cause obvious alterations in neuronal morphology or neuronal loss. However, when either ire-1 or xbp-1 was genetically disrupted, UNC-9 overexpression caused significant DD/VD neuronal loss that became progressively more severe with age.

These findings demonstrate that UNC-9 overload imposes a chronic proteostatic challenge but is not overtly neurotoxic when the ER UPR remains functional. Instead, activation of the IRE-1-XBP-1 pathway provides an essential protective response that preserves neuronal integrity and survival under this condition. Thus, the physiological consequence of UNC-9-induced ER stress becomes evident when the adaptive capacity of the UPR is compromised.

7. The authenticity of mCherry::LGG-1 puncta as autophagosomes needs to be further confirmed. Mutating the terminal glycine (G116) should abolish LGG-1 clustering on autophagosomes and therefore diminish puncta formation. This needs to be tested. In addition, the criterion of mCherry::LGG-1 quantification needs to be clearly defined (diameter, for example).

Response: We thank the reviewer for these insightful suggestions. We have addressed both points as described below.

**(1) Validation of mCherry::LGG-1 puncta as autophagosome structures**

To determine whether the observed mCherry::LGG-1 puncta represent authentic autophagosome structures rather than nonspecific aggregates of the fluorescent reporter, we generated the lipidation-deficient mCherry::LGG-1(G116A) reporter. Mutation of the terminal glycine prevents the membrane conjugation of LGG-1 and therefore provides an established negative control for autophagosome membrane association (Tanida et al., *Methods In Molecular Biology* 2008; Zhou et al., *Cell* 2019; Leboutet et al., *Elife* 2023).

UNC-9::GFP overexpression induced a significant increase in puncta formed by wild-type mCherry::LGG-1. By contrast, mCherry::LGG-1(G116A) remained predominantly diffuse and did not form puncta under the same conditions. Moreover, introduction of ergi-2 or ergi-3 mutations did not alter the diffuse distribution of mCherry::LGG-1(G116A) (Fig 2A-2D). These results demonstrate that the puncta observed with wild-type mCherry::LGG-1 depend on the terminal glycine required for LGG-1 lipidation and membrane association, rather than reflecting nonspecific reporter aggregation.

Together with our finding that UNC-9-induced wild-type mCherry::LGG-1 puncta are reduced in ergi-2 and ergi-3 mutants, these data support the conclusion that excessive UNC-9 promotes ERGI-2/3-dependent autophagosome formation.

**(2) Definition of the quantification criteria**

We have added explicit quantification criteria to the revised Materials and Methods section. An mCherry::LGG-1-positive punctum was defined as a spatially distinct, approximately round fluorescent structure with a diameter of at least 0.2 μm and a mean grayscale intensity greater than 50 on an 8-bit scale after subtraction of the local background.

Puncta were quantified within DD/VD neuronal cell bodies in young adult animals. DD/VD cell bodies were identified by their characteristic positions along the ventral nerve cord and were defined as approximately oval regions with an area of 6-20 μm². All images used for comparisons were acquired using identical settings and analyzed using the same thresholds and size criteria.

These validation experiments and quantitative definitions have now been included in Fig 2 and in the revised Results and Materials and Methods sections.

General:

1. The authors should mention C. elegans in the title, Abstract and Introduction, and make it clear that the findings described in this paper were made using this nematode.

Response: We thank the reviewer for this important suggestion. To identify the experimental organism clearly from the outset, we have revised the title to explicitly include C. elegans. The revised title is: “ER-to-Golgi transport machinery promotes the excessive cargo-triggered unfolded protein response in C. elegans.”

We have also revised the Abstract and Introduction to state clearly that the findings reported in this study were obtained using the C. elegans model. These changes ensure that the experimental context and scope of our conclusions are clear to readers throughout the revised manuscript.

2. The Introduction contains too little information and does not provide an accurate and suitable context for the readers to understand why the study fills the gaps in our understanding of ER stress responses. Please substantiate this section.

Response: We sincerely thank the reviewer for this constructive critique. In response, we have substantially expanded and reorganized the Introduction to provide a clearer context for the study.

The revised Introduction now describes: (1) the secretory and proteostatic demands faced by cells *in vivo*; (2) the central role of the IRE-1-XBP-1 pathway and BiP/HSP-4 in maintaining ER proteostasis and (3) the relationship between ER-to-Golgi transport and UPR activation. In particular, we emphasize an important unresolved question: whether early secretory-pathway components simply prevent ER stress by promoting cargo export or actively couple the processing of specific cargoes to UPR signaling.

We have also described our previous findings more clearly and distinguished them from the advances of the present study. Our earlier work showed that UNC-9 overexpression activates the neuronal UPR (Guan et al., 2020) and that ERGI-2 and ERGI-3 regulate UNC-9 trafficking in muscle cells (Guan et al., 2022). The current study connects these findings by demonstrating that ERGI-2 and ERGI-3 link UNC-9 processing to activation of the IRE-1-XBP-1 pathway.

These revisions establish the knowledge gap, the rationale for our experimental model, and the conceptual advance of our study: early secretory-pathway components can actively coordinate cargo-specific trafficking with ER stress signaling.

3. Were Figures 3A and 3C processed for contrast enhancement? Some of the images look oversaturated for pixel values.

Response: We thank the reviewer for raising this concern. The images presented in Figures 3A and 3C were not subjected to contrast enhancement or other selective image processing. All images were acquired using the same standardized imaging settings, which were maintained across the experimental groups to permit direct comparison.

For transparency, we provide the original, unmodified images below for the reviewer’s evaluation. These images confirm that the fluorescence intensities shown in Figures 3A and 3C reflect the original acquisition data rather than post-acquisition contrast enhancement.

4. In the Materials and Methods section, please define how cell bodies with hsp-4::mCherry expression or UNC-9 puncta were defined. Some quantitative criteria of calling UNC-9 puncta, positive mCherry expression or UNC-9 aggregates need to be

provided.

Response: We thank the reviewer for the opportunity to clarify our image-analysis and quantification criteria. We have now explicitly defined the criteria used to identify Phsp-4::mCherry-positive cell bodies, UNC-9::GFP puncta, and UNC-9::GFP aggregates in the Materials and Methods section of the revised manuscript.

DD/VD motor-neuron cell bodies were identified based on their characteristic positions along the ventral nerve cord. A DD/VD cell body was classified as Phsp-4::mCherry-positive when it satisfied all of the following criteria: (1) an approximately oval fluorescent region located along the ventral nerve cord; (2) an area of 6-20 μm², consistent with the size of a DD/VD neuronal cell body; and (3) a mean grayscale intensity greater than 50 on an 8-bit scale after subtraction of the local background.

An individual UNC-9::GFP punctum was defined as a round particle with an area greater than 0.02 μm² and a mean grayscale intensity greater than 40. The following parameters were quantified: (1) puncta intensity, defined as the mean fluorescence intensity of each UNC-9::GFP punctum; (2) puncta number, determined by counting UNC-9::GFP puncta along the neurite segment between the DD5 and VD9 cell bodies, or between the DD4 and DD5 cell bodies at the L1 stage; and (3) puncta size, defined as the area of each UNC-9::GFP punctum. Puncta with an area greater than 1 μm² were classified as aggregates. For each neuronal cell body, the areas of all UNC-9::GFP puncta were summed and normalized to the total cell-body area to calculate the percentage of the soma occupied by UNC-9::GFP puncta. Mean UNC-9::GFP fluorescence intensity was also measured within defined regions of interest in the cell body (elliptical region, 12 μm²) and neurite (rectangular region, 30 × 0.35 μm). The cell body-to-neurite fluorescence-intensity ratio was then calculated.

All images used for comparisons among genotypes were acquired using identical imaging settings and analyzed using the same thresholds and size criteria. These definitions and analysis procedures have now been added to the revised Materials and Methods section to improve the transparency and reproducibility of the quantification.

Reviewer #2: In this manuscript, the authors investigate the role of COPII-associated proteins ERGIC2 and ERGIC3 in gap junction overexpression–mediated IRE1–XBP1 activation. Overall, this is an interesting study that suggests a potentially novel function of COPII-associated proteins beyond cargo trafficking. The proposed signaling pathway appears to be activated by UNC-9::GFP overexpression; however, several key control experiments are missing. In addition, the biological relevance of this pathway under physiological conditions remains unclear.

1. Use of UNC-9::GFP

UNC-9::GFP may not fully reflect the behavior of native gap junction proteins. Previous work (e.g., Chen et al., 2007) showed that UNC-9::GFP can form constitutively open gap junction channels, while other studies suggest that its overexpression can disrupt normal gap junction function and mimic unc-9 loss-of-function phenotypes. Therefore, it is important to reevaluate these observations using untagged UNC-9 to ensure that the reported phenotypes and signaling are not artifacts caused by an abnormal fusion protein. Additionally, UNC-9 has been reported to possess channel-independent functions (Mizumoto, 2022). As the manuscript suggests that gap junction activity is responsible for activating the pathway, it is important to determine whether the channel function of UNC-9 is specifically required for the observed phenotypes.

Response: We appreciate the reviewer’s thoughtful comments and the opportunity to clarify the rationale for using UNC-9::GFP and the nature of the response investigated in this study.

First, we emphasize that our manuscript does not propose that gap-junction channel activity activates the ER UPR. Our study addresses the biosynthesis, ER processing, and early secretory trafficking of UNC-9-not its activity as a channel at the plasma membrane. Accordingly, whether UNC-9::GFP forms constitutively open channels or affects gap-junction function does not alter the central mechanistic question examined here: how neurons respond to an increased UNC-9 protein-processing burden within the early secretory pathway.

Nevertheless, we agree that it is important to establish that the observed response is not unique to the GFP-tagged protein. We therefore expressed untagged UNC-9 in DD/VD neurons. Untagged UNC-9 induced hsp-4 expression, and this response was markedly reduced in ergi-2 and modestly reduced in ergi-3 mutants (Fig 4G-4H). These new results demonstrate that UNC-9 itself is sufficient to activate an ERGI-2/3-dependent UPR response. Thus, the principal phenotype and the requirement for ERGI-2 and ERGI-3 are not artifacts caused by the GFP fusion.

We noticed that UNC-9::GFP produced a stronger UPR response than untagged UNC-9. We consider this a quantitative rather than a qualitative difference. The GFP tag may increase UNC-9 abundance or stability, or it may increase the demand placed on its folding, assembly, or trafficking. Importantly, UNC-9::GFP reaches its characteristic punctate locations at cell-cell junctions in both neurons and muscle cells, demonstrating that the fusion protein remains competent for secretory-pathway transport and membrane delivery. UNC-9::GFP therefore provides a sensitive experimental model of increased UNC-9 processing demand, whereas the untagged UNC-9 experiments independently establish the biological basis of the response.

Our results also strongly associate this response with UNC-9 handling in the early secretory pathway. ERGI-2 and ERGI-3 regulate the intracellular trafficking of UNC-9, interact with UNC-9 and the ER chaperone HSP-4, and are required for full UPR activation in response to an increased UNC-9 load. In their absence, excessive UNC-9 accumulates in ER-associated structures, and the UPR response is attenuated. These findings place ERGI-2 and ERGI-3 mechanistically at the interface between UNC-9 cargo handling and ER stress signaling.

By contrast, gap-junction channel activity may occur only after UNC-9 has exited the ER, reached the plasma membrane, and assembled into intercellular channels. The channel-dependent and channel-independent functions described for UNC-9 therefore concern a later stage of UNC-9 biology than the ER processing and cargo-trafficking events investigated here. Because our manuscript neither attributes UPR activation to gap-junction activity nor uses channel function to support its conclusions, distinguishing between channel-dependent and channel-independent plasma-membrane functions is not required to establish our central model.

Together, the new data with untagged UNC-9 provide direct evidence that an elevated UNC-9 protein load activates an ERGI-2/3-dependent UPR response independently of GFP tagging. We have revised the manuscript to include these results and to state more explicitly that UNC-9::GFP is used as a sensitized model of UNC-9 processing demand, rather than as a tool for examining gap-junction channel activity.

2. Specificity to UNC-9 versus other innexins

In addition to unc-9, multiple innexins are expressed in neurons. It is unclear whether the observed phenotype is specific to UNC-9 or represents a more general property of innexins. This could be addressed by overexpressing other neuronal innexins, such as unc-7 or inx-1, and assessing whether they similarly activate the IRE1–XBP1 pathway.

Response: We thank the reviewer for this excellent suggestion. To determine whether UPR activation is specific to UNC-9 or represents a broader response to increased innexin expression, we examined another well-characterized neuronal innexin, UNC-7, which is expressed in both neurons and muscle cells in *C. elegans*.

Overexpression of UNC-7::GFP in GABAergic neurons significantly increased *hsp-4* transcription, demonstrating that UPR activation is not unique to UNC-9 and can also be induced by an excessive load of another innexin. However, unlike the response induced by UNC-9, UNC-7-induced *hsp-4* expression was not suppressed by loss of *ergi-2* or *ergi-3* (Fig 4E and 4F). Thus, although innexin overexpression can more generally activate the UPR, the requirement for ERGI-2 and ERGI-3 is selective for the response to UNC-9.

We next examined whether this differential requirement correlates with the cargo-trafficking functions of ERGI-2 and ERGI-3. In wild-type muscle cells, UNC-7::GFP displayed a punctate distribution at cell-cell junctions, and this pattern was unaffected in *ergi-2* or *ergi-3* mutants (Fig 5J). Similarly, in DD/VD neurons, UNC-7::GFP formed puncta within neuronal cell bodies and along neuronal processes, and its distribution remained unchanged in the absence of ERGI-2 or ERGI-3 (Fig 5K). By contrast, as we previously demonstrated, ERGI-2 and ERGI-3 are required for the proper localization of UNC-9 in both muscle cells and GABAergic neurons.

Together, these findings distinguish two aspects of the response. First, UPR activation is not exclusive to UNC-9, because UNC-7 overexpression also induces *hsp-4*. Second, ERGI-2/3 dependence is cargo-selective: these proteins regulate both the localization of UNC-9 and the UPR to UNC-9 overload, but they are dispensable for UNC-7 localization and UNC-7-induced UPR. These results support our model that ERGI-2 and ERGI-3 couple the trafficking or processing of their specific cargo, UNC-9, to UPR signaling rather than serving as general regulators of the response to all innexins.

We have included these new data in Figs 4-5 and revised the Results and Discussion to clarify the distinction between the broader capacity of innexin overload to activate the ER UPR and the selective involvement of ERGI-2 and ERGI-3 in the response to UNC-9.

3. Cell-type specificity

It is unclear whether this phenotype is neuron-specific or reflects a broader cellular mechanism. Since unc-9 is expressed in both neurons and muscles, it would be informative to test whether overexpression of UNC-9 in muscle cells also activates IRE1–XBP1 signaling.

Response: We thank the reviewer for this important suggestion. UNC-9 is expressed in both neurons and muscles, and we agree that testing whether the observed UPR activation is neuron-specific or reflects a more general cellular mechanism is essential.

In our previous work, we found that expression of UNC-9 in muscle cells (driven by the P*myo-3* promoter) did not activate the UPR (Guan et al., 2022), suggesting that the effect is not universal across all cell types. To further examine whether UNC-9-induced UPR activation is specific to GABAergic neurons or can occur in other neuronal subtypes, we expressed UNC-9::GFP in a set of interneurons using the P*unc-53* promoter. In these interneurons, UNC-9::GFP exhibited a punctate distribution in both cell bodies and neuronal processes, similar to that observed in DD/VD GABAergic neurons. Moreover, consistent with our findings in GABAergic neurons, loss of *ire-1* or *xbp-1* caused UNC-9::GFP to become diffusely distributed within the cell-body region (Guan et al., 2020), indicating that the IRE-1-XBP-1 pathway is also required for proper localization or handling of overexpressed UNC-9 in these interneurons.

However, we did not detect a significant increase in P*hsp-4*::mCherry fluorescence in the P*unc-53*-expressing interneurons (Response Figure 1). Thus, under our experimental conditions, UNC-9 overexpression in these cells did not induce a detectable *hsp-4* transcriptional response. One possible explanation is that the P*unc-53* promoter drives a lower level of UNC-9 expression compared to P*unc-25*, resulting in a correspondingly weaker UPR output. At relatively low overexpression levels, the requirement for IRE-1 and XBP-1 in UNC-9 protein localization may still be observable without producing sufficient ER stress to induce measurable *hsp-4* reporter expression. At higher expression levels, such as those driven by P*unc-25* in GABAergic neurons, transcriptional induction of ER chaperone genes like *hsp-4* becomes readily detectable.

Interestingly, endogenous *unc-9* expression in DD/VD neurons is rather low. We speculate that this low basal expression may render these neurons particularly sensitive to UNC-9 overexpression, making it easier to trigger a detectable UPR in this cell type compared to others. This property gives us a unique advantage to explore the mechanisms underlying chronic stress responses *in vivo*, which may mimic pathological conditions relevant to human neuronal disorders. Using this UNC-9 overexpression model, we have not only revealed the role of PMK-3 (Guan et al., 2020) but also identified ERGI-2 and ERGI-3 as key players in UPR regulation-findings that might have been missed in other UPR models.

We have clarified these points in the revised manuscript to better address the cell-type specificity of the observed phenotype.

Response Figure 1: Overexpression of UNC-9::GFP under the P*unc‑53* promoter does not induce *hsp‑4* expression.

4. Physiological relevance

The proposed signaling pathway appears to be triggered by UNC-9::GFP overexpression, and the requirement for ERGIC2/ERGIC3 also seems to depend specifically on this condition. As such, the physiological relevance of this pathway remains unclear. While it may be technically challenging to address this directly with current tools, the authors should more thoroughly discuss the potential biological significance and contexts in which this signaling mechanism might operate.

Response: We thank the reviewer for raising this important point and for the opportunity to clarify the biological significance of our findings. Although UNC-9 overexpression provides the experimental challenge in our study, this system models a physiologically relevant problem faced by neurons: maintaining ER homeostasis while synthesizing, folding, assembling, and trafficking abundant multispanning membrane proteins. Our findings identify ERGI-2 and ERGI-3 as cargo-selective factors that couple an increased UNC-9 processing load to activation of the UPR.

First, we excluded the possibility that the observed response was caused by the GFP tag. Overexpression of untagged UNC-9 in DD/VD neurons induced hsp-4 reporter expression, and this induction was attenuated by ergi-2 or ergi-3 mutation (Fig 4G and 4H). Thus, UPR activation is driven by an increased UNC-9 protein load rather than by a nonspecific effect of GFP.

We next asked whether UPR is a general consequence of increased innexin expression and, importantly, whether the requirement for ERGI-2 and ERGI-3 is cargo specific. Overexpression of another innexin, UNC-7::GFP, in GABAergic neurons also induced hsp-4 reporter expression, demonstrating that an excessive innexin load can activate the neuronal UPR. However, UNC-7-induced hsp-4 expression was not suppressed by loss of ergi-2 or ergi-3 (Fig 4E and 4F). ERGI-2 and ERGI-3 are therefore not general UPR components required for the response to innexin overexpression; instead, they selectively mediate the response to excessive UNC-9.

We further found that expression of GFP-tagged UNC-6 or VIT-2 under the control of the same unc-25 promoter activated the UPR, but neither response required ERGI-2 or ERGI-3 (Fig 4A–4D). These controls demonstrate that the selective dependence of the UNC-9::GFP-induced UPR on ERGI-2 and ERGI-3 cannot be attributed to the unc-25 promoter, GFP expression, or a general burden imposed by protein overexpression.

Our localization data provide independent support for this cargo-selective mechanism. In body-wall muscle cells, UNC-7::GFP formed puncta at cell-cell junctions, and this distribution was unaffected by ergi-2 or ergi-3 mutations (Fig 5J). Similarly, UNC-7::GFP formed puncta in the cell bodies and processes of DD/VD neurons, with no detectable alteration in either mutant (Fig 5K). By contrast, ERGI-2 and ERGI-3 were required for the proper localization of excessive UNC-9 in both muscle cells and GABAergic neurons. Thus, their selective requirement for UNC-9-induced UPR closely parallels their selective role in UNC-9 trafficking. These findings support a model in which ERGI-2 and ERGI-3 couple the handling of a particular membrane cargo to activation of the ER stress response.

Our results further indicate that ERGI-2 and ERGI-3 are not simply passive transport factors. In ergi-2 and ergi-3 mutants, excessive UNC-9 accumulated in ER-associated structures (Fig 5F), yet activation of the IRE-1-dependent UPR was markedly attenuated (Fig 1B). Accumulation of UNC-9 in the ER is therefore not, by itself, sufficient to generate the full stress response. Instead, ERGI-2 and ERGI-3 appear to actively couple UNC-9 recognition or trafficking to UPR activation. Their interactions with both UNC-9 and HSP-4 provide a plausible molecular basis for this coupling. Although precisely how these interactions influence IRE-1 activation remains to be established, our genetic, cell-biological, and biochemical evidence collectively supports an active role for ERGI-2 and ERGI-3 in cargo-associated UPR regulation.

Importantly, this response has a clear protective consequence. When ire-1 or xbp-1 was removed in animals overexpressing UNC-9, DD/VD neurons exhibited progressive, age-dependent loss (Guan et al., 2020). Thus, UPR activation in this model is not merely a reporter response to transgene expression; it is an adaptive mechanism required to preserve neuronal survival under chronic UNC-9 burden. Conversely, ERGI-2 and ERGI-3 are not required for the normal localization of endogenous UNC-9 in neurons. We therefore interpret their role as a conditional proteostatic safeguard that becomes particularly important when UNC-9 production exceeds the basal capacity of the neuronal secretory pathway, rather than as an obligatory component of constitutive UNC-9 trafficking.

We propose that the biological significance of this pathway lies in its ability to match ER homeostatic capacity to cargo-specific processing demand. Such demand could arise not only from increased synthesis but also from impaired assembly, trafficking, or turnover of multimeric membrane proteins. Innexins may present a particularly substantial challenge because they must be folded, oligomerized, and delivered correctly before assembling into gap-junction channels. A cargo-associated mechanism involving ERGI-2 and ERGI-3 could therefore allow neurons to engage a protective UPR before defects in membrane-protein handling produce broader proteostatic failure.

This mechanism may be especially important in neurons, which must continuously synthesize and deliver membrane proteins across long cellular processes while maintaining proteostasis throughout the lifetime of the animal. Although the endogenous physiological conditions that engage this pathway remain to be defined, our overexpression system reveals a biologically consequential stress-response mechanism that is difficult to detect under basal conditions.

Taken together, our findings support a model in which the secretory machinery acts as an active and cargo-selective modulator of UPR signaling. By linking the handling of excessive UNC-9 to HSP-4 and IRE-1-XBP-1 activation, ERGI-2 and ERGI-3 help convert a specific membrane-protein burden into a protective neuronal response. We have expanded the Discussion to clarify this physiological framework while explicitly acknowledging that the endogenous conditions under which this mechanism is engaged remain an important question for future investigation.

Reviewer #3: In this manuscript by Guan, Zhang, and colleagues, the authors used C. elegans to show that transgenic overexpression of a gap junction protein, UNC-9, induces UPR via COPII-associated proteins, ERGIC2 and 3. The authors used HSP-4/BiP and xbp-1 alternative splicing as markers of UPR activation to show that loss of ergi-2 and ergi-3 suppresses the UPR induced by UNC-9 overexpression. ergi-2 and 3 act upstream of UPR, as overexpression of ire-1 and xbp-1s can activate UPR in the ergi-2 and 3 mutants. Interestingly, the function of ergi-2 and 3 in inducing UPR appears to be very specific to UNC-9 overexpression, as UPR induced by UNC-6 or VIT-2 overexpression or Tunicamycin is unaffected in ergi-2 and 3 mutants. Using co-IP and mass spectrometry, they show that ERGI-2 and 3 bind HSP-4/BiP, and this interaction dissociates IRE-1 from BiP. ERGI-2 and 3 also coimmunoprecipitate with UNC-9. UNC-9::GFP puncta stuck in ER in ergi-2 and 3 mutants, which can be suppressed by xbp-1s or hsp-4 overexpression.

This work is a logical extension of the authors’ previous two publications in which they showed that 1) UNC-9::GFP overexpression induces UPR (Guan et al., Plos Genetics 2020), and 2) UNC-9::GFP localization depends on ERGI-2 and ERGI-3 (Guan et al., Traffic 2022). The present work connects these two works. The work is well executed, but is completely dependent on the artificial transgenic overexpression system.

1. Many of the observations have already been presented in their previous works. Throughout the manuscript, the authors do not properly cite their own prior work, making it difficult to assess the novelty of this work. The authors should describe their previous findings in the introduction in detail, and cite these papers wherever in the results section confirming their previous work.

Response: We thank the reviewer for this important suggestion. The present study is a logical extension of our two previous publications. In our earlier work, we showed that UNC-9::GFP overexpression in neurons induces the UPR (Guan et al., 2020). We subsequently demonstrated that the proper localization of UNC-9::GFP depends on the early secretory-pathway proteins ERGI-2 and ERGI-3 (Guan et al., 2022).

The current study builds upon and connects these observations by demonstrating that ERGI-2 and ERGI-3 couple UNC-9 trafficking to activation of the IRE-1-XBP-1 pathway. It further provides genetic, cell-biological, and biochemical evidence for a cargo-selective mechanism through which early secretory-pathway components regulate the neuronal UPR.

Following the reviewer’s suggestion, we have revised the Introduction to describe our previous findings more clearly and distinguish them from the advances made in the present study. We have also added the appropriate citations throughout the Results wherever earlier observations are reproduced, confirmed, or used as the basis for new experiments. These revisions should make the relationship between our previous studies and the novelty of the present work substantially clearer.

2. The authors described that they identified ergi-2 and ergi-3 from a genetic screening of reduced hsp-4 expression. Please describe how screening was conducted in the method section, and nature of alleles they identified. Why did the authors use the ergi-2(xd84) allele that they isolated from their previous screening (Guan et al., Traffic 2022) and an allele from the Million mutation project?

Response: We thank the reviewer for raising this point, and we apologize that our original description created the impression that ergi-2 and ergi-3 were identified through a new screen for reduced hsp-4 expression. This was not the case.

The ergi-2(xd84) allele was originally isolated in an EMS-based forward genetic screen for mutants with defective UNC-9-containing gap-junction formation in C. elegans body-wall muscle cells (Guan et al., 2022). We subsequently examined an ergi-3 allele obtained from the Million Mutation Project and found that it produced a similar defect in UNC-9 localization and gap-junction formation in muscle cells. These findings, including the origins and molecular characteristics of the alleles, were reported in Guan et al (Guan et al., 2022). We used these alleles in the present study because they had already been established as genetic tools for investigating the roles of ERGI-2 and ERGI-3 in UNC-9 trafficking.

In parallel, our earlier study showed that UNC-9::GFP overexpression in DD/VD neurons induces ER stress and activates the IRE-1-XBP-1 branch of the UPR (Guan et al., 2020). We therefore introduced the previously characterized ergi-2 and ergi-3 alleles into animals overexpressing UNC-9::GFP in DD/VD neurons to determine how ERGI-2/3-dependent UNC-9 trafficking influences the neuronal ER UPR.

As expected from their roles in UNC-9 trafficking, loss of either ergi-2 or ergi-3 disrupted the distribution of UNC-9::GFP along DD/VD neuronal processes and caused the protein to accumulate in large structures within the neuronal cell bodies. Because impaired ER-to-Golgi transport and increased ER protein retention commonly activate the UPR, we initially expected Phsp-4::mCherry expression to be maintained or further increased in these mutants. Unexpectedly, however, Phsp-4::mCherry fluorescence was markedly reduced (Fig 1B). This observation provided the starting point for the present study: why does increased UNC-9 accumulation in ergi-2 and ergi-3 mutants attenuate, rather than enhance, the UPR?

To eliminate this confusion in the revised manuscript, we have made the following changes: 1) We removed wording suggesting that ergi-2 and ergi-3 were identified through a screen for reduced hsp-4 expression; 2) We clarified why these previously characterized alleles were selected for the current neuronal UPR experiments; 3) We revised the Introduction and Results to distinguish clearly between our previously published findings and the unexpected observation that initiated the present study, with the appropriate citations to Guan et al. (Guan et al., 2020) and Guan et al. (Guan et al., 2022).

These revisions should make the origin of the alleles, the rationale for their use, and the progression from our previous studies to the present work much clearer.

3. While it is interesting that the function of ergi-2 and ergi-3 is very specific to UNC-9 overexpression, it is possible that everything the authors observe is specific to the exact transgene they used (Punc-25::UNC-9::GFP). Do ergi-2 and ergi-3 activate UPR when other innexin genes (such as unc-7) are overexpressed? Is it dependent on the specific promoter the authors used to overexpress UNC-9? UNC-9::GFP has been shown to be a gain-of-function form. Does untagged UNC-9 also cause ergi-2/3-dependent UPR?

Response: We thank the reviewer for these insightful questions. To determine whether our observations are specific to the Punc-25::UNC-9::GFP transgene, we performed additional experiments addressing the identity of the overexpressed protein, the GFP tag, and the promoter used.

First, we examined UNC-7, another well-characterized innexin expressed in both neurons and muscle cells. Overexpression of UNC-7::GFP in DD/VD neurons using the same unc-25 promoter significantly increased Phsp-4::mCherry fluorescence (Fig 4E and 4F), demonstrating that an excessive load of another innexin can also activate the UPR. However, introduction of either the ergi-2 or ergi-3 mutation did not suppress UNC-7::GFP-induced hsp-4 expression (Fig 4E and 4F). Thus, ERGI-2 and ERGI-3 are not generally required for UPR activation caused by innexin overexpression; their requirement is selective for the response induced by UNC-9.

This selectivity is also supported by our localization analyses. In body-wall muscle cells, UNC-7::GFP displayed a punctate distribution at cell-cell junctions, and this pattern was unaffected by loss of ergi-2 or ergi-3 (Fig 5J). Similarly, in DD/VD neurons, the UNC-7::GFP puncta within cell bodies and along neuronal processes were indistinguishable among wild-type, ergi-2, and ergi-3 animals(Fig 5K). By contrast, ERGI-2 and ERGI-3 are required for the proper localization of UNC-9 in both muscle cells and GABAergic neurons (Fig 5A) (Guan et al., 2022). The cargo-selective effects on both localization and UPR activation therefore support a model in which ERGI-2 and ERGI-3 couple the trafficking of UNC-9, but not UNC-7, to the UPR.

We also considered whether the response could be produced nonspecifically by the unc-25 promoter or the GFP moiety. Expression of GFP-tagged UNC-7, UNC-6, or VIT-2 from the same unc-25 promoter also activated the UPR, but these responses were not dependent on ERGI-2 or ERGI-3 (Fig 4A-4F). Therefore, the selective ERGI-2/3 dependence of the response to UNC-9::GFP cannot readily be attributed to the unc-25 promoter, GFP expression, or a general protein-overexpression burden.

We were concerned that the UPR effects observed with *ergi-2* and *ergi-3* might actually stem from an abnormal GFP fusion protein of UNC-9, rather than from UNC-9 itself. To address this, we reevaluated our findings using untagged UNC-9. Overexpression of untagged UNC-9 in DD/VD neurons was sufficient to induce hsp-4 expression. Importantly, this induction was reduced in both ergi-2 and ergi-3 mutants (Fig 4G-4H). These results demonstrate that an increased load of UNC-9 itself activates an ERGI-2/3-dependent UPR and that this effect is not an artifact specific to the UNC-9::GFP fusion.

Finally, we investigated whether the response depends specifically on expression from the unc-25 promoter. We examined UNC-9::GFP expressed in a subset of interneurons using the unc-53 promoter. UNC-9::GFP showed a punctate distribution in the cell bodies and processes of these neurons. As reported previously, loss of ire-1 or xbp-1 disrupted this distribution and caused UNC-9::GFP to become diffuse within the cell-body region (Fig 5A-5B) (Guan et al., 2020), indicating that the IRE-1-XBP-1 pathway contributes to the handling of excessive UNC-9 in these interneurons.

However, Punc-53::UNC-9::GFP did not produce a detectable increase in Phsp-4::mCherry fluorescence (Response Figure 1). This difference may reflect a lower UNC-9 expression level from Punc-53 than from Punc-25, differences in the proteostatic capacity of the targeted neurons, or both. Thus, our results do not indicate that the unc-25 promoter itself triggers the response. Instead, they suggest that the detectable magnitude of UNC-9-induced UPR activation depends on UNC-9 protein load and cellular context. At a relatively modest expression level, the contribution of IRE-1 and XBP-1 to UNC-9 handling may be detectable without sufficient activation to produce a measurable hsp-4 transcriptional response.

Collectively, these findings support the conclusion that ERGI-2 and ERGI-3 selectively connect an increased UNC-9 processing burden to the neuronal UPR, rather than responding nonspecifically to the promoter, GFP tag, or general protein overexpression. We have incorporated these new data and clarifications into the revised Results and Discussion.

Response Figure 1: Overexpression of UNC-9::GFP under the P*unc‑53* promoter does not induce *hsp‑4* expression.

4. Suppression of transgenic over-expression phenotypes can be caused by a reduction of transgene expression. The authors should quantify UNC-9::GFP expression levels across different genetic backgrounds and rule out the possibility that the phenotypic suppression is due to reduced transgene expression.

Response: We thank the reviewer for this important suggestion. To determine whether the suppression of the UNC-9::GFP-induced phenotype in ergi-2 and ergi-3 mutants resulted from reduced transgene expression, we quantified unc-9::gfp transcript levels by RT-qPCR. We detected no significant difference in unc-9::gfp mRNA abundance among the wild-type, ergi-2, and ergi-3 backgrounds. Thus, the attenuation of UPR activation in these mutants cannot be explained by reduced transcription of the Punc-25::UNC-9::GFP transgene. These data have now been included in S3 Fig and described in the revised Results and Methods sections.

5. Related to the above, the authors showed that overexpression of hsp-4 or xbp-1s can bypass the requirement of ergi-2 and ergi-3 on UNC-9 localization phenotype (figure 8). The authors used the same promoter (Punc-25) for this experiment, and it is possible that an excessive amount of this promoter dampens unc-9 expression, thereby reducing the amount of UNC-9 that restores proper localization. The authors should use different promoters for this experiment.

Response: We thank the reviewer for raising this important concern. To exclude the possibility that the use of multiple Punc-25-driven transgenes reduces UNC-9::GFP expression through promoter competition, we repeated these experiments using the unc-47 promoter, which also drives expression specifically in DD/VD GABAergic neurons.

Expression of either XBP-1s or HSP-4 from the unc-47 promoter significantly reduced the large UNC-9::GFP accumulations observed in ergi-2 and ergi-3 mutants (Fig 8A). These results reproduce the effects obtained with the corresponding Punc-25-driven constructs and therefore demonstrate that the rescue is not caused by competition between identical promoters.

Together, these findings support the conclusion that increased XBP-1s or HSP-4 eliminate excessive UNC-9 in ergi-2 and ergi-3 mutants, rather than simply reducing UNC-9::GFP expression through promoter competition. We have included these new data in Fig 8 and revised the corresponding Results and Methods sections.
