# Supplementary material for: ER-to-Golgi transport machinery promotes the excessive cargo-triggered unfolded protein response in C. elegans
Source: PLoS Genet. 2026 Sep 8;22(9):e1012301. doi: 10.1371/journal.pgen.1012301 (PMC13568704; doi:10.1371/journal.pgen.1012301)
Supplement: Peer review history

# Peer review: Decision Letter 0

**DOI:** 10.1371/journal.pgen.1012301.r001

**Authors:** Andrew Chisholm, Fengwei Yu

11 May 2026

PGENETICS-D-26-00349

ER-to-Golgi transport machinery promotes the excessive cargo-triggered unfolded protein response

PLOS Genetics

Dear Dr. Ding,

Thank you for submitting your manuscript to PLOS Genetics. After careful consideration, we feel that it has merit but does not fully meet PLOS Genetics's publication criteria as it currently stands. Therefore, we invite you to submit a revised version of the manuscript that addresses the points raised during the review process.

We and the reviewers find significant experimental work is essential for the current manuscript to be viable  at PLOS Genetics. Any revised ms must incorporate new data that address the major reviewer concerns, minimally tests of over expression of untagged UNC-9, tests of other innexins, or tests of other promoters.

Please submit your revised manuscript by Jul 10 2026 11:59PM. If you will need more time than this to complete your revisions, please reply to this message or contact the journal office at plosgenetics@plos.org. When you're ready to submit your revision, log on to https://www.editorialmanager.com/pgenetics/ and select the 'Submissions Needing Revision' folder to locate your manuscript file.

Please include the following items when submitting your revised manuscript:

* A letter that responds to each point raised by the editor and reviewer(s). You should upload this letter as a separate file labeled 'Response to Reviewers'. This file does not need to include responses to any formatting updates and technical items listed in the 'Journal Requirements' section below.

* A marked-up copy of your manuscript that highlights changes made to the original version. You should upload this as a separate file labeled 'Revised Manuscript with Track Changes'.

* An unmarked version of your revised paper without tracked changes. You should upload this as a separate file labeled 'Manuscript'.

If you would like to make changes to your financial disclosure, competing interests statement, or data availability statement, please make these updates within the submission form at the time of resubmission. Guidelines for resubmitting your figure files are available below the reviewer comments at the end of this letter.

As the corresponding author, your ORCID iD is verified in the submission system and will appear in the published article. PLOS supports the use of ORCID, and we encourage all coauthors to register for an ORCID iD and use it as well. Please encourage your coauthors to verify their ORCID iD within the submission system before final acceptance, as unverified ORCID iDs will not appear in the published article. Only the individual author can complete the verification step; PLOS staff cannot verify ORCID iDs on behalf of authors.

We look forward to receiving your revised manuscript.

Kind regards,

Andrew D. Chisholm

Academic Editor

PLOS Genetics

Fengwei Yu

Section Editor

PLOS Genetics

Aimée Dudley

Editor-in-Chief

PLOS Genetics

Anne Goriely

Editor-in-Chief

PLOS Genetics

**Journal Requirements:**

1) Please ensure that the CRediT author contributions listed for every co-author are completed accurately and in full.

At this stage, the following Authors/Authors require contributions: Liying Guan, Tong Zhang, Zhigao Zhan, Yingchun Wang, Xun Huang, and Mei Ding. Please ensure that the full contributions of each author are acknowledged in the "Add/Edit/Remove Authors" section of our submission form.

The list of CRediT author contributions may be found here: https://journals.plos.org/plosgenetics/s/authorship#loc-author-contributions

2) We ask that a manuscript source file is provided at Revision. Please upload your manuscript file as a .doc, .docx, .rtf or .tex. If you are providing a .tex file, please upload it under the item type u2018LaTeX Source Fileu2019 and leave your .pdf version as the item type u2018Manuscriptu2019.

3) Please provide an Author Summary. This should appear in your manuscript between the Abstract (if applicable) and the Introduction, and should be 150-200 words long. The aim should be to make your findings accessible to a wide audience that includes both scientists and non-scientists. Sample summaries can be found on our website under Submission Guidelines:

https://journals.plos.org/plosgenetics/s/submission-guidelines#loc-parts-of-a-submission

4) Please upload all main figures as separate Figure files in .tif or .eps format. For more information about how to convert and format your figure files please see our guidelines:

https://journals.plos.org/plosgenetics/s/figures

5) We have noticed that you have uploaded Supporting Information files, but you have not included a list of legends. Please add a full list of legends for your Supporting Information files after the references list.

6) Please amend your detailed Financial Disclosure statement. This is published with the article. It must therefore be completed in full sentences and contain the exact wording you wish to be published.

State what role the funders took in the study. If the funders had no role in your study, please state: "The funders had no role in study design, data collection and analysis, decision to publish, or preparation of the manuscript.".

If you did not receive any funding for this study, please simply state: u201cThe authors received no specific funding for this work.u201d

**Reviewers' comments:**

Reviewer's Responses to Questions

**Comments to the Authors:**

**Please note here if the review is uploaded as an attachment.**

Reviewer #1: Guan, Ding and colleagues explore how C. elegans vesicular trafficking machinery regulates ER stress responses induced by neuronal overexpression of gap junction proteins. Built on a prior study published by the same group of authors a few years ago, they investigated how overexpression of the C. elegans innexin UNC-9 in the GABAergic DD/VD motor neurons elicited ER unfolded protein responses (UPR-ER). They focused on ERGI-2 and ERGI-3, two proteins of the ER-Golgi trafficking pathway. Through a series of genetic, imaging and biochemical studies, they provided evidence pointing to a role of ERGI-2 and ERGI-3 dedicated specifically to UPR-ER caused by the expression of UNC-9, but not those by expressing other secreted proteins (netrin, vitellogenin) or by stressors (Tunicamycin, heat stress). Several lines of evidence suggest a model in which ERGI-2 and ERGI-3 recruit HSP-4, the ER stress chaperone, which frees the ER-resident kinase/endonuclease IRE-1 for splicing the xbp-1s messenger RNA. The findings that ER-Golgi trafficking proteins regulate UPR-ER are consistent with recent findings that Golgi trafficking proteins are involved in the regulation of ER stress responses. Most of the experiments were properly carried out, and the quantification of data was rigorous. Together these observations provide interesting insights on the mechanisms of proteostasis in the neurons. Below are issues that need to be addressed to further consolidate the major conclusions of the study. Below are key issues that the authors should address to further substantiate the major conclusions of the paper.

Major comments:

1. The nature of UNC-9 overexpression in the GABAergic neurons needs to be clarified, including the following: (1) Does expression of other gap junction proteins, such as UNC-7, show similar effects? (2) Is induction of neuron-autonomous UPR-ER by UNC-9 specific to GABAergic neurons, or does it also occur in other neuronal types?

2. The authors hypothesized that ERGI-2 and ERGI-3 are dedicated to mitigating ER stress triggered by UNC-9 overexpression. Data supporting this model includes biochemical evidence that ERGI-2 and ERGI-3 interact with UNC-9 and HSP-4 by forming protein complexes. One possibility is that ERGI-2 and ERGI-3 activate IRE-1 by removing HSP-4 away from it. However, one can imagine that such mechanisms will not be specific for ER stress induced by UNC-9, as HSP-4-IRE-1 is a core component of UPR-ER for many different stressors, and ERGI-2 and ERGI-3 can bind HSP-4 without UNC-9. How does such a model explain the specificity of ERGI-2/3 to UPR-ER induced by UNC-9, but not other protein stressors? Could ERGI-2/3 overexpression enhance the ability of the neurons to cope with ER stress induced by non-UNC-9 factors, such as Tunicamycin, heat or other secreted or misfolded proteins?

3. Although unc-9 is expressed in VD and DD, according to the C. elegans single neuron gene expression dataset, the expression level is low (212 (rank: 67) in VD and 181 (rank: 73) in DD). In contrast, they are expressed in several non-neuronal cells, and in one of the authors’ prior papers, ergi-2 and erg-3 were shown to act in the muscles. Therefore, the authors should examine whether ergi-2 and ergi-3 act cell-autonomously in VD/DD neurons to modulate ER Stress response caused by UNC-9 overexpression.

4. The sensor for xbp-1 splicing is interesting, but using the disappearance of signals as evidence of a positive event is sometimes confounded by technical issues that lead to lack of signals unrelated to the regulatory mechanisms. Could the authors design an alternative sensor such that baseline mCherry expression is prevented by an out-of-frame design, and mCherry is in the reading frame when the xbp-1 short intron is removed by IRE-1? This will ensure that expression of mCherry is indeed triggered by xbp-1 splicing.

5. The imaging study of excessive UNC-9 in subcellular compartments of different genotypes needs further substantiation (Figure 5 and Figure S2). First, please provide images of UNC-9::GFP with markers for ER (CYTB-5::mCherry), chaperones (RFP::HSP-4), early endosomes (mCherry::RAB-5), late endosomes (mCherry::RAB-7), autophagosomes (mCherry::LGG-1) and lysosomes (LAAT-1::mCherry) in the wild type. Second, in both ergi-2 and ergi-3 mutants, UNC-9::GFP in the neuronal cell bodies appears as two big (perinuclear) blobs, whereas CYTB-5::mCherry signal is diffuse in the cytosol (in the extensive ER network?). Therefore, it is possible that UNC-9::GFP in these mutants is aggregated in another membrane organelle that is close to the cell nucleus and intimately associated with ER, such as Golgi. A Golgi marker should be used to further clarify the nature of these aggregates.

6. Does ER stress caused by UNC-9 overload impair neuronal health or functions? This important question is surprisingly missed in the current and prior publications.

7. The authenticity of mCherry::LGG-1 puncta as autophagosomes needs to be further confirmed. Mutating the terminal glycine (G116) should abolish LGG-1 clustering on autophagosomes and therefore diminish puncta formation. This needs to be tested. In addition, the criterion of mCherry::LGG-1 quantification needs to be clearly defined (diameter, for example).

General:

1. The authors should mention C. elegans in the title, Abstract and Introduction, and make it clear that the findings described in this paper were made using this nematode.

2. The Introduction contains too little information and does not provide an accurate and suitable context for the readers to understand why the study fills the gaps in our understanding of ER stress responses. Please substantiate this section.

3. Were Figures 3A and 3C processed for contrast enhancement? Some of the images look oversaturated for pixel values.

4. In the Materials and Methods section, please define how cell bodies with hsp-4::mCherry expression or UNC-9 puncta were defined. Some quantitative criteria of calling UNC-9 puncta, positive mCherry expression or UNC-9 aggregates need to be provided.

Reviewer #2: In this manuscript, the authors investigate the role of COPII-associated proteins ERGIC2 and ERGIC3 in gap junction overexpression–mediated IRE1–XBP1 activation. Overall, this is an interesting study that suggests a potentially novel function of COPII-associated proteins beyond cargo trafficking. The proposed signaling pathway appears to be activated by UNC-9::GFP overexpression; however, several key control experiments are missing. In addition, the biological relevance of this pathway under physiological conditions remains unclear.

1. Use of UNC-9::GFP

UNC-9::GFP may not fully reflect the behavior of native gap junction proteins. Previous work (e.g., Chen et al., 2007) showed that UNC-9::GFP can form constitutively open gap junction channels, while other studies suggest that its overexpression can disrupt normal gap junction function and mimic unc-9 loss-of-function phenotypes. Therefore, it is important to reevaluate these observations using untagged UNC-9 to ensure that the reported phenotypes and signaling are not artifacts caused by an abnormal fusion protein. Additionally, UNC-9 has been reported to possess channel-independent functions (Mizumoto, 2022). As the manuscript suggests that gap junction activity is responsible for activating the pathway, it is important to determine whether the channel function of UNC-9 is specifically required for the observed phenotypes.

2. Specificity to UNC-9 versus other innexins

In addition to unc-9, multiple innexins are expressed in neurons. It is unclear whether the observed phenotype is specific to UNC-9 or represents a more general property of innexins. This could be addressed by overexpressing other neuronal innexins, such as unc-7 or inx-1, and assessing whether they similarly activate the IRE1–XBP1 pathway.

3. Cell-type specificity

It is unclear whether this phenotype is neuron-specific or reflects a broader cellular mechanism. Since unc-9 is expressed in both neurons and muscles, it would be informative to test whether overexpression of UNC-9 in muscle cells also activates IRE1–XBP1 signaling.

4. Physiological relevance

The proposed signaling pathway appears to be triggered by UNC-9::GFP overexpression, and the requirement for ERGIC2/ERGIC3 also seems to depend specifically on this condition. As such, the physiological relevance of this pathway remains unclear. While it may be technically challenging to address this directly with current tools, the authors should more thoroughly discuss the potential biological significance and contexts in which this signaling mechanism might operate.

Reviewer #3: In this manuscript by Guan, Zhang, and colleagues, the authors used C. elegans to show that transgenic overexpression of a gap junction protein, UNC-9, induces UPR via COPII-associated proteins, ERGIC2 and 3. The authors used HSP-4/BiP and xbp-1 alternative splicing as markers of UPR activation to show that loss of ergi-2 and ergi-3 suppresses the UPR induced by UNC-9 overexpression. ergi-2 and 3 act upstream of UPR, as overexpression of ire-1 and xbp-1s can activate UPR in the ergi2 and 3 mutants. Interestingly, the function of ergi-2 and 3 in inducing UPR appears to be very specific to UNC-9 overexpression, as UPR induced by UNC-6 or VIT-2 overexpression or Tunicamycin is unaffected in ergi-2 and 3 mutants. Using co-IP and mass spectrometry, they show that ERGI-2 and 3 bind HSP-4/BiP, and this interaction dissociates IRE-1 from BiP. ERGI-2 and 3 also coimmunoprecipitate with UNC-9. UNC-9::GFP puncta stuck in ER in ergi-2 and 3 mutants, which can be suppressed by xbp-1s or hsp-4 overexpression.

This work is a logical extension of the authors’ previous two publications in which they showed that 1) UNC-9::GFP overexpression induces UPR (Guan et al., Plos Genetics 2020), and 2) UNC-9::GFP localization depends on ERGI-2 and ERGI-3 (Guan et al., Traffic 2022). The present work connects these two works. The work is well executed, but is completely dependent on the artificial transgenic overexpression system.

1. Many of the observations have already been presented in their previous works. Throughout the manuscript, the authors do not properly cite their own prior work, making it difficult to assess the novelty of this work. The authors should describe their previous findings in the introduction in detail, and cite these papers wherever in the results section confirming their previous work.

2. The authors described that they identified ergi-2 and ergi-3 from a genetic screening of reduced hsp-4 expression. Please describe how screening was conducted in the method section, and nature of alleles they identified. Why did the authors use the ergi-2(xd84) allele that they isolated from their previous screening (Guan et al., Traffic 2022) and an allele from the Million mutation project?

3. While it is interesting that the function of ergi-2 and ergi-3 is very specific to UNC-9 overexpression, it is possible that everything the authors observe is specific to the exact transgene they used (Punc-25::UNC-9::GFP). Do ergi-2 and ergi-3 activate UPR when other innexin genes (such as unc-7) are overexpressed? Is it dependent on the specific promoter the authors used to overexpress UNC-9? UNC-9::GFP has been shown to be a gain-of-function form. Does untagged UNC-9 also cause ergi-2/3-dependent UPR?

4. Suppression of transgenic over-expression phenotypes can be caused by a reduction of transgene expression. The authors should quantify UNC-9::GFP expression levels across different genetic backgrounds and rule out the possibility that the phenotypic suppression is due to reduced transgene expression.

5. Related to the above, the authors showed that overexpression of hsp-4 or xbp-1s can bypass the requirement of ergi-2 and ergi-3 on UNC-9 localization phenotype (figure 8). The authors used the same promoter (Punc-25) for this experiment, and it is possible that an excessive amount of this promoter dampens unc-9 expression, thereby reducing the amount of UNC-9 that restores proper localization. The authors should use different promoters for this experiment.

**********

**Have all data underlying the figures and results presented in the manuscript been provided?**

Large-scale datasets should be made available via a public repository as described in the *PLOS Genetics*  data availability policy, and numerical data that underlies graphs or summary statistics should be provided in spreadsheet form as supporting information.

Reviewer #1: Yes

Reviewer #2: Yes

Reviewer #3: Yes

**********

PLOS authors have the option to publish the peer review history of their article (what does this mean?). If published, this will include your full peer review and any attached files.

If you choose “no”, your identity will remain anonymous but your review may still be made public.

**Do you want your identity to be public for this peer review?**  For information about this choice, including consent withdrawal, please see our Privacy Policy.

Reviewer #1: No

Reviewer #2: No

Reviewer #3: No

[NOTE: If reviewer comments were submitted as an attachment file, they will be attached to this email and accessible via the submission site. Please log into your account, locate the manuscript record, and check for the action link "View Attachments". If this link does not appear, there are no attachment files.]

**Figure resubmission:**

**Reproducibility:**

To enhance the reproducibility of your results, we recommend that authors of applicable studies deposit laboratory protocols in protocols.io, where a protocol can be assigned its own identifier (DOI) such that it can be cited independently in the future. Additionally, PLOS ONE offers an option to publish peer-reviewed clinical study protocols. Read more information on sharing protocols at https://plos.org/protocols?utm_medium=editorial-email&utm_source=authorletters&utm_campaign=protocols

# Author comment: Author response to Decision Letter 1

**DOI:** 10.1371/journal.pgen.1012301.r002

5 Aug 2026

Attachment Submitted filename: Response to reviewers.docx

# Peer review: Decision Letter 1

**DOI:** 10.1371/journal.pgen.1012301.r003

**Authors:** Andrew Chisholm, Fengwei Yu

25 Aug 2026

Dear Dr Ding,

We are pleased to inform you that your manuscript entitled "ER-to-Golgi transport machinery promotes the excessive cargo-triggered unfolded protein response in C . elegans" has been editorially accepted for publication in PLOS Genetics. Congratulations!

Before your submission can be formally accepted and sent to production you will need to complete our formatting changes, which you will receive in a follow up email. Please be aware that it may take several days for you to receive this email; during this time no action is required by you. Please note: the accept date on your published article will reflect the date of this provisional acceptance, but your manuscript will not be scheduled for publication until the required changes have been made.

Once your paper is formally accepted, an uncorrected proof of your manuscript will be published online ahead of the final version, unless you’ve already opted out via the online submission form. If, for any reason, you do not want an earlier version of your manuscript published online or are unsure if you have already indicated as such, please let the journal staff know immediately at plosgenetics@plos.org.

In the meantime, please log into Editorial Manager at https://www.editorialmanager.com/pgenetics/, click the "Update My Information" link at the top of the page, and update your user information to ensure an efficient production and billing process. Note that PLOS requires an ORCID iD for all corresponding authors. Therefore, please ensure that you have an ORCID iD and that it is validated in Editorial Manager. To do this, go to ‘Update my Information’ (in the upper left-hand corner of the main menu), and click on the Fetch/Validate link next to the ORCID field.  This will take you to the ORCID site and allow you to create a new iD or authenticate a pre-existing iD in Editorial Manager.

If you have a press-related query, or would like to know about making your underlying data available (as you will be aware, this is required for publication), please see the end of this email. If your institution or institutions have a press office, please notify them about your upcoming article at this point, to enable them to help maximise its impact. Inform journal staff as soon as possible if you are preparing a press release for your article and need a publication date.

Thank you again for supporting open-access publishing; we are looking forward to publishing your work in PLOS Genetics!

Yours sincerely,

Andrew D. Chisholm

Academic Editor

PLOS Genetics

Fengwei Yu

Section Editor

PLOS Genetics

Aimée Dudley

Editor-in-Chief

PLOS Genetics

Anne Goriely

Editor-in-Chief

PLOS Genetics

www.plosgenetics.org

BlueSky: @plos.bsky.social

----------------------------------------------------

Comments from the reviewers (if applicable):

Reviewer's Responses to Questions

**Comments to the Authors:**

**Please note here if the review is uploaded as an attachment.**

Reviewer #1: The authors had addressed all the issues that I raised in the initial review with either additional experiments or revision of the manuscript. Specifically, they clarified that (1) ERGI-2 and ERGI-3 act autonomously in DD and VD neurons to trigger ER unfolded protein responses elicited by overexpression of UNC-9 in these neurons; (2) expression of UNC-7, another gap junction protein, induced UPR-ER in an ergi-2/3-independent manner; (3) peri-nuclear aggregates of ERGI-2/3 are not associated with Golgi, and (4) UPR-ER triggered by UNC-9 overexpression cannot be replicated in neurons other than VD/DD, but there may be technical explanations for this. Along with a number of other textual revisions, these additional results substantially improve the quality and conceptual framework of the paper. Overall, it elaborates the molecular mechanisms of UPR-ER, showing that there is further molecular specificity for the stress responses induced by different types of proteins. This could open new directions for future research of proteostatic stress. I do not have further comments, and would like congratulate the authors for their solid work.

Reviewer #2: the authors have addressed all my concerns.

Reviewer #3: the authors addressed questions and concerns raised by this reviewer thoroughly.

**********

**Have all data underlying the figures and results presented in the manuscript been provided?**

Large-scale datasets should be made available via a public repository as described in the *PLOS Genetics*  data availability policy, and numerical data that underlies graphs or summary statistics should be provided in spreadsheet form as supporting information.

Reviewer #1: Yes

Reviewer #2: Yes

Reviewer #3: Yes

**********

PLOS authors have the option to publish the peer review history of their article (what does this mean?). If published, this will include your full peer review and any attached files.

If you choose “no”, your identity will remain anonymous but your review may still be made public.

**Do you want your identity to be public for this peer review?**  For information about this choice, including consent withdrawal, please see our Privacy Policy.

Reviewer #1: No

Reviewer #2: No

Reviewer #3: No

----------------------------------------------------

**Data Deposition**

If you have submitted a Research Article or Front Matter that has associated data that are not suitable for deposition in a subject-specific public repository (such as GenBank or ArrayExpress), one way to make that data available is to deposit it in the Dryad Digital Repository. As you may recall, we ask all authors to agree to make data available; this is one way to achieve that. A full list of recommended repositories can be found on our website.

The following link will take you to the Dryad record for your article, so you won't have to re‐enter its bibliographic information, and can upload your files directly:

http://datadryad.org/submit?journalID=pgenetics&manu=PGENETICS-D-26-00349R1

More information about depositing data in Dryad is available at http://www.datadryad.org/depositing. If you experience any difficulties in submitting your data, please contact help@datadryad.org for support.

Additionally, please be aware that our data availability policy requires that all numerical data underlying display items are included with the submission, and you will need to provide this before we can formally accept your manuscript, if not already present.

----------------------------------------------------

**Press Queries**

If you or your institution will be preparing press materials for this manuscript, or if you need to know your paper's publication date for media purposes, please inform the journal staff as soon as possible so that your submission can be scheduled accordingly. Your manuscript will remain under a strict press embargo until the publication date and time. This means an early version of your manuscript will not be published ahead of your final version. PLOS Genetics may also choose to issue a press release for your article. If there's anything the journal should know or you'd like more information, please get in touch via plosgenetics@plos.org.

# Editor report: Acceptance letter

**DOI:** 10.1371/journal.pgen.1012301.r004

**Authors:** Andrew Chisholm, Fengwei Yu

PGENETICS-D-26-00349R1

ER-to-Golgi transport machinery promotes the excessive cargo-triggered unfolded protein response in C. elegans

Dear Dr Ding,

We are pleased to inform you that your manuscript entitled "ER-to-Golgi transport machinery promotes the excessive cargo-triggered unfolded protein response in C. elegans" has been formally accepted for publication in PLOS Genetics! Your manuscript is now with our production department and you will be notified of the publication date in due course.

The corresponding author will soon be receiving a typeset proof for review, to ensure errors have not been introduced during production. Please review the PDF proof of your manuscript carefully, as this is the last chance to correct any errors. Please note that major changes, or those which affect the scientific understanding of the work, will likely cause delays to the publication date of your manuscript.

Soon after your final files are uploaded, unless you have opted out or your manuscript is a front-matter piece, the early version of your manuscript will be published online. The date of the early version will be your article's publication date. The final article will be published to the same URL, and all versions of the paper will be accessible to readers.

For Research Articles, you will receive an invoice from PLOS for your publication fee after your manuscript has reached the completed accept phase. If you receive an email requesting payment before acceptance or for any other service, this may be a phishing scheme. Learn how to identify phishing emails and protect your accounts at https://explore.plos.org/phishing.

Thank you again for supporting PLOS Genetics and open-access publishing. We are looking forward to publishing your work!

With kind regards,

Sharmila Kamatchi

PLOS Genetics

On behalf of:

The PLOS Genetics Team

Carlyle House, Carlyle Road, Cambridge CB4 3DN | United Kingdom

plosgenetics@plos.org | +44 (0) 1223-442823

plosgenetics.org | Twitter: @PLOSGenetics
